# A rapid, site-selective and efficient route to the dual modification of DARPins†
†Electronic supplementary information (ESI) available: LC-MS spectra for all reactions with proteins described herein. See DOI: 10.1039/c4cc00053f



**DOI:** 10.1039/c4cc00053f

**Published:** 2014-04-01

**Authors:** Paul Moody, Vijay Chudasama, Ramiz I. Nathani, Antoine Maruani, Stephen Martin, Mark E. B. Smith, Stephen Caddick

**Affiliations:** a Department of Chemistry , University College London , 20 Gordon Street , London , WC1H 0AJ , UK . Email: vpenterprise@ucl.ac.uk ; Tel: +44 (0)20 7679 7538; b MRC National Institute for Medical Research , The Ridgeway , Mill Hill , London , NW7 1AA , UK

## Abstract

Designed ankyrin repeat proteins (DARPins) are valuable tools in both biochemistry and medicine. Herein we describe a rapid, simple method for the dual modification of DARPins by introduction of cysteine mutations at specific positions that results in a vast difference in their thiol nucleophilicity, allowing for clean sequential modification.

## Abstract 2

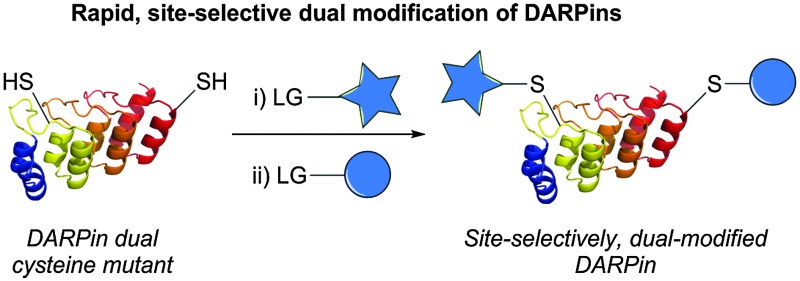
Herein we describe a rapid, simple method for dual modification of DARPins by introduction of cysteine mutations at specific positions that results in a vast difference in their thiol nucleophilicity, allowing for sequential modification.

A leading alternative to antibody proteins for binding to molecular targets are designed ankyrin repeat proteins (DARPins),^1^ which are small (∼15 kDa), thermally stable,^2^ and routinely expressed in *E. coli* in high yields. The ease with which DARPins can be genetically manipulated, expressed and purified has made them attractive tools for development in both academia and industry.^3^ DARPins are small, single-domain proteins, which consist of repeated structural units.^4,5^ DARPins contain at least 1 internal repeat, flanked by N- and C-terminal capping helices to prevent aggregation.^4,5^ These repeats form two antiparallel α-helices, and a β-hairpin. The long axes of the helices and the β-hairpin are perpendicular, which forms a pocket that is used as a binding site. In DARPins that have been selected to bind to a designated target, the residues that do not form the binding site are conserved, whereas the residues that form the binding site are derived from randomised sequences.^3^

Over the years, a plethora of different reagents for increasing the functionality of proteins have become available. These include: reagents for PEGylation for increased *in vivo* half-life,^6^ drugs^7^ or photosensitive reagents for targeted therapy,^8^ fluorophores for in-cell and *in vivo* imaging,^9^ MRI contrast agents,^10^ radiolabels,^11^ electrophiles for covalent attachment to protein targets,^12^ and mimics of post-translational modification.^13^ Despite the substantial toolbox of reagents that could be applied to functionalise proteins, site-selective addition of multiple reagents onto the same protein remains a significant challenge.^14^

A common strategy for the controlled, selective addition of multiple functional groups is to differentially label protein termini using inteins,^15^ sortase^16^ or native chemical ligation^17^ labelling strategies. These strategies, however, are commonly limited to the labelling of the termini, involve multiple reaction and purification steps, and may result in loss of the attached functional group by proteolysis.^18^ Another common strategy for selective addition of multiple functional groups is to introduce reactive unnatural amino acids into the protein sequence,^19–21^ which can be selectively modified in the presence of other reactive groups and this approach has been successfully applied to DARPins.^21^ However, unnatural amino acid strategies require expensive unnatural amino acids and more complex expression protocols.

In 2002, Ratner *et al.* ^22^ proposed that if a protein contains two free cysteines that have a sufficient difference in reactivity, then the most reactive cysteine can first be selectively reacted to completion by addition of a weakly thiol reactive reagent and the remaining, less reactive cysteine, subsequently modified with a highly thiol reactive reagent to generate a dual modified protein. Although this is a powerful and useful concept, it has brought limited success to date due to the difficulty in identifying cysteines with suitable differences in reactivity – leading to heterogeneous products and the requirement of intermediate purification steps. Herein, for the first time, we deliver a realisation of the strategy with complete selectivity, without the need for intermediate purification, on a protein of huge significance and relevance. Through identification of cysteine environments with significant differences in nucleophilicity, simple, rapid and site-selective dual modification of DARPins has been enabled.

The model DAPRin protein that we selected for analysis is a HER2-binding DARPin (“HER2DARPin”, PDBID: 2JAB),^23,24^ which contains two internal repeats. HER2 is a validated target for the treatment of breast cancer, as demonstrated by the success of the monoclonal antibody trastuzumab,^25^ and the chemically functionalised variant trastuzumab-emtansine.^26^ We believe that if DARPins could be selectively functionalised in a facile manner, a number of useful applications would ensue, *e.g.* a novel route to the linking of DARPins with different binding domains to construct bi-, tri- or multi-specific constructs, or functionalisation with a PEG chain and a cytotoxic drug to construct a targeted therapeutic.^27,28^ In this study, we were interested in providing a proof of concept for the site-selective dual modification of DARPins.

We sought to identify two residues for mutation to cysteine that gave vastly different cysteine reactivity. From the first internal repeat, all residues with a solvent-accessible surface area greater than 20 Å^2^ (calculated using DSSP)^29^ and distal to the binding site were selected for mutation to cysteine (see Table 1). Due to DARPins comprising conserved repeat units, it was assumed that analogous mutations on adjacent repeats would yield similar results, thus mutations on adjacent repeats were not initially tested. By reaction with *N*-methylmaleimide, each mutant was confirmed to have a single free cysteine (see ESI,† Fig. S13–S39).

**Table 1:** Reactivity of single-cysteine mutants with BrAcEGMe (5 eq.) in PBS pH 7.4 for 1 h at 4 °C, and a single DARPin repeat highlighting the side-chains of residues mutated

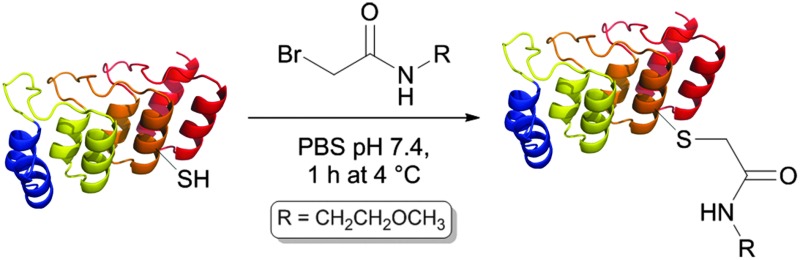		
	Mutation	Reaction with BrAcEGMe^*a*^ (%)
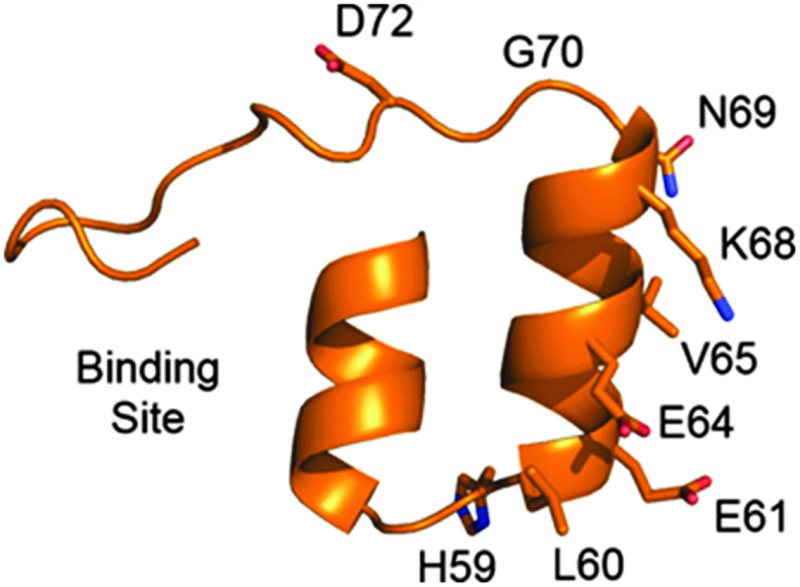	D72C	100
	G70C	18
	N69C	0
	K68C	16
	V65C	5
	E64C	17
	E61C	57
	L60C	4
	H59C	29

^*a*^Determined by relative peak areas in mass spectrometry.

In order to test the reactivity of the single-cysteine DARPin mutants, reduced mutants were reacted with a weakly thiol-reactive reagent under mild conditions, namely 1 mM 2-bromo-*N*-(2-methoxyethyl)acetamide (BrAcEGMe, 5 eq.) in PBS pH 7.4 for 1 h at 4 °C. After this, the progress of the reaction was evaluated by mass spectrometry (Table 1).

Although we observed a wide range of reactivity across the series of mutants evaluated, we were most pleased to observe a mutant that reacted completely, D72C, and one that gave no reaction (within experimental error), N69C, as this paved the way to appraise the site-selective dual modification strategy discussed. We next sought to determine whether equivalent mutations on adjacent repeats would display similar reactivity (see Fig. 1). We thus generated mutations equivalent to D72C (*i.e.* D39C and D105C). To our delight, these mutants were also found to be highly reactive towards BrAcEGMe (see ESI,† Fig S10–S12 and S43–S45). Mutations that were equivalent to N69C (*i.e.* N36C and H102C) were next generated. Gratifyingly, these mutants were found to be highly unreactive (see ESI,† Fig. S4–S6 and S40–S42). The L135C mutation, which may have been predicted to be unreactive, showed *ca.* 25% reactivity towards BrAcEGMe (see ESI,† Fig. S46–S48). This is not surprising, however, as the L135C position is the penultimate residue on the protein and is therefore likely to experience a different micro-environment to N69C, N36C and H102C, due to increased flexibility at the C-terminus.^30^ Recently, improved sequences for the N-terminal^31^ and C-terminal^30^ capping helices have been described. We found that the poor reactivity of the N36C mutation was retained when combined with the improved N-terminal sequence (ESI,† Fig. S7–S9). Gratifyingly, the increased stability conferred by the improved C-terminal mutations significantly lowered the reactivity of the L135C mutant (ESI,† Fig. S49–S51).

**Fig. 1:**
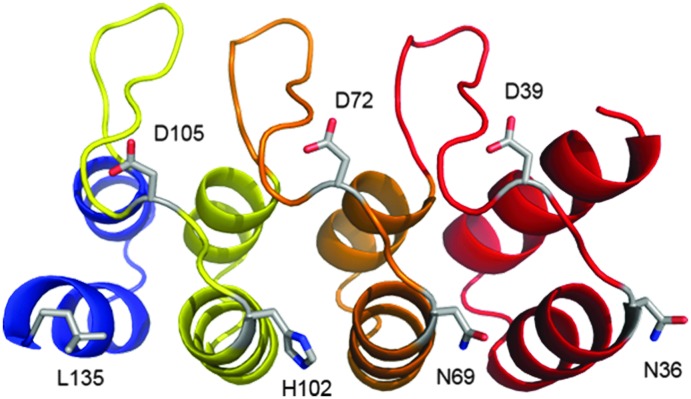
Location of residues analogous to N69 and D72.

We next sought to generate DARPin dual cysteine mutants in which the cysteines could be reacted sequentially. Using the same HER2DARPin, we generated an N69C, D72C dual mutant in which the cysteine residues are proximal, and an N36C, D105C dual mutant in which the cysteine residues are distal. In order to show the generality of our technique, we also generated an N69C, D72C mutant of the small DARPin protein mut4,^32^ which has a single internal repeat (11 kDa). In each case, we successfully demonstrated selective addition of BrAcEGMe to one of the two cysteine residues (see ESI,† Fig. S52–S60). In order to demonstrate that a second functional reagent could be added to the protein in a controlled manner, we took the HER2DARPin(N36C, D105CAcEGMe) that was functionalised with ethylene glycol methyl ether, and added tetramethylrhodamine-5-(and -6-) C2 maleimide (TMRM) to react with the remaining unreacted cysteine. This resulted in a homogenous dual-functionalised DARPin (Fig. 2, ESI,† Fig. S61). Successful dual modification was also demonstrated on HER2DARPin(N69C, D72C) and mut4(N69C, D72C) (see ESI,† Fig. S62–S63).

**Fig. 2:**
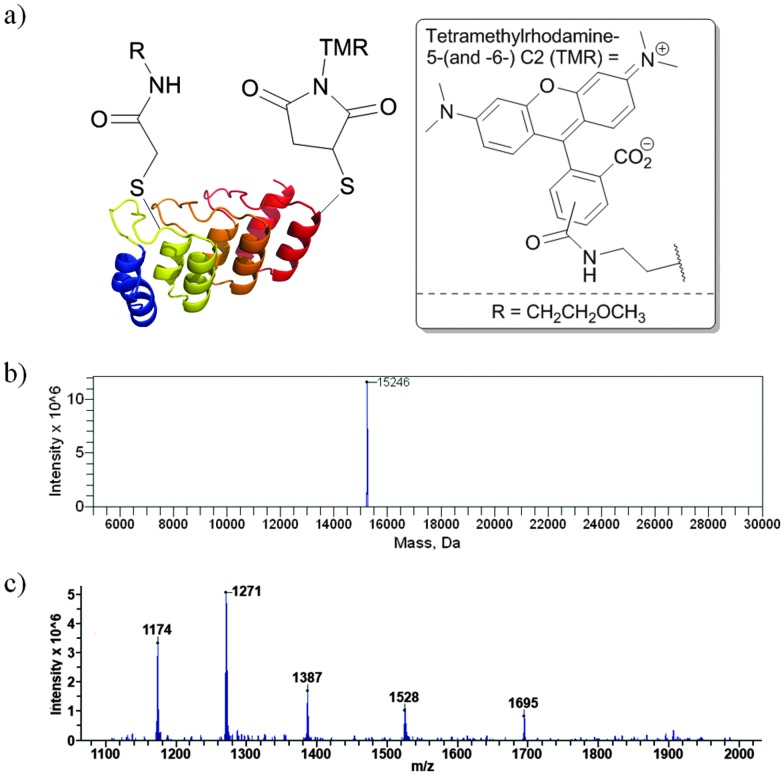
(a) HER2DARPin(N36CTMRM, D105CAcEGMe), and (b) deconvoluted and (c) raw mass spectra of HER2DARPin(N36CTMRM, D105CAcEGMe). Expected mass: 15 241 Da, observed mass: 15 246 Da.

Given the use of DARPins as binding proteins, it was important to evaluate whether the structure and/or stability had been affected by introduction and modification of two cysteine residues. Pleasingly, circular dichroism spectra (Fig. 3a) were indistinguishable for wild-type HER2DARPin, HER2DARPin(N36C, D105C) reacted with NMM, and dual-functionalised HER2DARPin(N36CTMRM, D105CAcEGMe). This implies that the structure is unaffected by introduction and/or modification of the two cysteine residues. Moreover, thermal unfolding data (Fig. 3b) showed an unfolding transition at slightly higher temperatures for the dual cysteine mutants, indicating that, if anything, the N36C, D105C mutations may increase the stability of HER2DARPin.

**Fig. 3:**
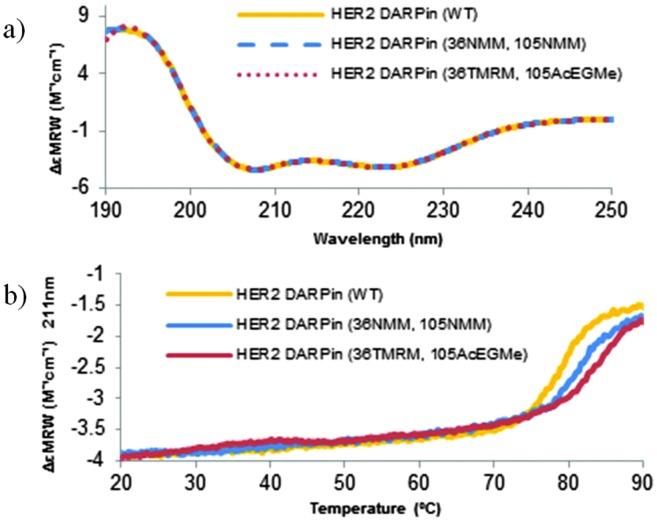
(a) Circular dichroism spectra of HER2DARPin variants, and (b) thermal unfolding of HER2DARPin variants.

In this communication, we have identified cysteine mutations that can be sequentially modified to enable rapid and site-selective dual modification of DARPins. We have shown that the reactivity of the cysteine mutations is preserved when the mutations are applied both to analogous positions within the same protein, and to analogous positions on another DARPin. We hence expect this strategy to be applicable across the class of DARPins. To the best of our knowledge, no other strategy allows site-selective, dual modification of DARPins in such a simple, rapid and cost-effective manner. Considering the academic, diagnostic and therapeutic potential of DARPins, we anticipate a number of applications to follow from our discovery.

We gratefully acknowledge the Wellcome Trust, MRC, RCUK, EPSRC and UCLB for support of our programme, and Justin Molloy for helpful discussions.
