# Supplementary material for: A rapid, site-selective and efficient route to the dual modification of DARPins
Source: Chem Commun (Camb). 2014 Apr 1;50(38):4898–900. doi: 10.1039/c4cc00053f (PMC4091302; doi:10.1039/c4cc00053f)
Supplement: Supplementary file 1 [file CC-050-C4CC00053F-s001.pdf]

Electronic Supplementary Material (ESI) for ChemComm.
This journal is © The Royal Society of Chemistry 2014

A rapid, site-selective and efficient route to the dual modification of DARPins

Paul Moody,[a,b] Vijay Chudasama,[a] Ramiz I. Nathani,[a] Antoine Maruani,[a] Stephen Martin,[b] Justin Molloy,[b] Mark E. B. Smith[a] and Stephen Caddick*[a]

[a] *Department of Chemistry, University College London, 20 Gordon Street, London, WC1H OAJ, UK;* [b] *MRC National Institute for Medical Research, The Ridgeway, Mill Hill, London, NW7 1AA, UK.*

Tel: +44 (0)20 3108 5071; Fax: +44 (0)20 7679 7463; E-mail: vpenterprise@ucl.ac.uk

Mutation and expression of DARPin proteins

HERDARPin(WT), HER2DARPin(Cmut, L135C) and Mut4DARPin(N69C, D72C) DARPin genes were purchased from Genscript in the vector pUC57, flanked by the upstream and downstream T7 control sequences used in pET21. Sequences of purchased genes are provided in Supplementary Data 1. The primers used are provided in Supplementary Table 1, and all mutations were confirmed by DNA sequencing.

DARPins were expressed directly from pUC57 plasmids in LysY/lacIq cells (purchased from NEB). Cells were lysed by sonication, and proteins were purified by nickel affinity chromatography in the presence of 14 mM 2-mercaptoethanol. Proteins were buffer exchanged into PBS with 1 mM EDTA by repeated ultrafiltration, and aliquots were stored at -80 °C.

Solvent accessibility calculations

Solvent accessibilities of sidechains in HER2DARPin were calculated by DSSP, using the default parameters. Calculations were performed on the A chain of PDB file 2JAB.

Reactions with 2-Bromo-*N*-(2-methoxy-ethyl)-acetamide (BrAcEGMe)

2-Bromo-*N*-(2-methoxy-ethyl)-acetamide (BrAcEGMe) (Mw 196 Da) was purchased from Vitas M Labs. A stock solution of 10 mM BrAcEGMe was prepared in DMF, and stored at -80 °C. Proteins were reduced by incubation with 1 mM DTT in phosphate buffered saline (PBS) pH 7.4 with 1 mM EDTA for 1 h on 4 °C, followed by removal of excess DTT by ultrafiltration. 3 mg/mL of reduced protein (200 µM) was reacted with 1 mM BrAcEGMe in PBS (pH 7.4), with 10% DMF and 1 mM EDTA. Reactions

were performed with a 50 µL total volume in 250 µL PCR tubes on 4 °C for 1 h and analysed by LC-MS.

Reactions with *N*-methyl maleimide (NMM)

N-Methyl maleimide (NMM) (Mw 111.1 Da) was purchased from Sigma Aldrich. A stock solution of 10 mM NMM was prepared in DMF, and stored at -80 °C. Proteins were reduced by incubation with 1 mM DTT in PBS (pH 7.4) with 1 mM EDTA for 1 h on 4 °C, followed by removal of excess DTT by ultrafiltration. 3 mg/mL of reduced protein (200 µM) was reacted with 1 mM NMM in PBS buffer pH 7.4, with 10% DMF and 1 mM EDTA. Reactions were performed with a 50 µL total volume in 250 µL PCR tubes on 4 °C for 1 h and analysed by LC-MS.

Reaction with Tetramethylrhodamine-5-(and-6) C2 maleimide (TMRM)

Tetramethylrhodamine-5-(and -6-) C2 maleimide (TMRM) (Mw 552.6 Da) was purchased from Anaspec. A stock solution of 10 mM TMRM was prepared in DMF, and stored at -80 °C. Proteins were reduced by incubation with 1 mM DTT in PBS (pH 7.4) with 1 mM EDTA for 1 h on 4 °C, followed by removal of excess DTT by ultrafiltration. 3 mg/mL protein (200 µM) was reacted with 1 mM TMRM in PBS buffer pH 7.4, with 10% DMF and 1 mM EDTA. Reactions were performed with a 50 µL total volume in 250 µL PCR tubes on 4 °C for 1 h and the reaction quenched by addition of 10 mM 2-mercaptoethanol. Excess TMRM was removed by ultrafiltration, followed by size-exclusion chromatography, using a HiPrep 26/60 sephacryl S-100 column in PBS (pH 7.4). The product was analysed by LC-MS.

Protein Mass Spectrometry

LC-MS was performed on protein samples using a Thermo Scientific uPLC connected to MSQ Plus Single Quad Detector (SQD). Column: Hypersil Gold C4, 1.9 µm, 2.1 x 50 mm. Wavelength: 254 nm. Mobile Phase: 99:1 Water (0.1% formic acid): MeCN (0.1% formic acid) to 1:9 Water (0.1% formic acid):MeCN (0.1% formic acid) gradient over 4 min. Flow Rate: 0.3 mL/min. MS Mode: ES+. Scan Range: m/z = 500-2000. Scan time: 1.5 s. Data obtained in continuum mode. The electrospray source of the MS was operated with a capillary voltage of 3.5 kV and a cone voltage of 50 V. Nitrogen was used as the nebulizer and desolvation gas at a total flow of 600 L/h. Ion series were generated by integration of the total ion chromatogram (TIC) over the major TIC peak. Total mass spectra for protein samples were reconstructed from the ion series using the pre-installed ProMass software using default settings for large proteins in m/z range 1100-2000. The m/z range 500-1100 was not used for deconvolution as cation adducts were observed in this range.

Circular Dichroism and Thermal Unfolding

HER2 DARPin (36TMRM, 105BrAcEGMe) was generated by reaction with BrAcEGMe followed by reaction with TMRM, as described above. HER2DARPin(WT) and HER2DARPin(36NMM, 105NMM) were generated by reaction of HER2DARPin(WT) and DARPin(N36C, D105C) respectively with NMM, as described above. Excess NMM was removed by repeated ultrafiltration into PBS.

Circular dichroism (CD) spectra were recorded in 1 mm fused silica cuvettes using a Jasco J-715 spectrophotometer at room temperature in PBS, with 0.15 mg/ml of each construct. Thermal unfolding curves were obtained by monitoring the CD signal at 211 nm using 2-mm pathlength cuvettes and a heating rate of 1°/min. Spectra were normalised using a constant scaling factor.

Supplementary Data 1. Sequences of purchased genes

HER2DARPin(WT)
TAATACGACTCACTATAGGGGAATTGTGAGCGGATAACAATTCCCCTCTAGAAATAATTTTG
TTTAACTTTAAGAAGGAGATATACAT
ATGCGCGGTAGCCACCATCACCACCACCACGGTAGCGACCTGGGCAAAAAACTGCTGGAAGC
GGCACGTGCGGGCCAAGACGACGAAGTGCGTATTCTGATGGCGAACGGCGCCGATGTTAATG
CGAAAGACGAATATGGTCTGACCCCGCTGTACCTGGCAACGGCTCATGGCCACCTGGAAATT
GTGGAAGTTCTGCTGAAAAACGGCGCCGATGTCAATGCAGTGGACGCTATCGGTTTTACCCC
GCTGCATCTGGCGGCCTTCATTGGTCACCTGGAAATCGCAGAAGTTCTGCTGAAACATGGCG
CGGATGTCAACGCCCAGGACAAATTTGGTAAAACGGCTTTCGACATCTCCATTGGTAACGGC
AACGAAGACCTGGCTGAAATCCTGCAGAAACTGAACTGA
TGCTGCCACCGCTGAGCAATAACTAGCATAACCCCTTGGGGCCTCTAAACGGGTCTTGAGGG
GTTTTTTG

HER2DARPin(Cmut, L135C)
TAATACGACTCACTATAGGGGAATTGTGAGCGGATAACAATTCCCCTCTAGAAATAATTTTG
TTTAACTTTAAGAAGGAGATATACAT
ATGCGCGGTAGCCACCACCATCATCATCACGGTAGCGACCTGGGCAAAAAACTGCTGGAAGC
GGCACGTGCGGGCCAAGACGACGAAGTGCGTATTCTGATGGCGAACGGCGCCGATGTTAATG
CGAAAGACGAATATGGTCTGACCCCGCTGTACCTGGCAACGGCTCATGGCCACCTGGAAATT
GTGGAAGTTCTGCTGAAAAACGGCGCAGATGTCAATGCAGTGGACGCTATCGGTTTTACCCC
GCTGCATCTGGCGGCCTTCATTGGTCACCTGGAAATCGCGGAAGTTCTGCTGAAACATGGCG
CGGATGTCAACGCCCAGGACAAATTTGGTAAAACGCCGTTCGATCTGGCTATTCGTGAAGGT
CATGAAGACATCGCTGAAGTCCTGCAGAAATGTGCGTAA
TGCTGCCACCGCTGAGCAATAACTAGCATAACCCCTTGGGGCCTCTAAACGGGTCTTGAGGG
GTTTTTTG

Mut4DARPin(N69C, D72C)
TAATACGACTCACTATAGGGGAATTGTGAGCGGATAACAATTCCCCTCTAGAAATAATTTTG
TTTAACTTTAAGAAGGAGATATACAT
ATGCGCGGTAGTCATCATCATCATCATCATGGCTCGGATCTGGGCAAGAAACTGCTGGAAGC
GGCCCGTGCTGGTCAGGACGACGAAGTCCGTATTCTGATGGCAAACGGCGCTGATGTGAATG
CAAAAGATAAGGACGGTTATACCCCGCTGCATCTGGCAGCACGTGAAGGTCACCTGGAAATC
GTGGAAGTTCTGCTGAAATGCGGCGCGTGTGTTAACGCCCAGGACAAATTTGGCAAGACGGC
CTTCGACATCTCTATCGACAATGGTAATGAAGACCTGGCTGAAATCCTGCAAAAGGCTGCGT
AA
TGCTGCCACCGCTGAGCAATAACTAGCATAACCCCTTGGGGCCTCTAAACGGGTCTTGAGGG
GTTTTTTG

Supplementary Table 1. Primers used for mutagenesis

Mutation	Forwards primer	Reverse primer
N36C	GGCGTGCGGGGCCGATGTTAAT GCGAAAGAC	CATCGGCCCCGCACGCCATCAG AATACGC
Nmut, N36C	CGTGAACTGCTGAAATGCGGCG CCGATGTTAATGCGAAAGACG	GCATTTCAGCAGTTCACGCACTT CGTCGTCTTGGCCCGCACG
D39C	GGCGAACGGGGCCTGTGTTAAT GCGAAAGAC	GCGTATTCTGATGGCGAACGGG GCCTGTG
H59C	CTCATGGCTGTCTGGAAATTGTG GAAGTTCTGC	CAATTTCCAGACAGCCATGAGC CGTTGCC
L60C	CTGCGAAATTGTGGAAGTTCTGC TGAAAAACGGCG	CAGAACTTCCACAATTTCGCAGT GGCCATGAGC
E61C	CCTGTGTATTGTGGAAGTTCTGC TGAAAAACGGCG	CAGAACTTCCACAATACACAGGT GGCCATGAGC
E64C	CCTGGAAATTGTGTGTGTTCTGC TGAAAAACGGCG	CAGAACACACACAATTTCCAGGT GGCCATGAGC
V65C	CCTGGAAATTGTGGAATGTCTGC TGAAAAACGGCG	CAGACATTCCACAATTTCCAGGT GGCCATGAGC
K68C	GTGTAACGGCGCCGATGTCAAT GCAGTGGACG	GACATCGGCGCCGTTACACAGC AGAACTTCC
N69C	GAAATGTGGCGCCGATGTCAAT GCAGTGGACG	GACATCGGCGCCACATTTCAGC AGAACTTCC
G70C	GAAAAACTGTGCCGATGTCAATG CAGTGGACG	GACATCGGCACAGTTTTTCAGCA GAACTTCC
D72C	GAAAAACGGCGCCTGTGTCAAT GCAGTGGACG	GACACAGGCGCCGTTTTTCAGC AGAACTTCC
H102C	CTGAAATGTGGAGCGGATGTCA ACGCCCAGGAC	GACATCCGCTCCACATTTCAGCA GAACTTCT
D105C	CTGAAACATGGAGCGTGTGTCA ACGCCCAGGAC	GACACACGCTCCATGTTTCAGCA GAACTTCT
L135C	TGTAACTGATGCTGCCACCGCT GAGCAATAACTAGC	GTGGCAGCATCAGTTACATTTCT GCAGGATTTCAGCCAG

Supplementary Figure 1. HER2DARPin(WT) in PBS, 1 mM EDTA.
Expected mass 14596.4 Da

Sequence
MRGSHHHHHHGSDLGKKLLEAARAGQDDEVRILMANGADVNAKDEYGLTPLYLATAHGHLEI
VEVLLKNGADVNAVDAIGFTPLHLAAFIGHLEIAEVLLKHGADVNAQDKFGKTAFDISIGNG
NEDLAEILQKLN

a)

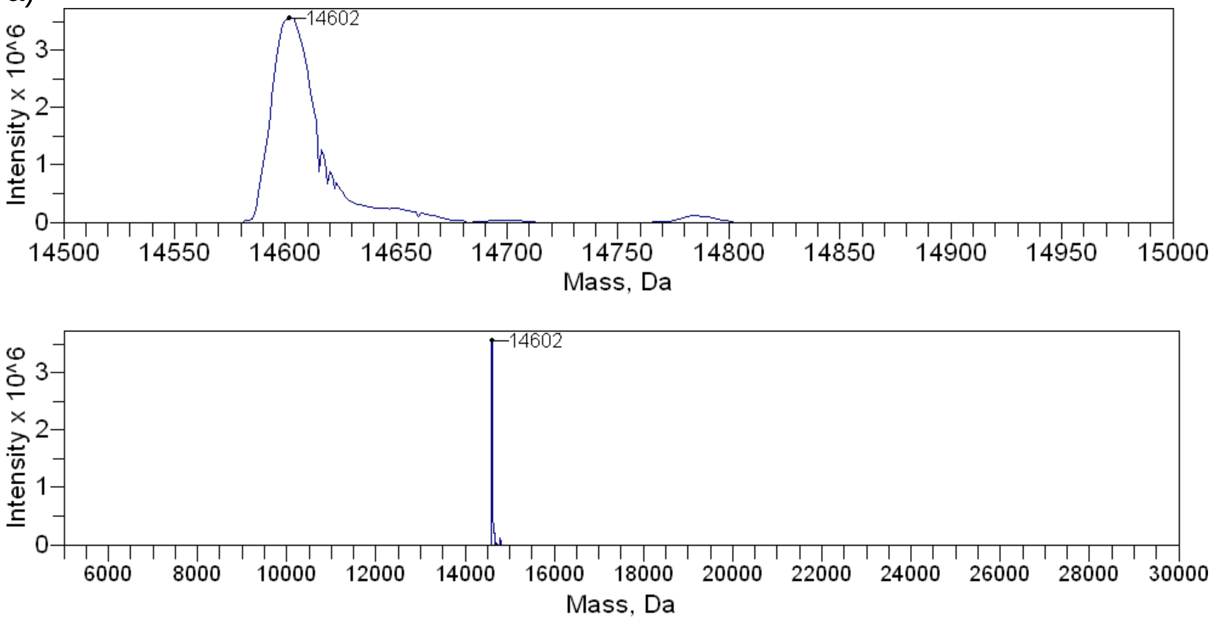

b)

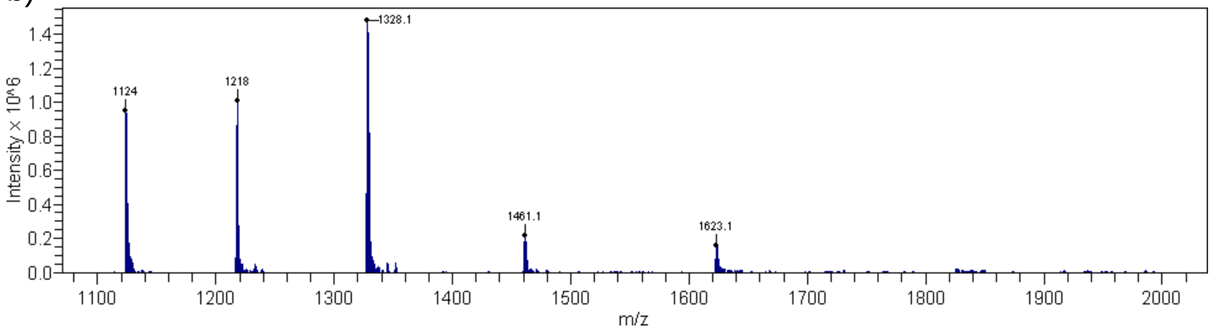

c)

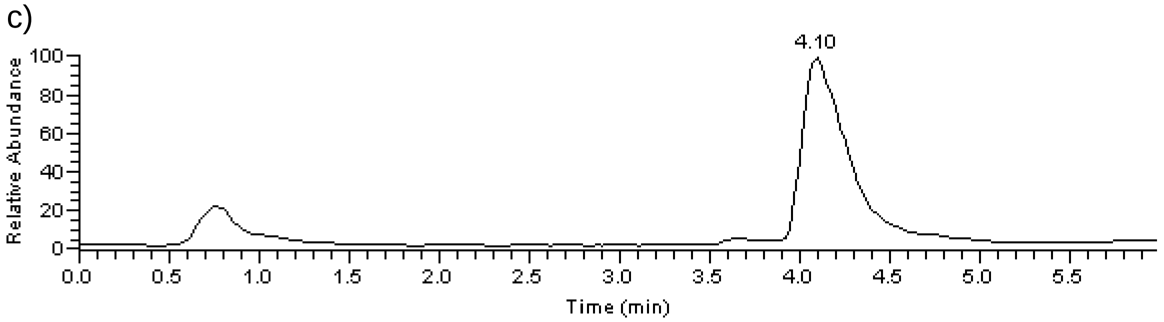

(a) deconvoluted, (b) non-deconvoluted, and (c) TIC mass spectrometry data for HER2DARPin(WT).

Supplementary Figure 2. HER2DARPin(WT) with 1 mM BrAcEGMe, 1 mM EDTA, in PBS pH 7.4, 10% DMF, 2 h, at 4 °C.
Expected mass (unmodified) 14596.4 Da
Expected mass (modified) 14711.6 Da

a)

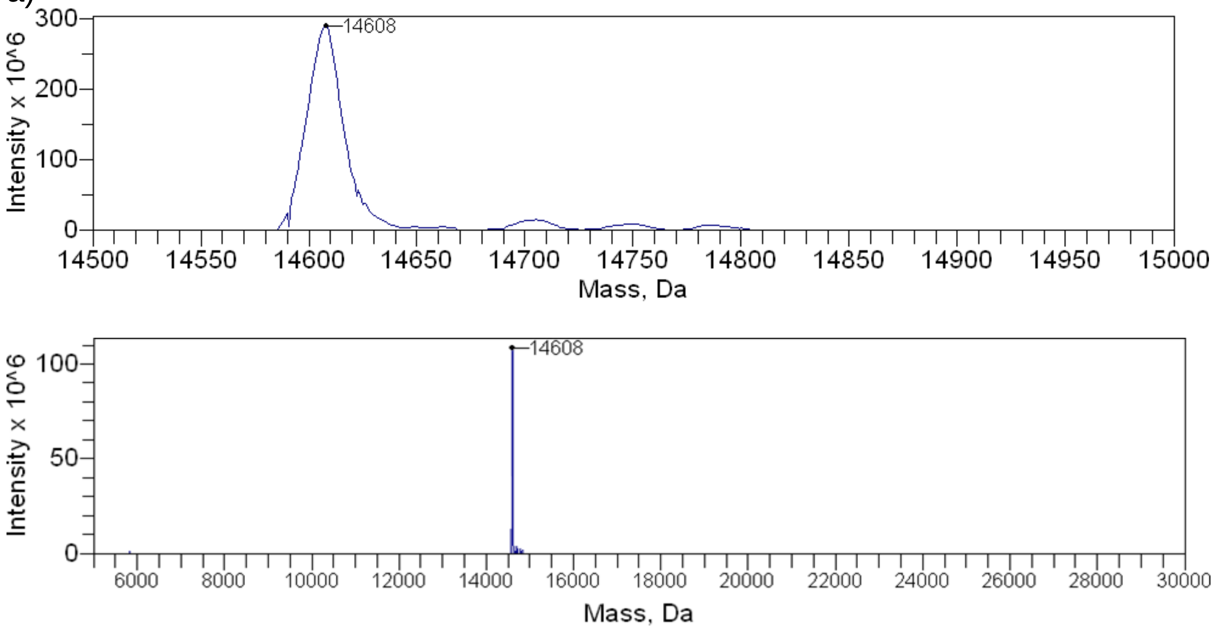

b)

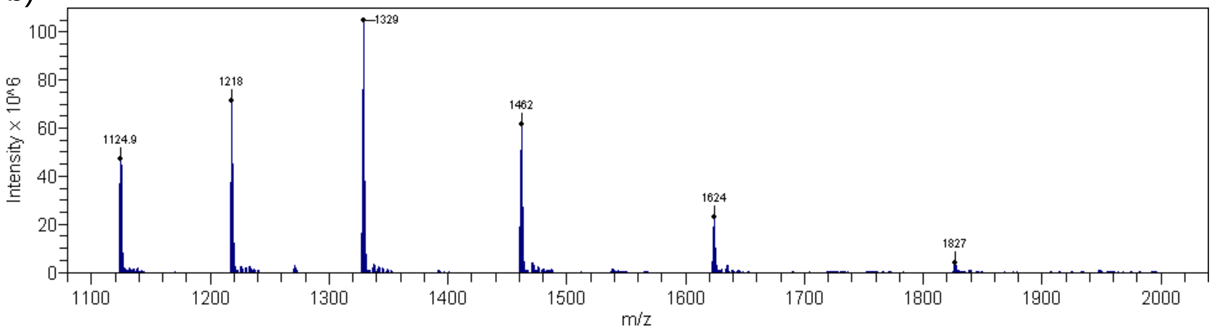

c)

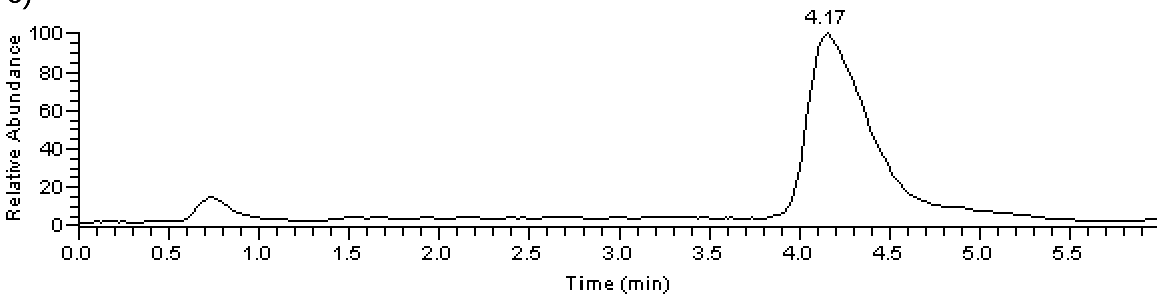

(a) deconvoluted, (b) non-deconvoluted, and (c) TIC mass spectrometry data for HER2DARPin(WT) reacted with BrAcEGMe.

Supplementary Figure 3. HER2DARPin(WT) with 1 mM NMM, 1 mM EDTA, in PBS pH 7.4, 10% DMF, 1 h, at 4 °C.
Expected mass (unmodified) 14596.4 Da
Expected mass (modified) 14707.5 Da

a)

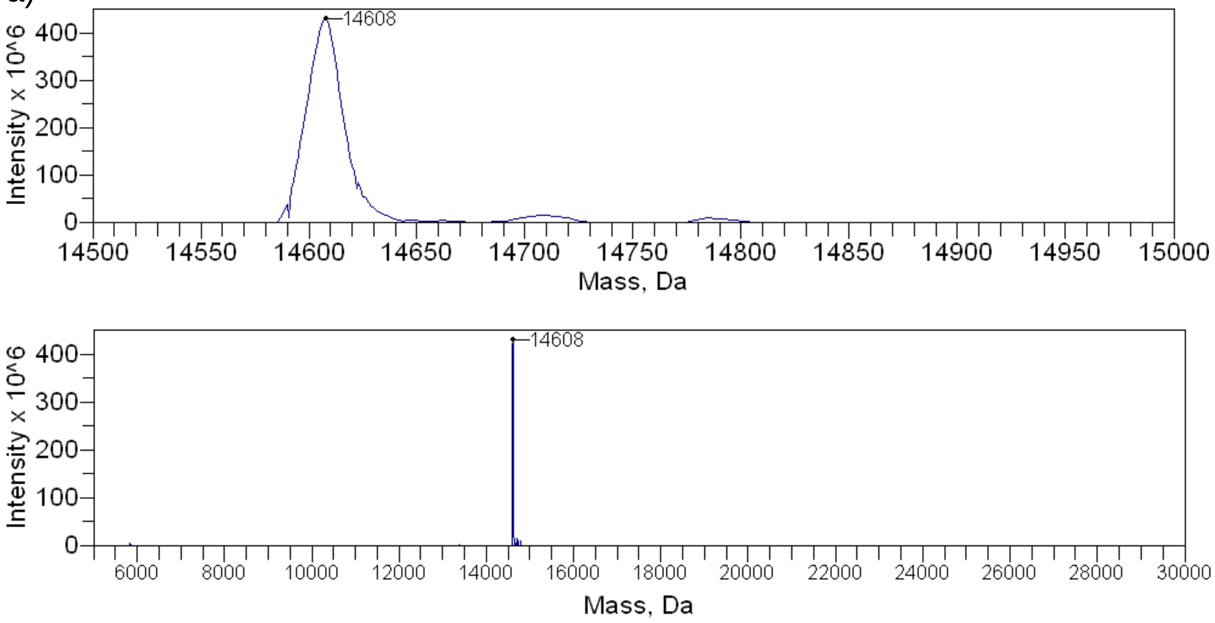

b)

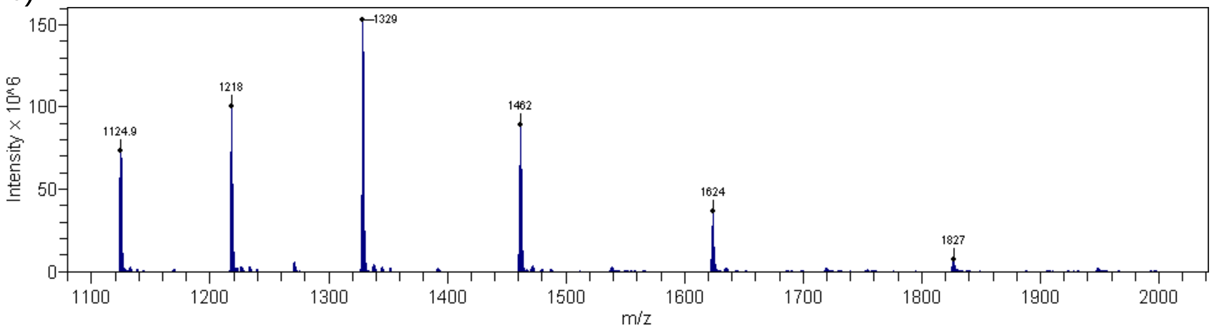

c)

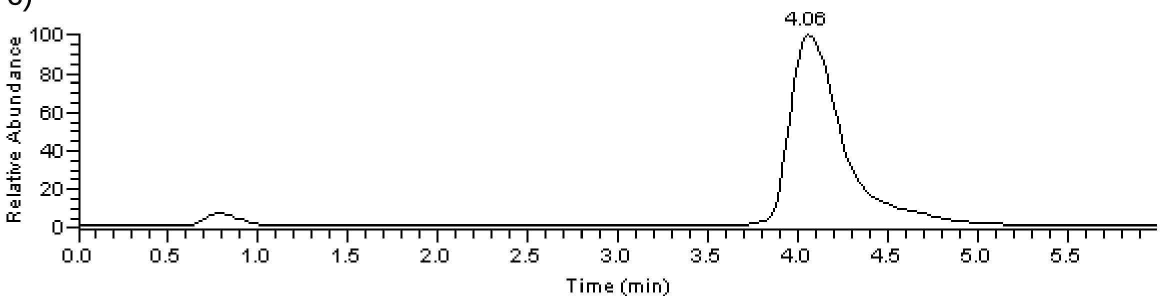

(a) deconvoluted, (b) non-deconvoluted, and (c) TIC mass spectrometry data for HER2DARPin(WT) reacted with NMM.

Supplementary Figure 4. HER2DARPin(N36C) in PBS, 1 mM EDTA. Expected mass 14585.4 Da

Sequence

MRGSHHHHHHGSDLGKKLLEAARAGQDDEVRILMA**C**GADVNAKDEYGLTPLYLATAHGHLEI
VEVLLKNGADVNAVDAIGFTPLHLAAFIGHLEIAEVLLKHGADVNAQDKFGKTAFDISIGNG
NEDLAEILQKLN

a)

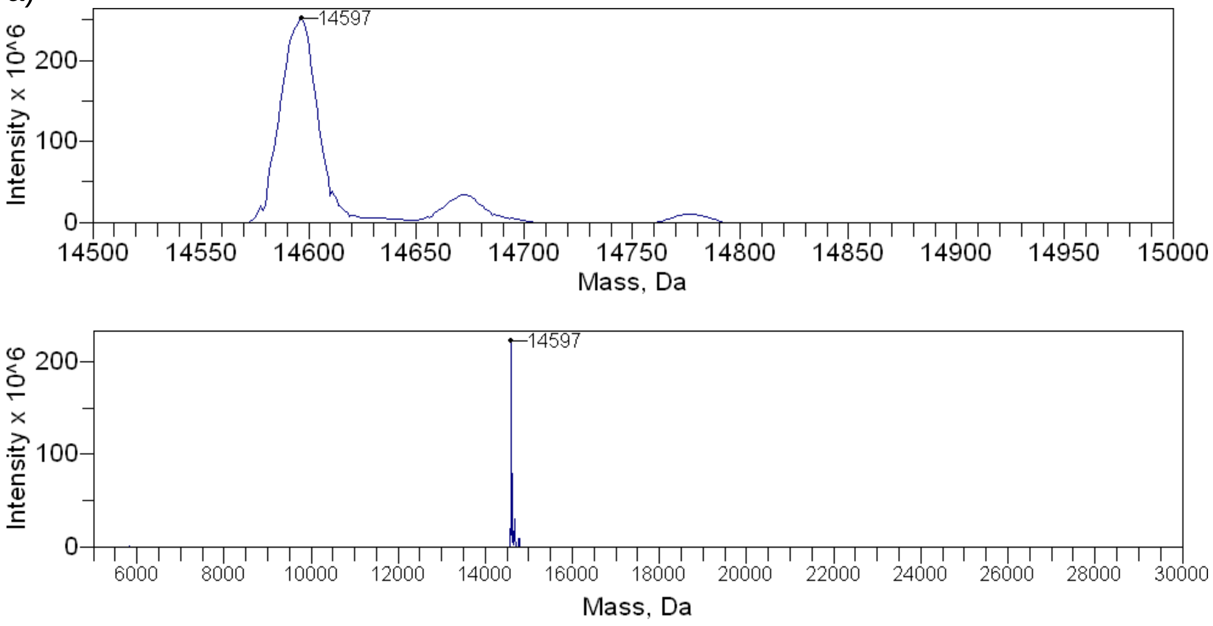

b)

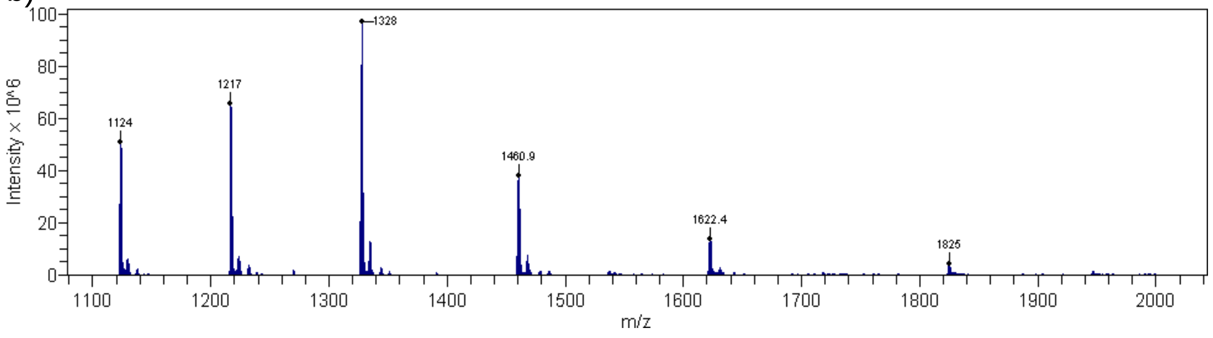

c)

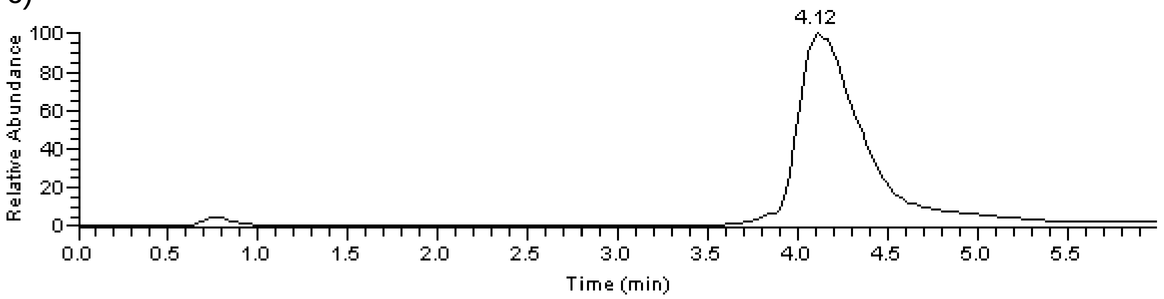

(a) deconvoluted, (b) non-deconvoluted, and (c) TIC mass spectrometry data for HER2DARPin(N36C).

Supplementary Figure 5. HER2DARPin(N36C) with 1 mM BrAcEGMe, 1 mM EDTA, in PBS pH 7.4, 10% DMF, 2 h, at 4 °C.
Expected mass (unmodified) 14585.4 Da
Expected mass (modified) 14700.6 Da

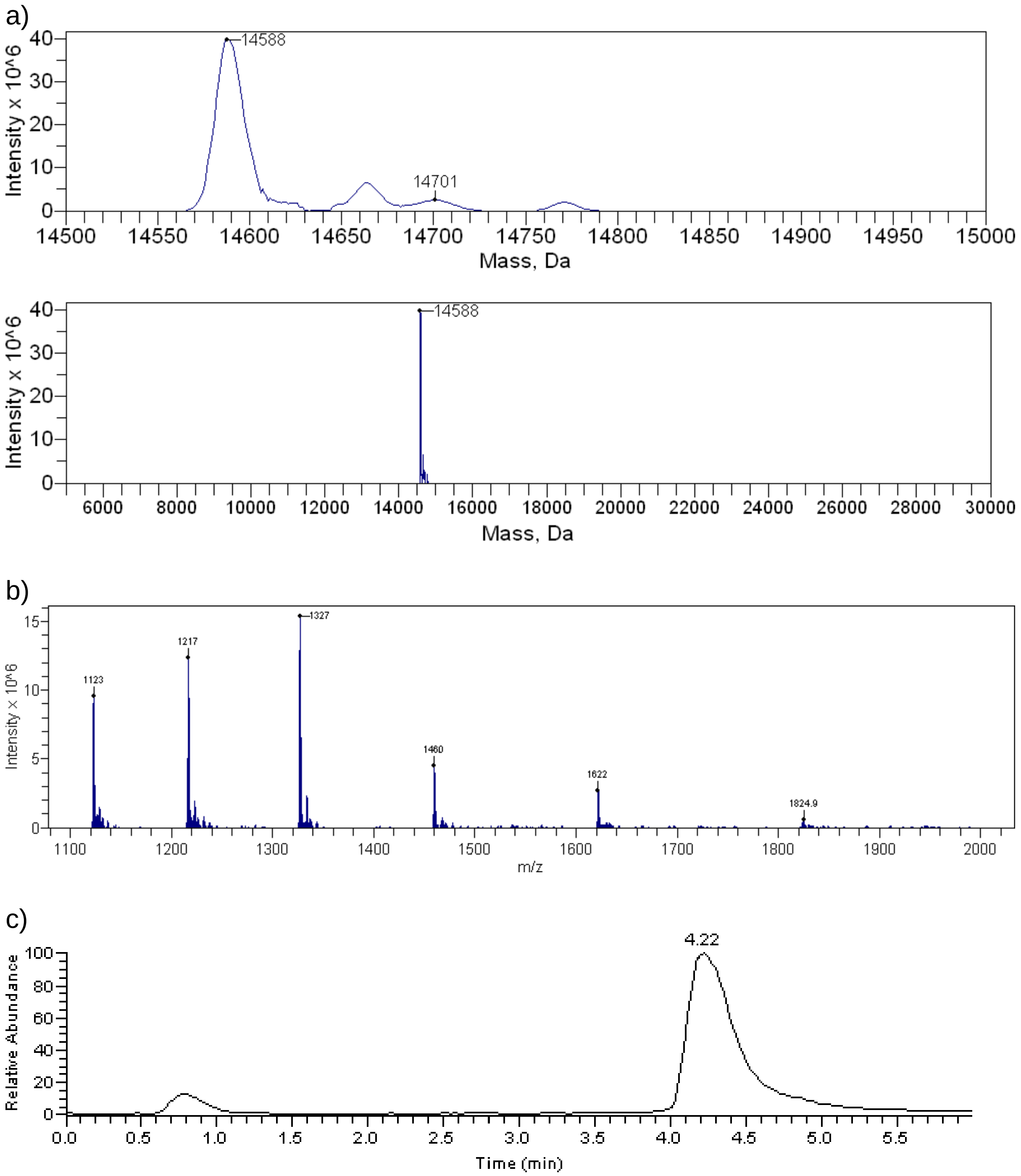

(a) deconvoluted, (b) non-deconvoluted, and (c) TIC mass spectrometry data for HER2DARPin(N36C) reacted with BrAcEGMe.

Supplementary Figure 6. HER2DARPin(N36C) with 1 mM NMM, 1 mM EDTA, in PBS pH 7.4, 10% DMF, 1 h, at 4 °C.
Expected mass (unmodified)   14585.4 Da
Expected mass (modified)   14696.5 Da

a)

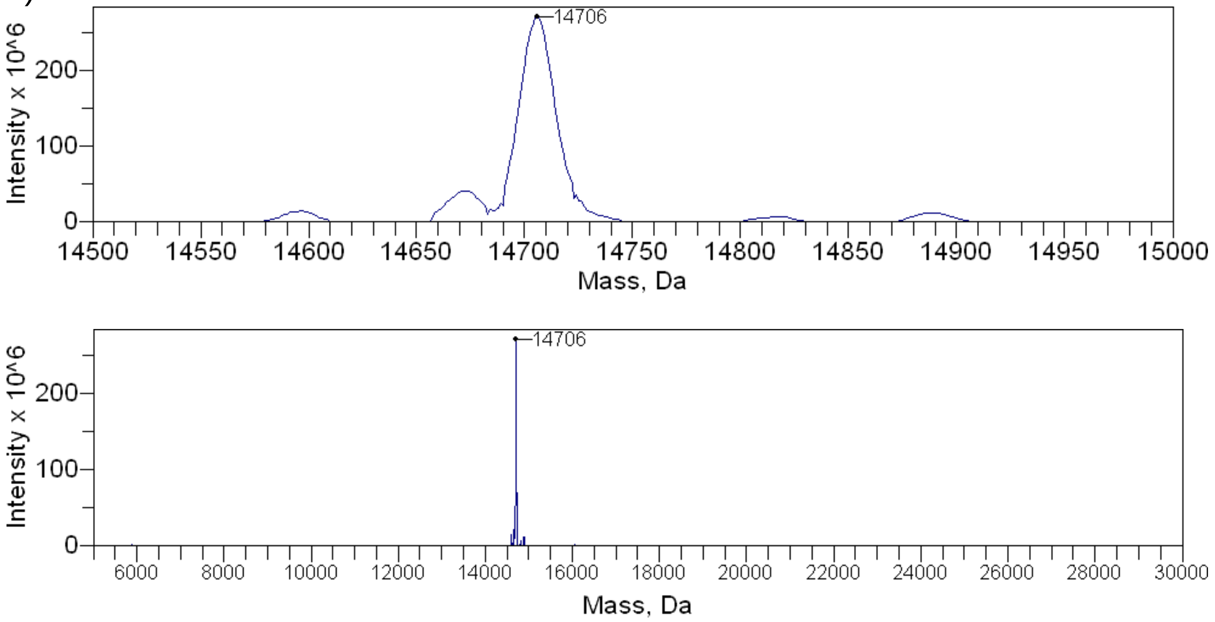

b)

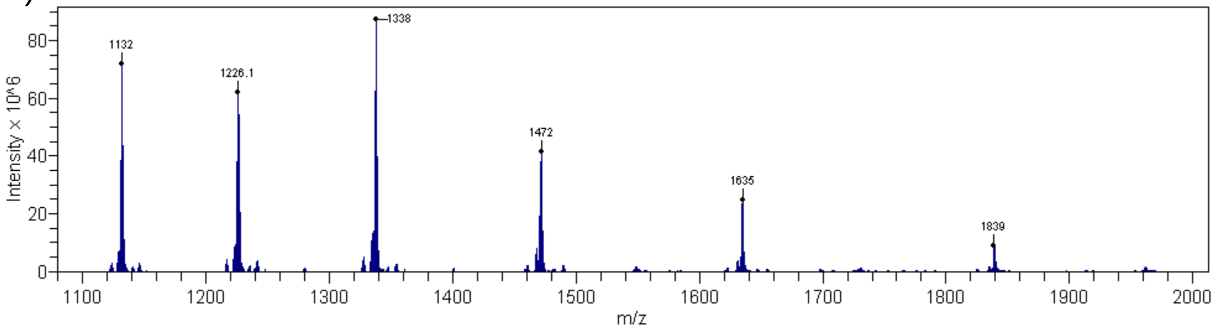

c)

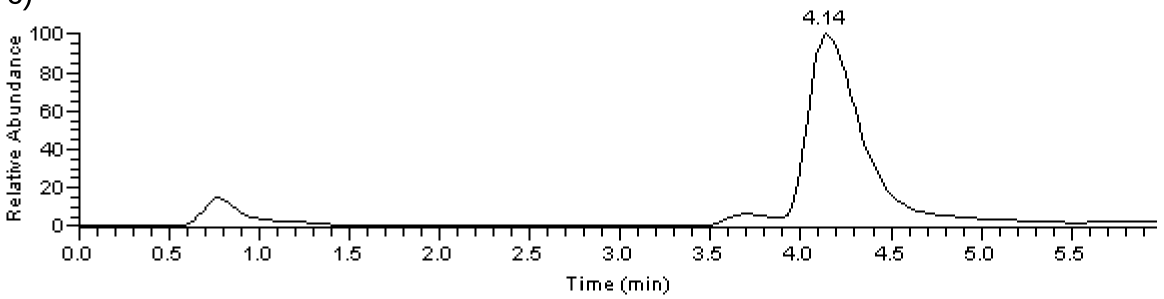

(a) deconvoluted, (b) non-deconvoluted, and (c) TIC mass spectrometry data for HER2DARPin(N36C) reacted with NMM.

Supplementary Figure 7. HER2DARPin(Nmut, N36C) in PBS, 1 mM EDTA. Expected mass 14640.4 Da

Sequence

MRGSHHHHHHGSDLGKKLLEAARAGQDDEVR**ELLKC**GADVNAKDEYGLTPLYLATAHGHLEI
VEVLLKNGADVNAVDAIGFTPLHLAAFIGHLEIAEVLLKHGADVNAQDKFGKTAFDISIGNG
NEDLAEILQKLN

a)

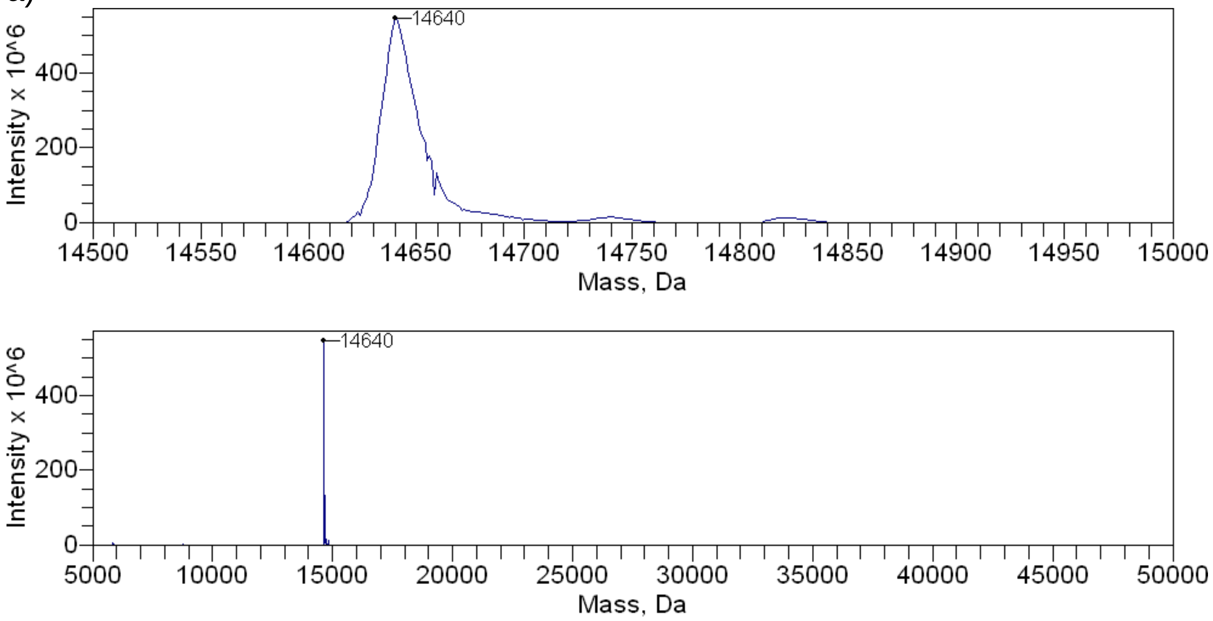

b)

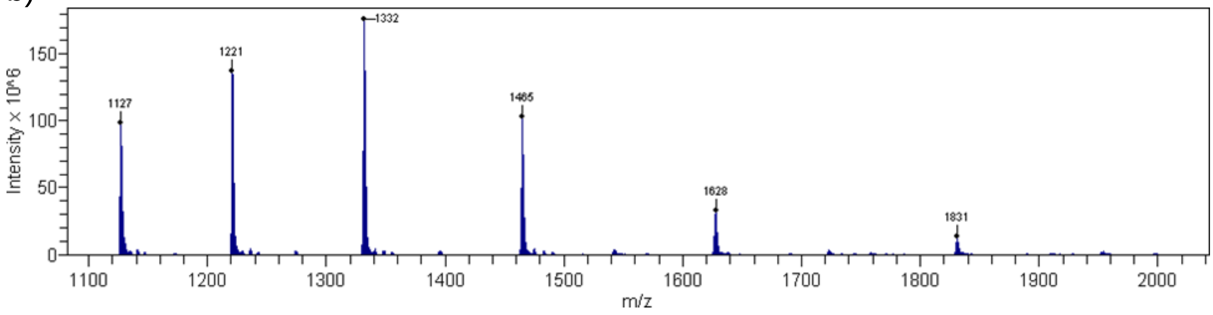

c)

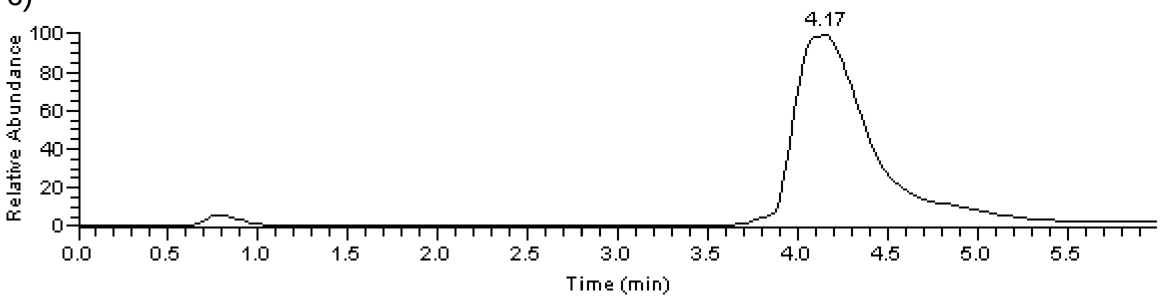

(a) deconvoluted, (b) non-deconvoluted, and (c) TIC mass spectrometry data for HER2DARPin(Nmut, N36C).

Supplementary Figure 8. HER2DARPin(Nmut, N36C) with 1 mM BrAcEGMe, 1 mM EDTA, in PBS pH 7.4, 10% DMF, 2 h, at 4 °C.
Expected mass (unmodified) 14640.4 Da
Expected mass (modified) 14755.6 Da

a)

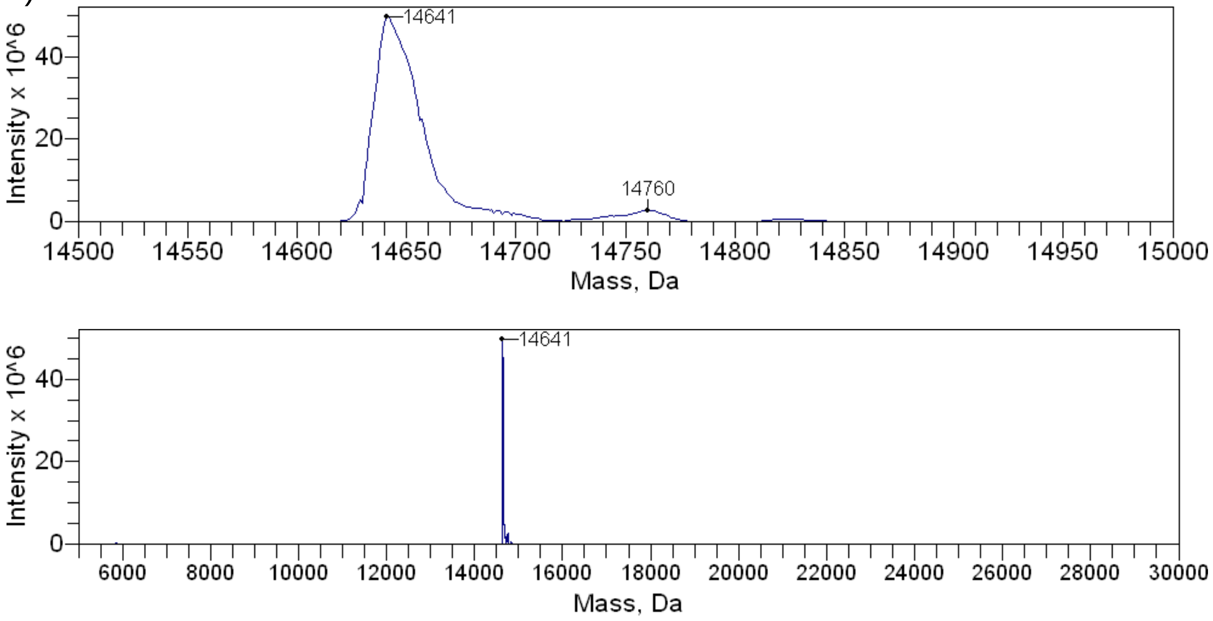

b)

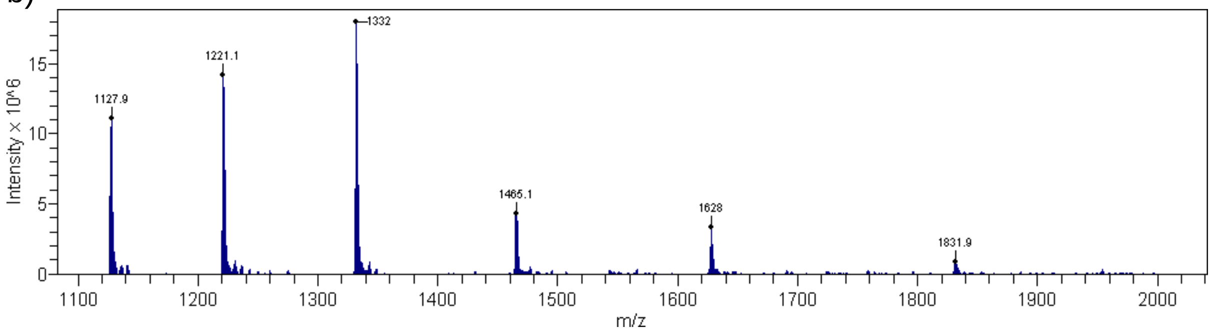

c)

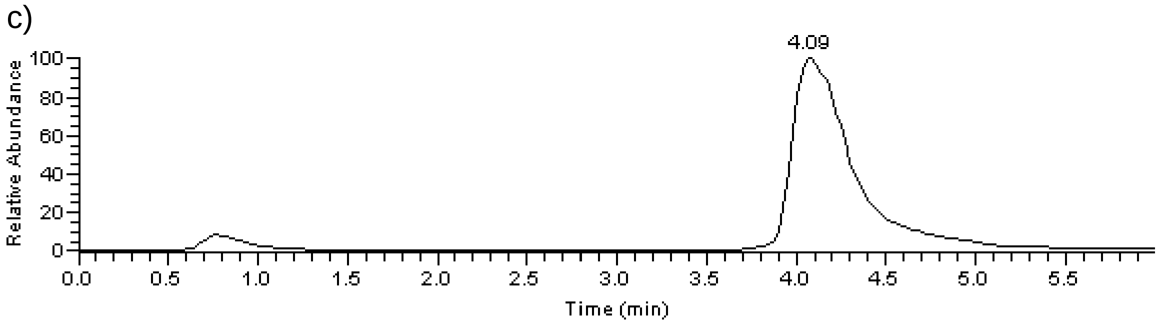

(a) deconvoluted, (b) non-deconvoluted, and (c) TIC mass spectrometry data for HER2DARPin(Nmut, N36C) reacted with BrAcEGMe.

Supplementary Figure 9. HER2DARPin(Nmut, N36C) with 1 mM NMM, 1 mM EDTA, in PBS pH 7.4, 10% DMF, 1 h, at 4 °C.
Expected mass (unmodified) 14640.4 Da
Expected mass (modified) 14751.5 Da

a)

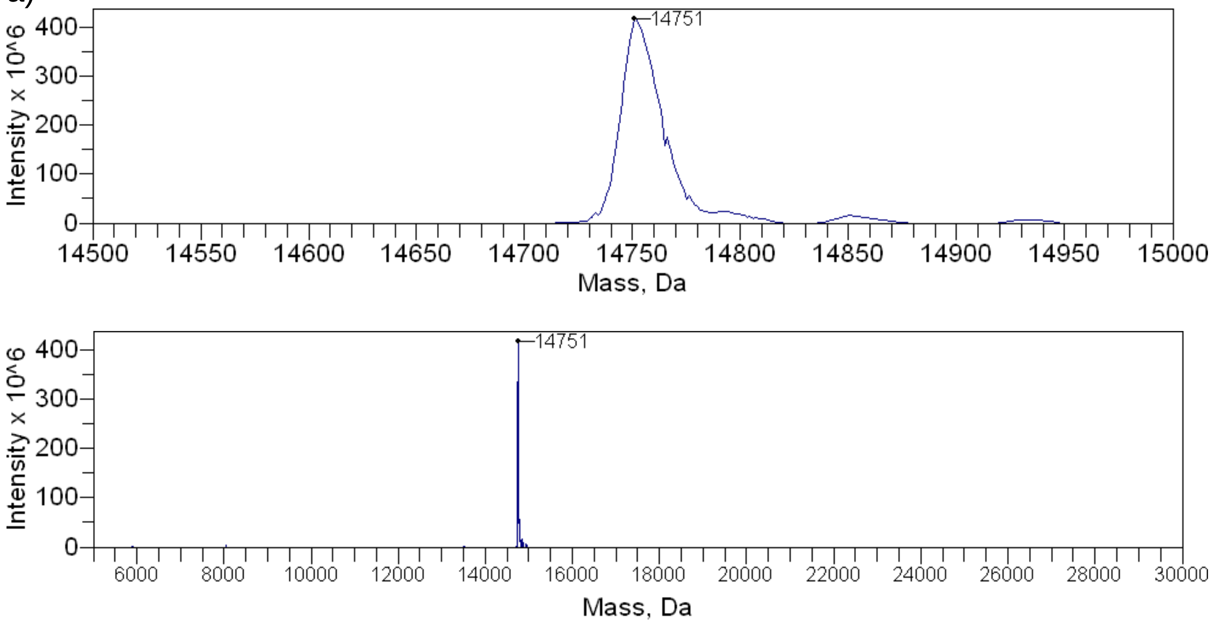

b)

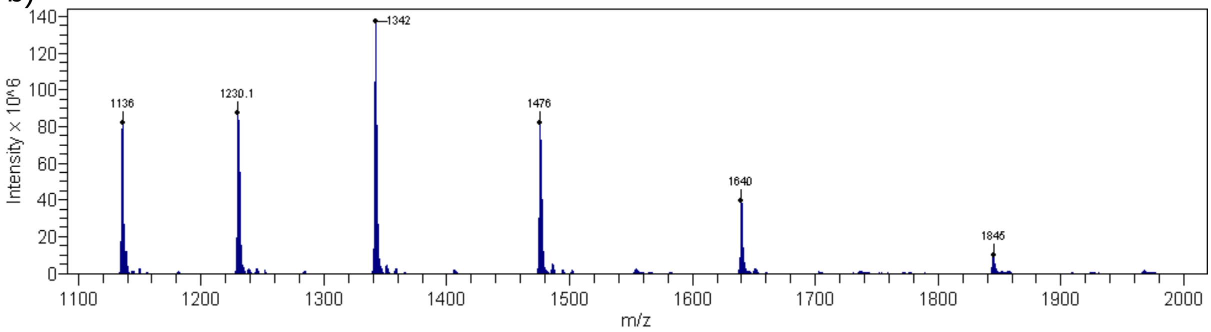

c)

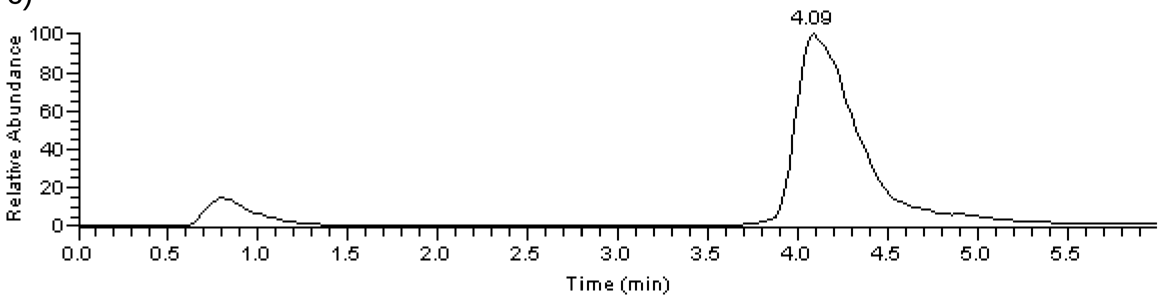

(a) deconvoluted, (b) non-deconvoluted, and (c) TIC mass spectrometry data for HER2DARPin(Nmut, N36C) reacted with NMM.

Supplementary Figure 10. HER2DARPin(D39C) in PBS, 1 mM EDTA. Expected mass 14584.4 Da

Sequence
MRGSHHHHHHGSDLGKKLLEAARAGQDDEVRILMANGA**C**VNAKDEYGLTPLYLATAHGHLEI
VEVLLKNGADVNAVDAIGFTPLHLAAFIGHLEIAEVLLKHGADVNAQDKFGKTAFDISIGNG
NEDLAEILQKLN

a)

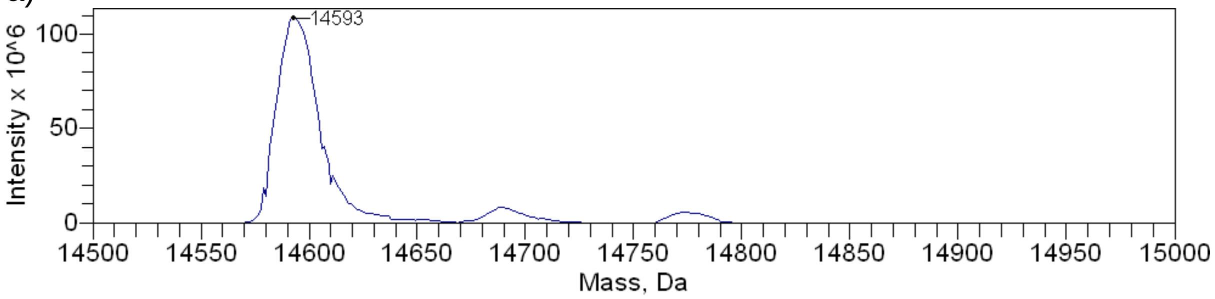

b)

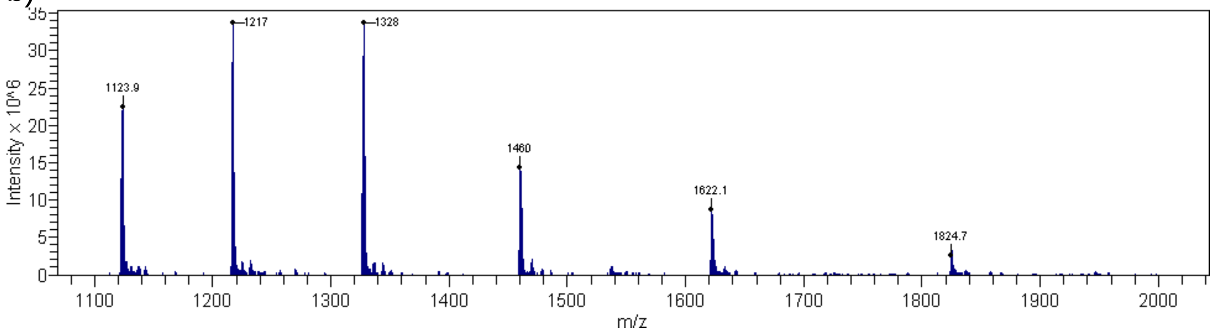

c)

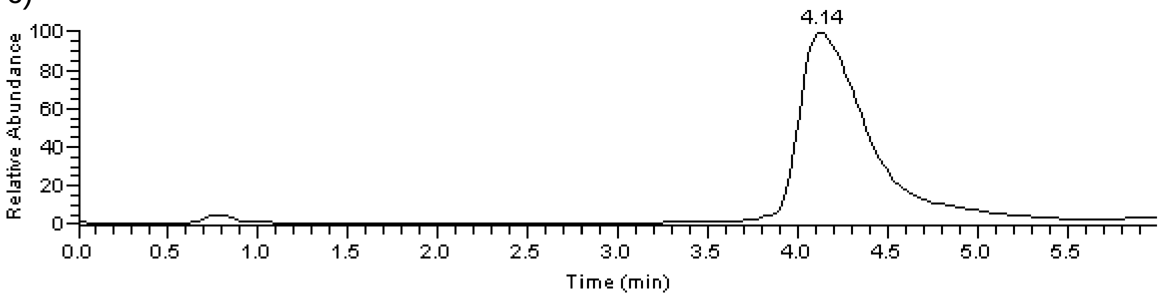

(a) deconvoluted, (b) non-deconvoluted, and (c) TIC mass spectrometry data for HER2DARPin(D39C).

Supplementary Figure 11. HER2DARPin(D39C) with 1 mM BrAcEGMe, 1 mM EDTA, in PBS pH 7.4, 10% DMF, 2 h, at 4 °C.
Expected mass (unmodified) 14584.4 Da
Expected mass (modified) 14699.6 Da

a)

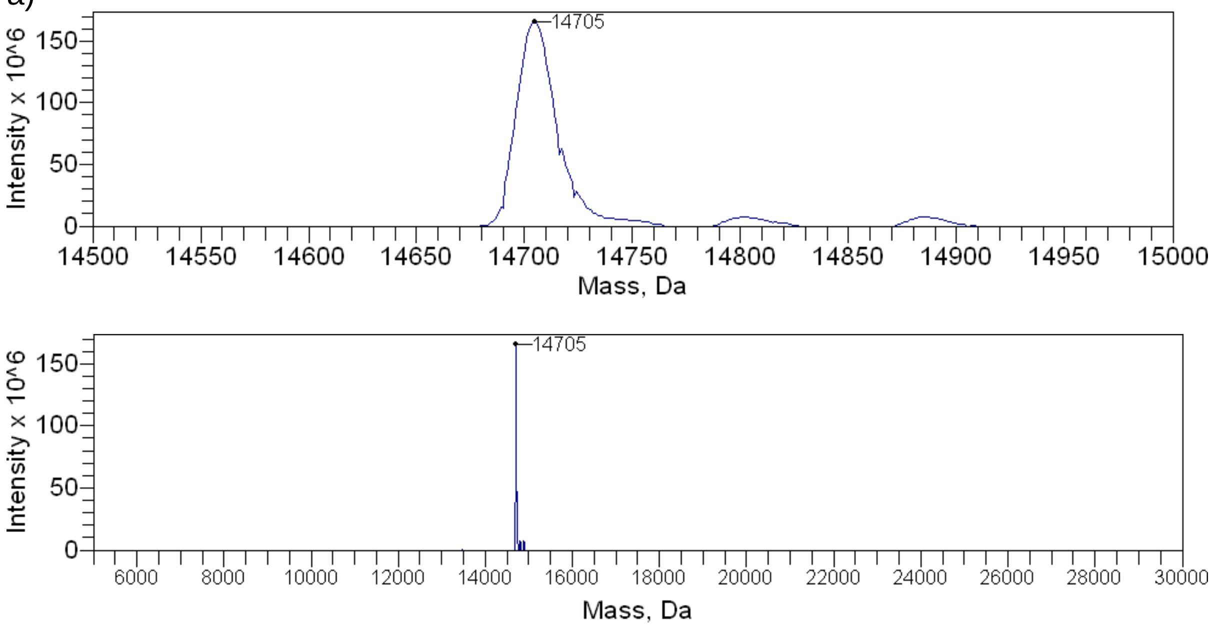

b)

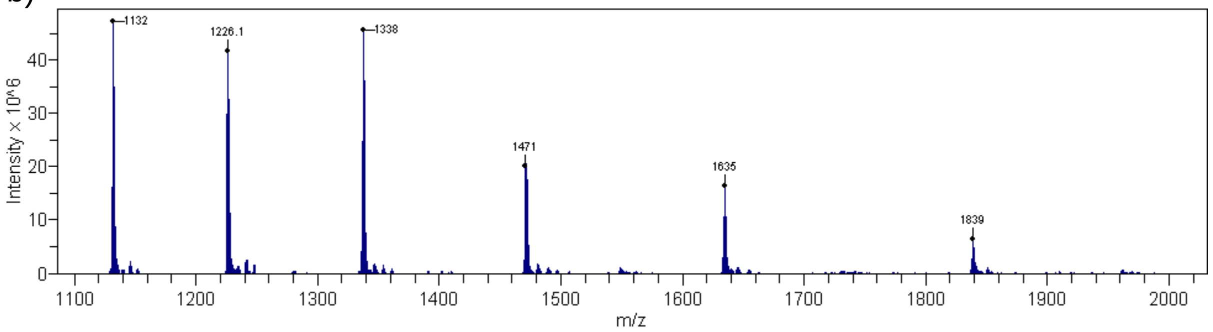

c)

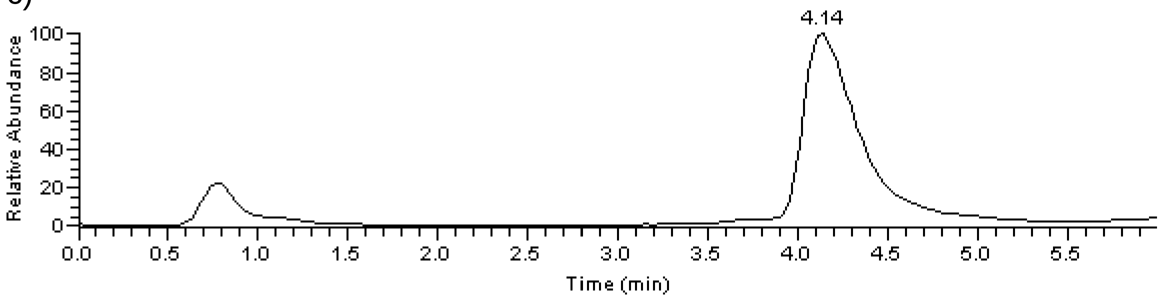

(a) deconvoluted, (b) non-deconvoluted, and (c) TIC mass spectrometry data for HER2DARPin(D39C) reacted with BrAcEGMe.

Supplementary Figure 12. HER2DARPin(D39C) with 1 mM NMM, 1 mM EDTA, in PBS pH 7.4, 10% DMF, 1 h, at 4 °C.
Expected mass (unmodified) 14584.4 Da
Expected mass (modified) 14695.5 Da

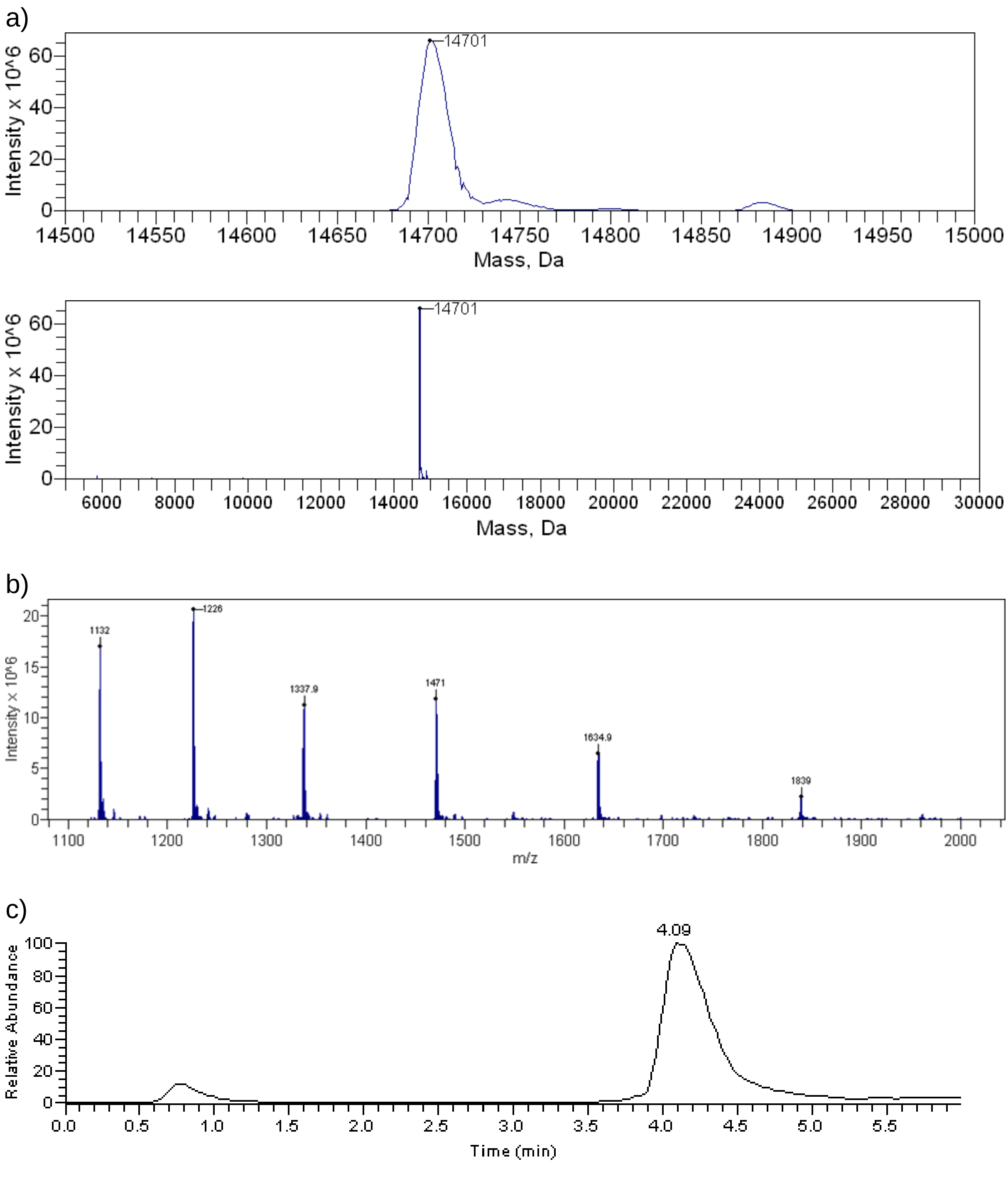

(a) deconvoluted, (b) non-deconvoluted, and (c) TIC mass spectrometry data for HER2DARPin(D39C) reacted with NMM.

Supplementary Figure 13. HER2DARPin(H59C) in PBS, 1 mM EDTA. Expected mass 14562.4 Da

Sequence

MRGSHHHHHHGSDLGKKLLEAARAGQDDEVRILMANGADVNAKDEYGLTPLYLATAHG**C**LEI
VEVLLKNGADVNAVDAIGFTPLHLAAFIGHLEIAEVLLKHGADVNAQDKFGKTAFDISIGNG
NEDLAEILQKLN

a)

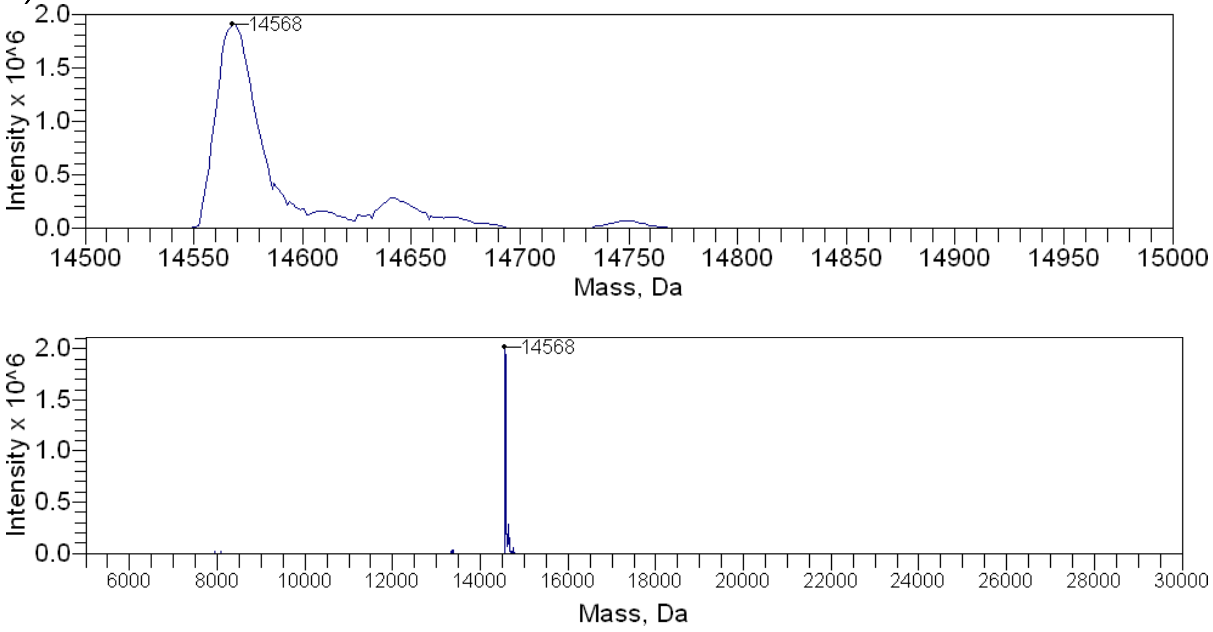

b)

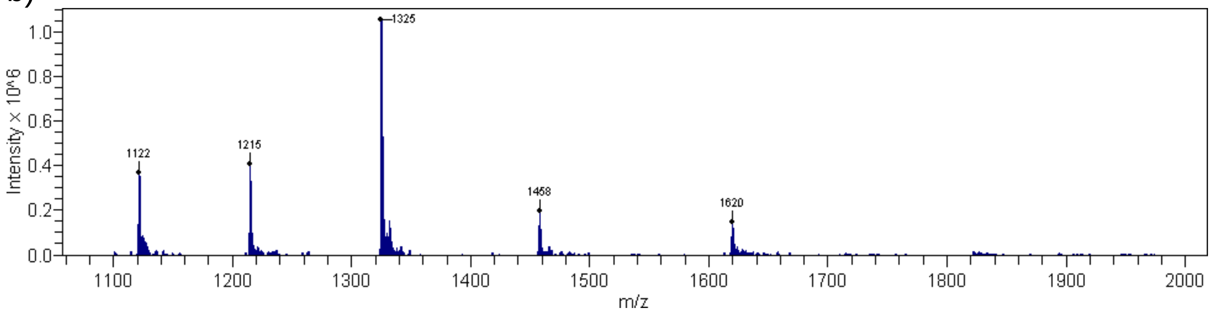

c)

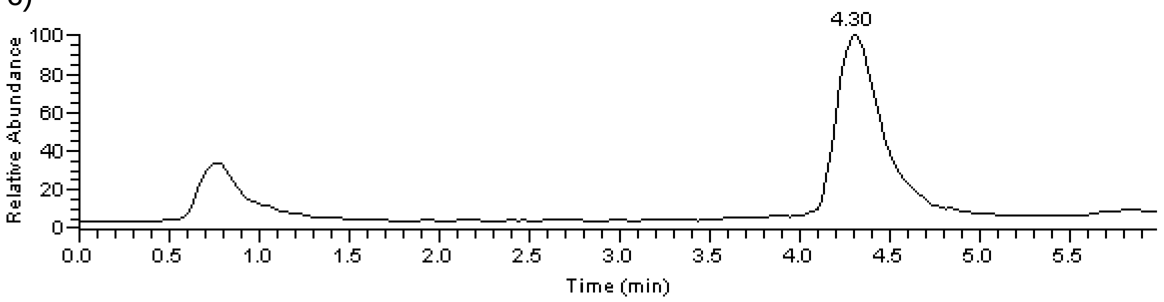

(a) deconvoluted, (b) non-deconvoluted, and (c) TIC mass spectrometry data for HER2DARPin(H59C).

Supplementary Figure 14. HER2DARPin(H59C) with 1 mM BrAcEGMe, 1 mM EDTA, in PBS pH 7.4, 10% DMF, 2 h, at 4 °C.
Expected mass (unmodified) 14562.4 Da
Expected mass (modified) 14677.6 Da

a)

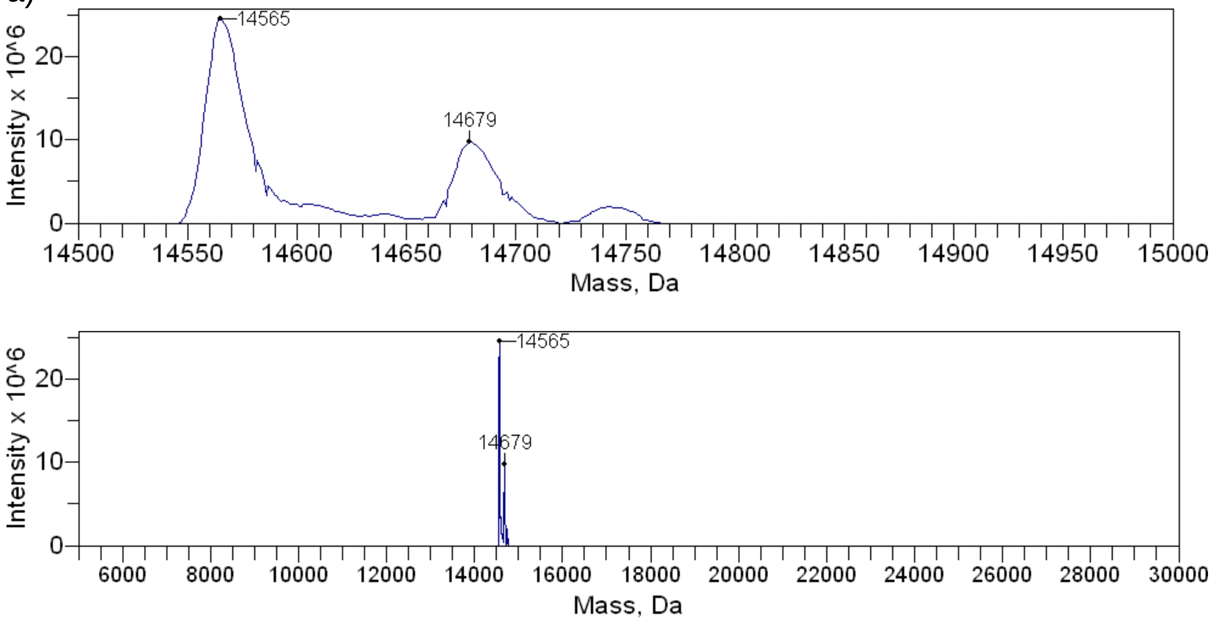

b)

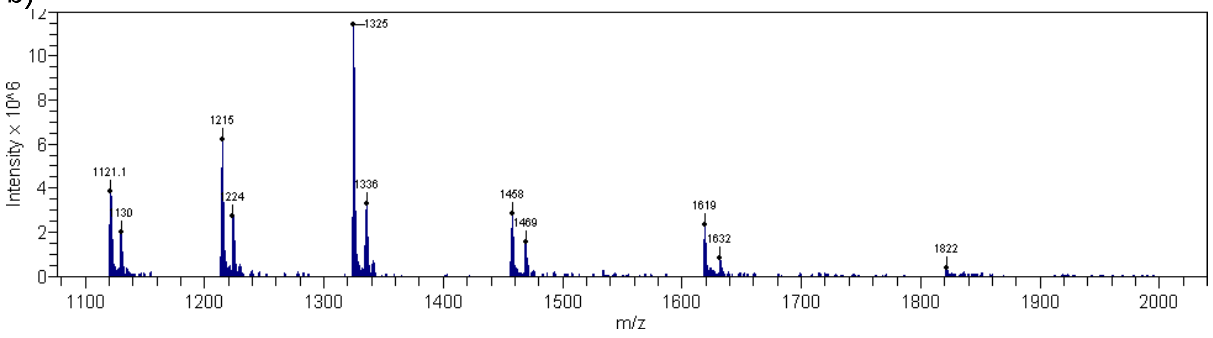

c)

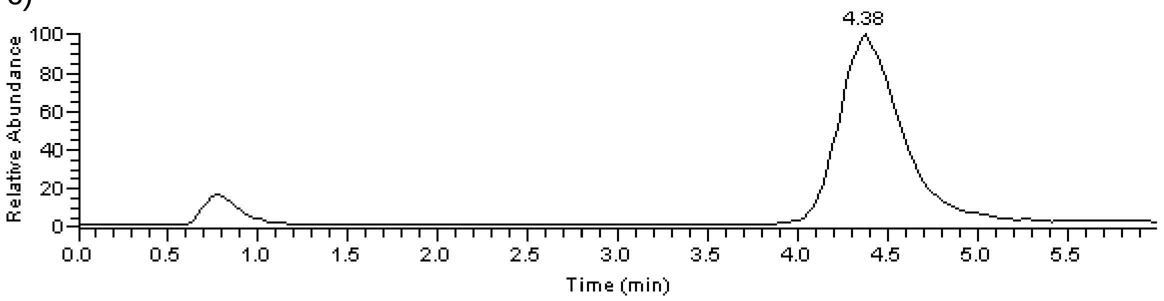

(a) deconvoluted, (b) non-deconvoluted, and (c) TIC mass spectrometry data for HER2DARPin(H59C) reacted with BrAcEGMe.

Supplementary Figure 15. HER2DARPin(H59C) with 1 mM NMM, 1 mM EDTA, in PBS pH 7.4, 10% DMF, 1 h, at 4 °C.
Expected mass (unmodified) 14562.4 Da
Expected mass (modified) 14673.5 Da

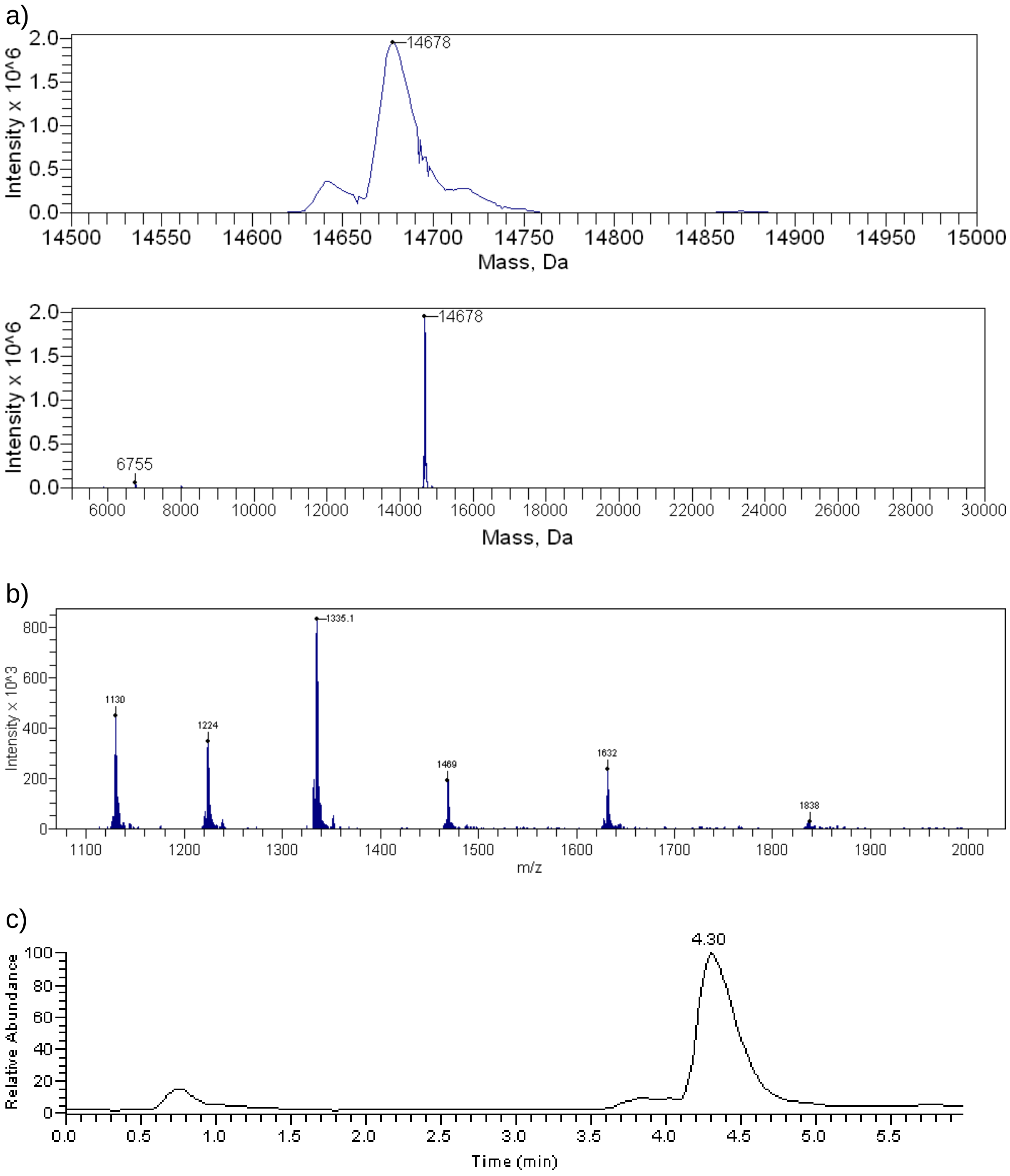

(a) deconvoluted, (b) non-deconvoluted, and (c) TIC mass spectrometry data for HER2DARPin(H59C) reacted with NMM.

Supplementary Figure 16. HER2DARPin(L60C) in PBS, 1 mM EDTA. Expected mass 14586.4 Da

Sequence

MRGSHHHHHHGSDLGKKLLEAARAGQDDEVRILMANGADVNAKDEYGLTPLYLATAHGHCEI
VEVLLKNGADVNAVDAIGFTPLHLAAFIGHLEIAEVLLKHGADVNAQDKFGKTAFDISIGNG
NEDLAEILQKLN

a)

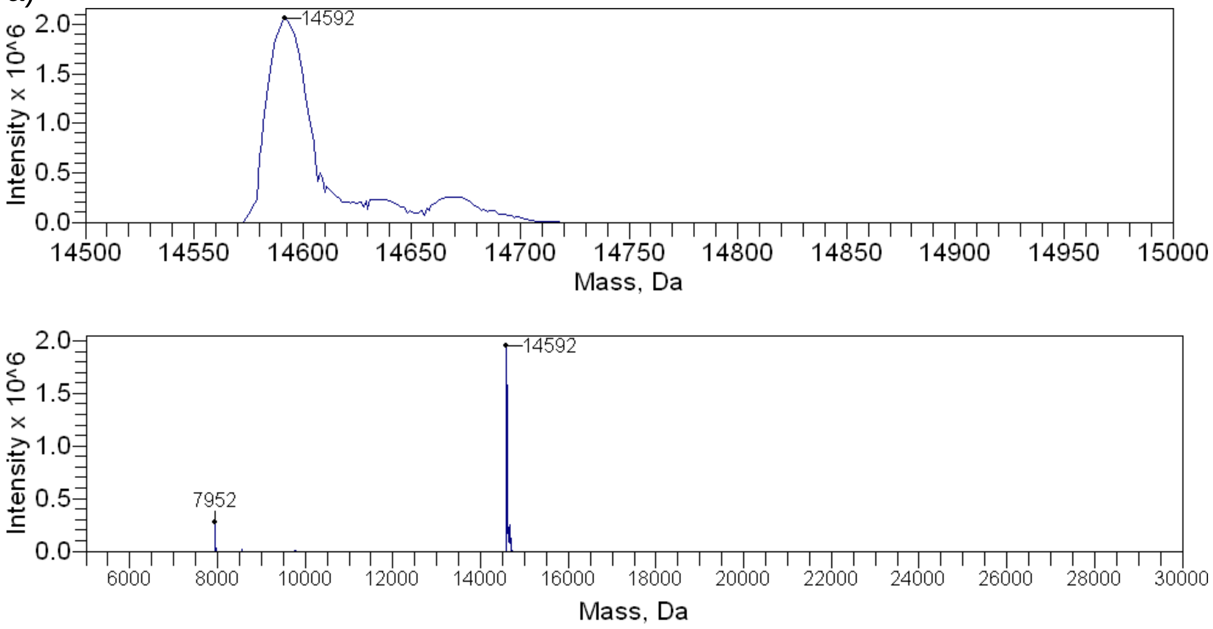

b)

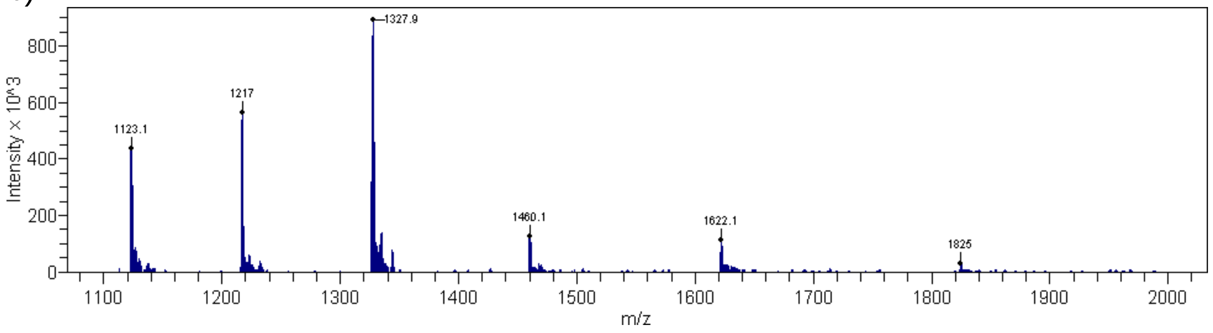

c)

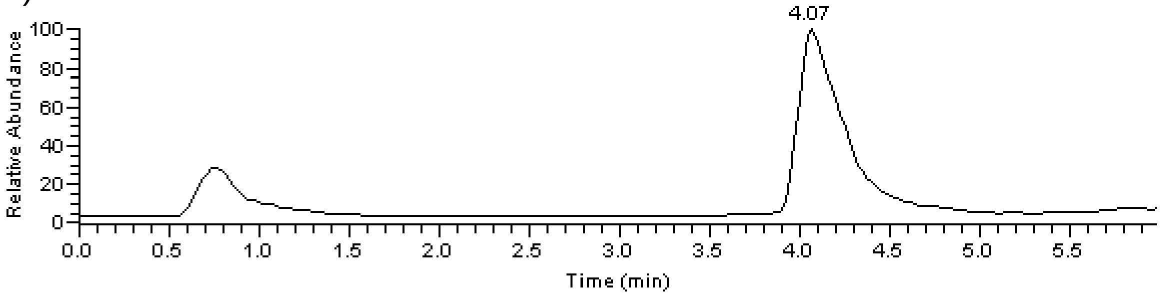

(a) deconvoluted, (b) non-deconvoluted, and (c) TIC mass spectrometry data for HER2DARPin(L60C).

Supplementary Figure 17. HER2DARPin(L60C) with 1 mM BrAcEGMe, 1 mM EDTA, in PBS pH 7.4, 10% DMF, 2 h, at 4 °C.
Expected mass (unmodified) 14586.4 Da
Expected mass (modified) 14601.6 Da

a)

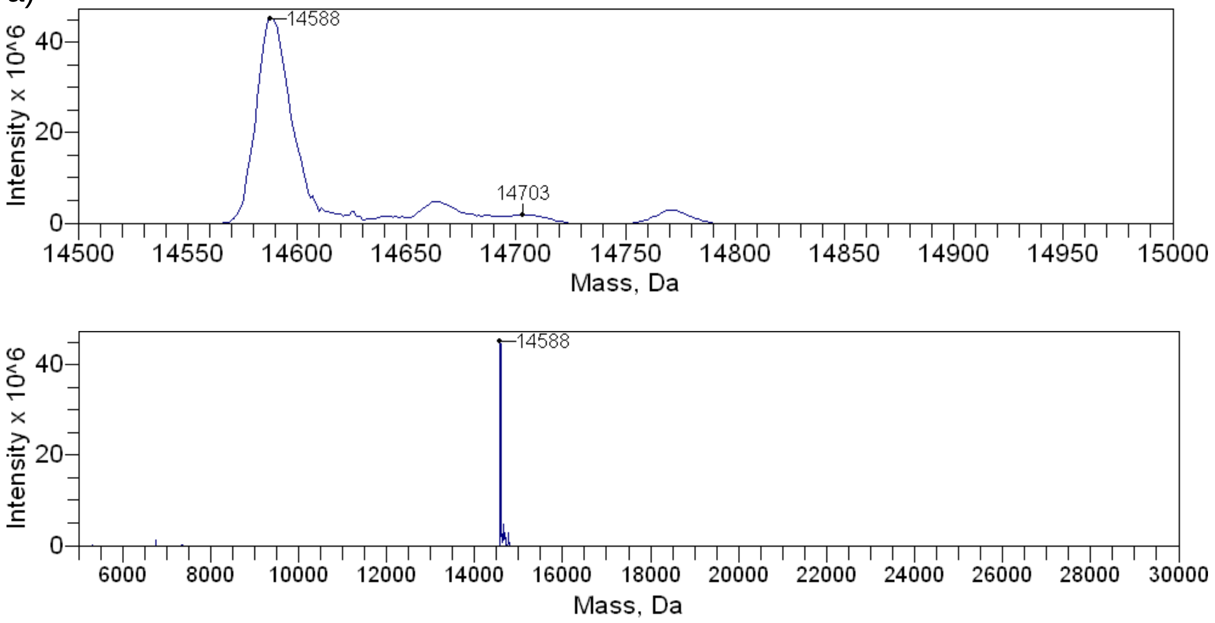

b)

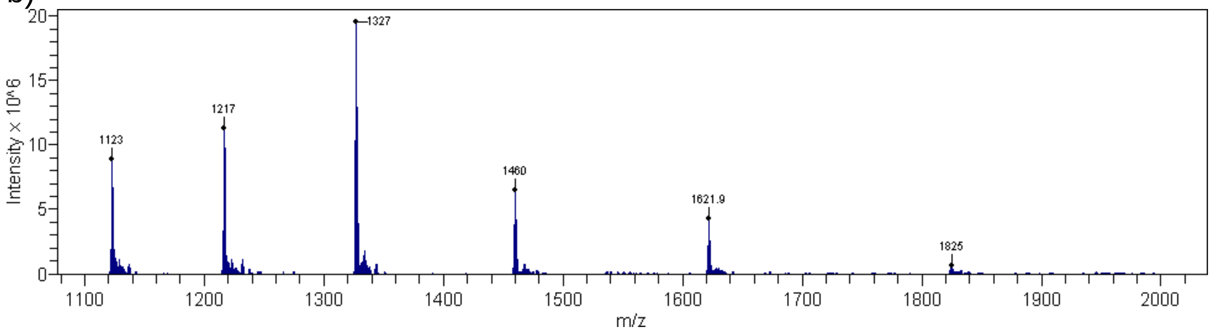

c)

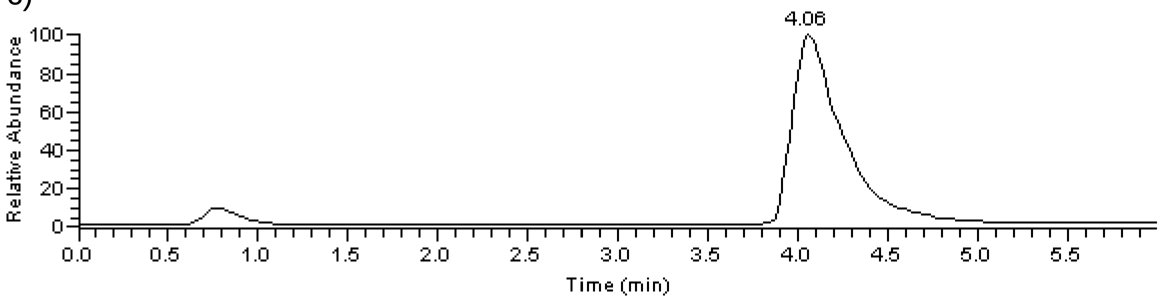

(a) deconvoluted, (b) non-deconvoluted, and (c) TIC mass spectrometry data for HER2DARPin(L60C) reacted with BrAcEGMe.

Supplementary Figure 18. HER2DARPin(L60C) with 1 mM NMM, 1 mM EDTA, in PBS pH 7.4, 10% DMF, 1 h, at 4 °C.
Expected mass (unmodified) 14586.4 Da
Expected mass (modified) 14597.5 Da

a)

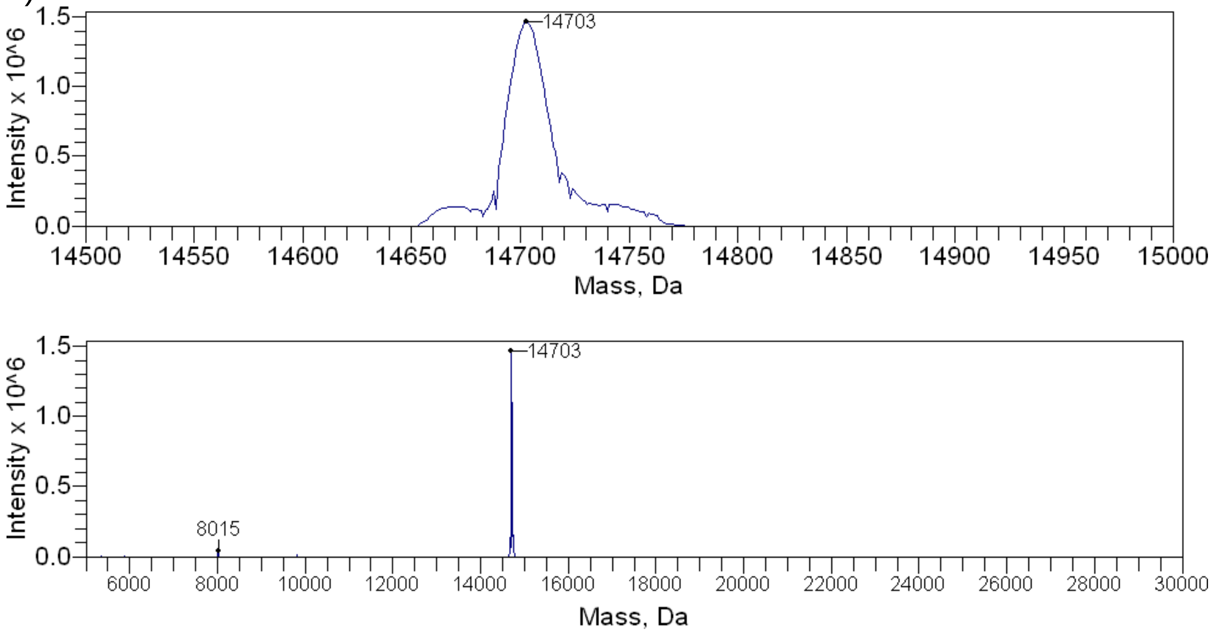

b)

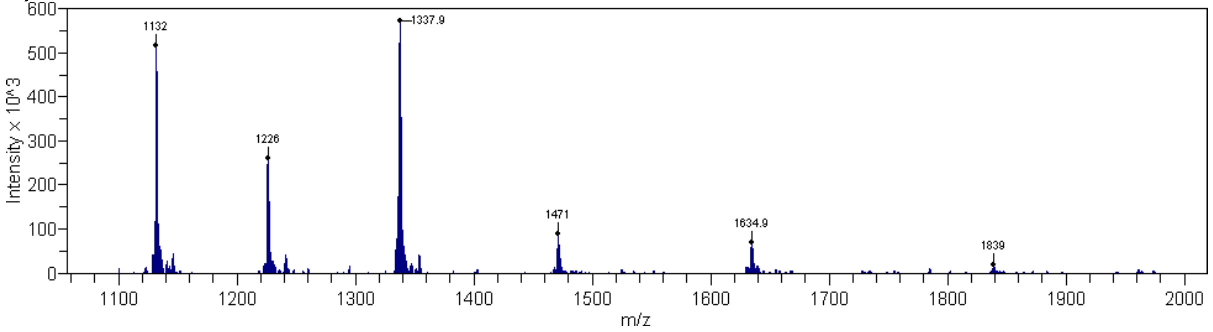

c)

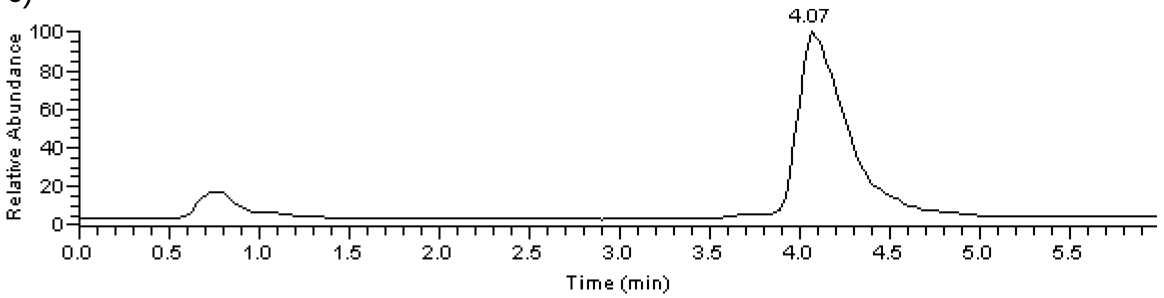

(a) deconvoluted, (b) non-deconvoluted, and (c) TIC mass spectrometry data for HER2DARPin(L60C) reacted with NMM.

Supplementary Figure 19. HER2DARPin(E61C) in PBS, 1 mM EDTA. Expected mass 14570.4 Da

Sequence
MRGSHHHHHHGSDLGKKLLEAARAGQDDEVRILMANGADVNAKDEYGLTPLYLATAHGHL**C**I
VEVLLKNGADVNAVDAIGFTPLHLAAFIGHLEIAEVLLKHGADVNAQDKFGKTAFDISIGNG
NEDLAEILQKLN

a)

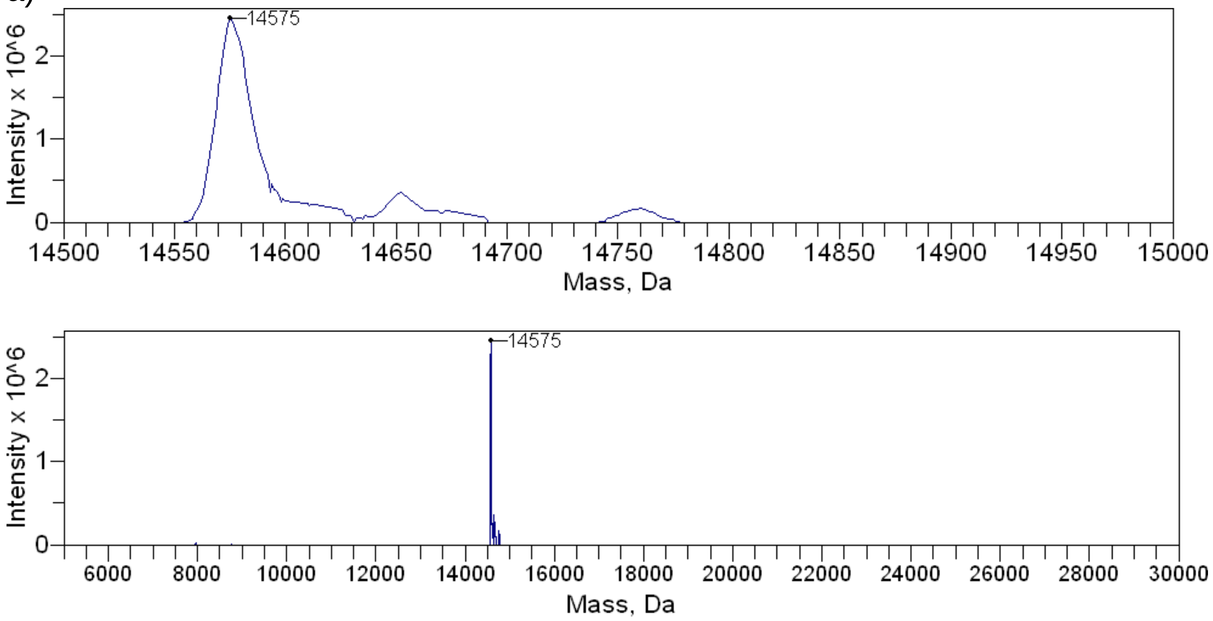

b)

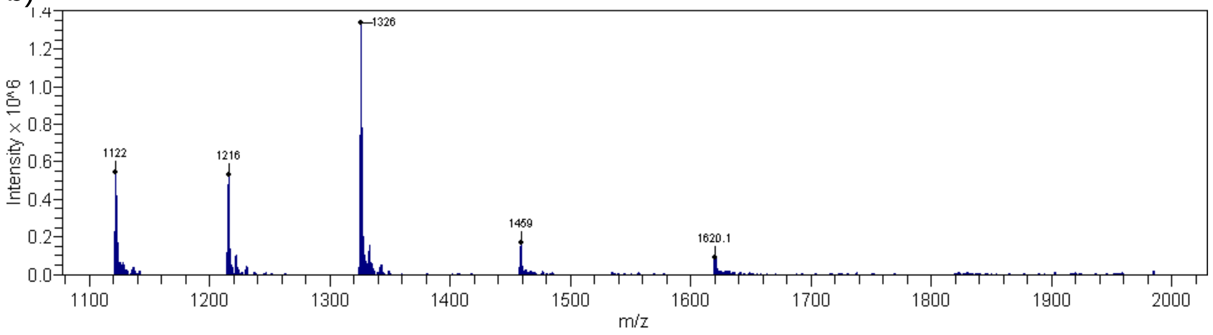

c)

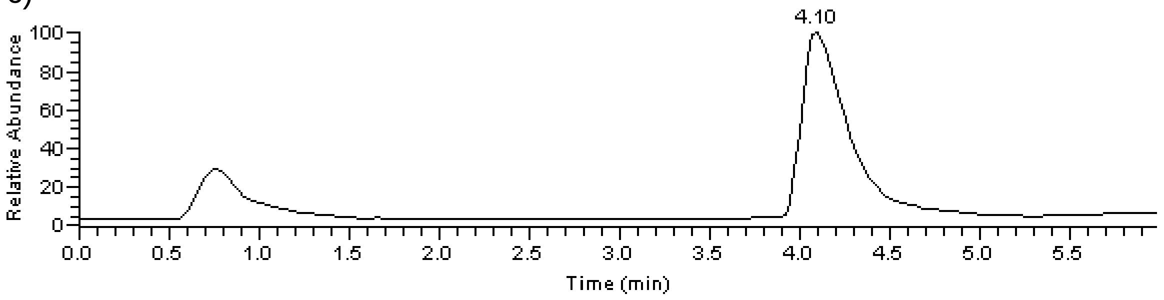

(a) deconvoluted, (b) non-deconvoluted, and (c) TIC mass spectrometry data for HER2DARPin(E61C).

Supplementary Figure 20. HER2DARPin(E61C) with 1 mM BrAcEGMe, 1 mM EDTA, in PBS pH 7.4, 10% DMF, 2 h, at 4 °C.
Expected mass (unmodified) 14570.4 Da
Expected mass (modified) 14685.6 Da

a)

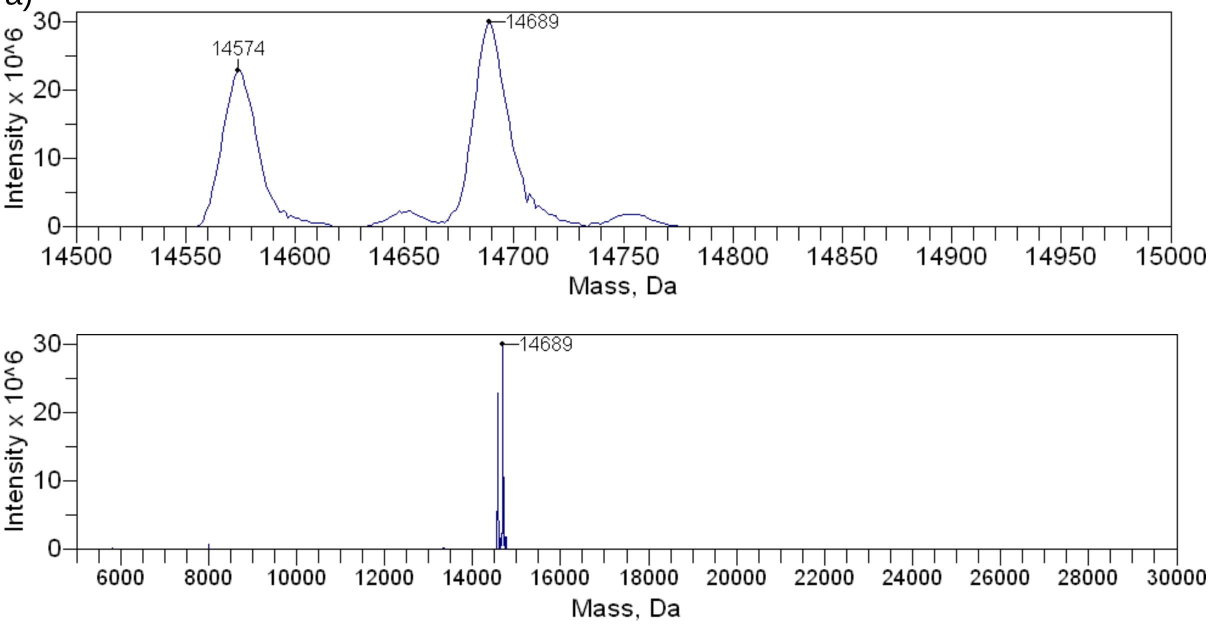

b)

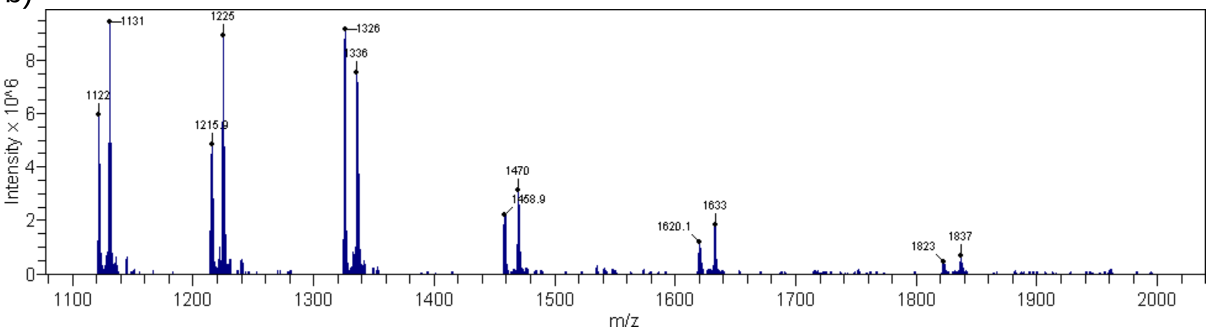

c)

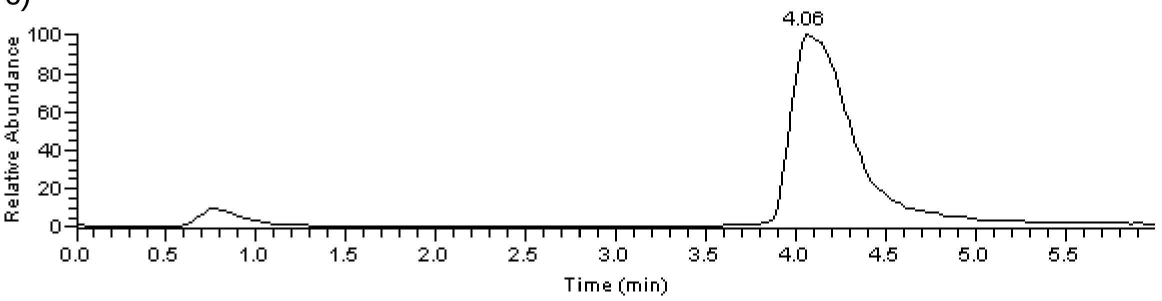

(a) deconvoluted, (b) non-deconvoluted, and (c) TIC mass spectrometry data for HER2DARPin(E61C) reacted with BrAcEGMe.

Supplementary Figure 21. HER2DARPin(E61C) with 1 mM NMM, 1 mM EDTA, in PBS pH 7.4, 10% DMF, 1 h, at 4 °C.
Expected mass (unmodified) 14570.4 Da
Expected mass (modified) 14681.5 Da

a)

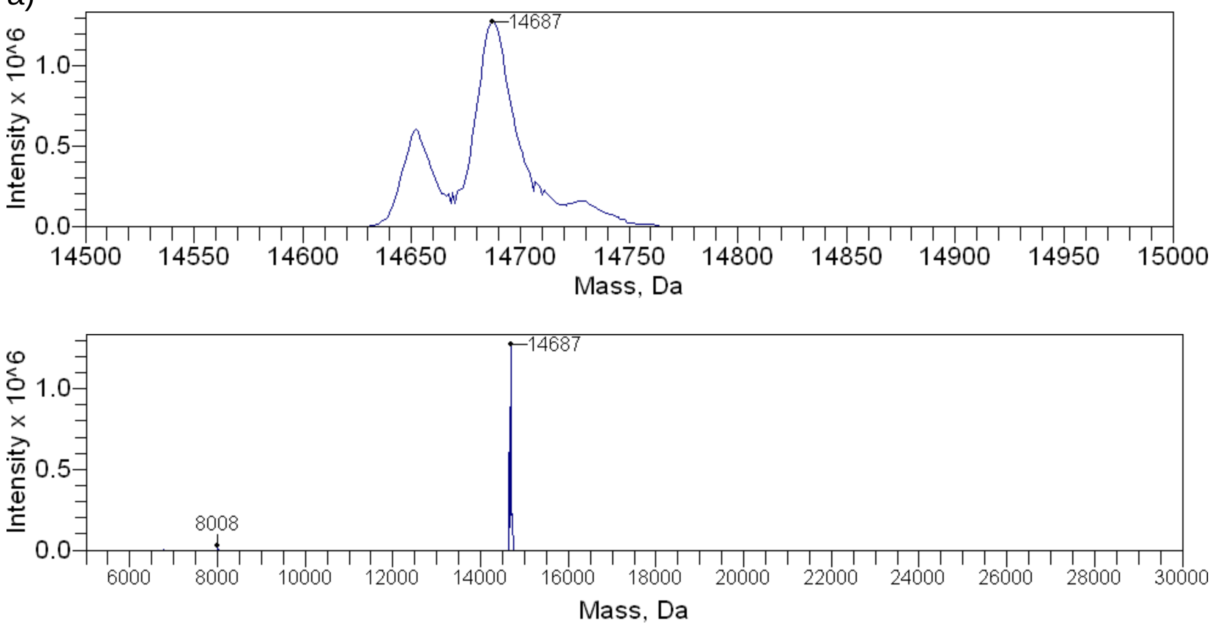

b)

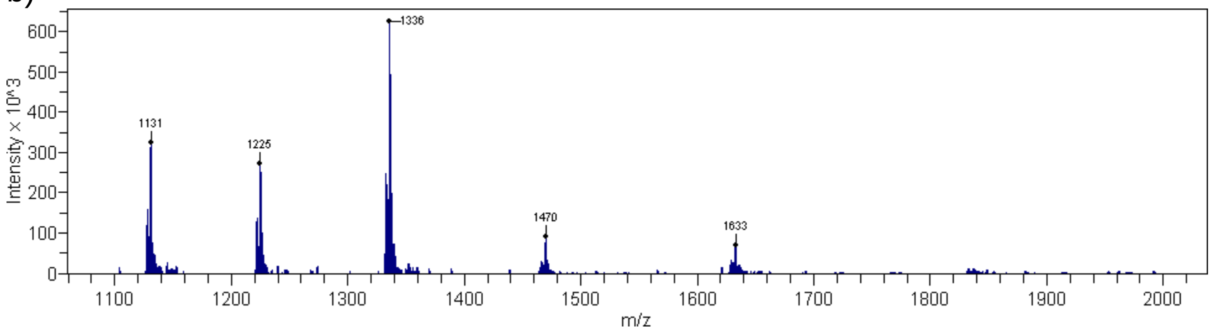

c)

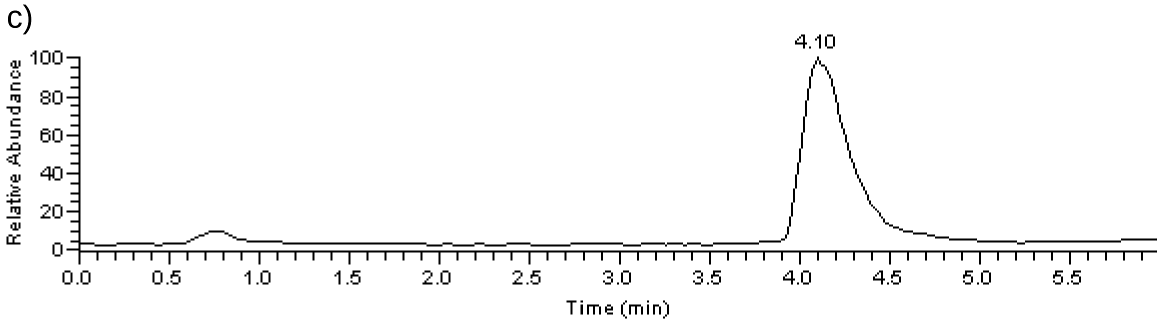

(a) deconvoluted, (b) non-deconvoluted, and (c) TIC mass spectrometry data for HER2DARPin(E61C) reacted with NMM.

Supplementary Figure 22. HER2DARPin(E64C) in PBS, 1 mM EDTA. Expected mass 14570.4 Da

Sequence
MRGSHHHHHHGSDLGKKLLEAARAGQDDEVRILMANGADVNAKDEYGLTPLYLATAHGHLEI
VCVLLKNGADVNAVDAIGFTPLHLAAFIGHLEIAEVLLKHGADVNAQDKFGKTAFDISIGNG
NEDLAEILQKLN

a)

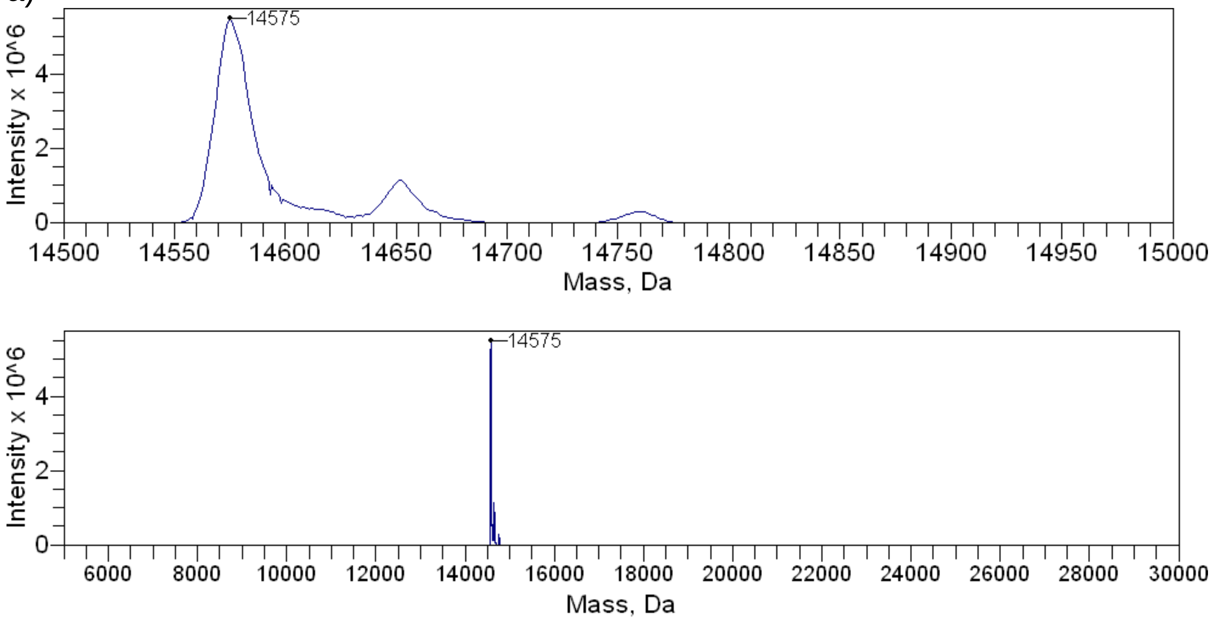

b)

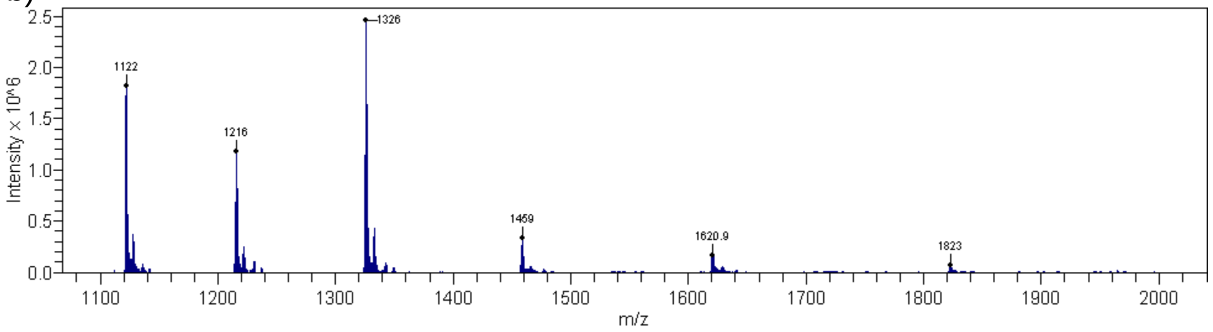

c)

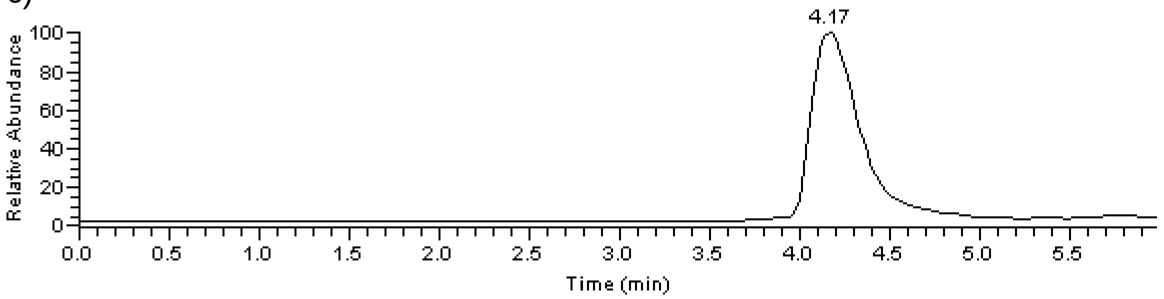

(a) deconvoluted, (b) non-deconvoluted, and (c) TIC mass spectrometry data for HER2DARPin(E64C).

Supplementary Figure 23. HER2DARPin(E64C) with 1 mM BrAcEGMe, 1 mM EDTA, in PBS pH 7.4, 10% DMF, 2 h, at 4 °C.
Expected mass (unmodified) 14570.4 Da
Expected mass (modified) 14685.6 Da

a)

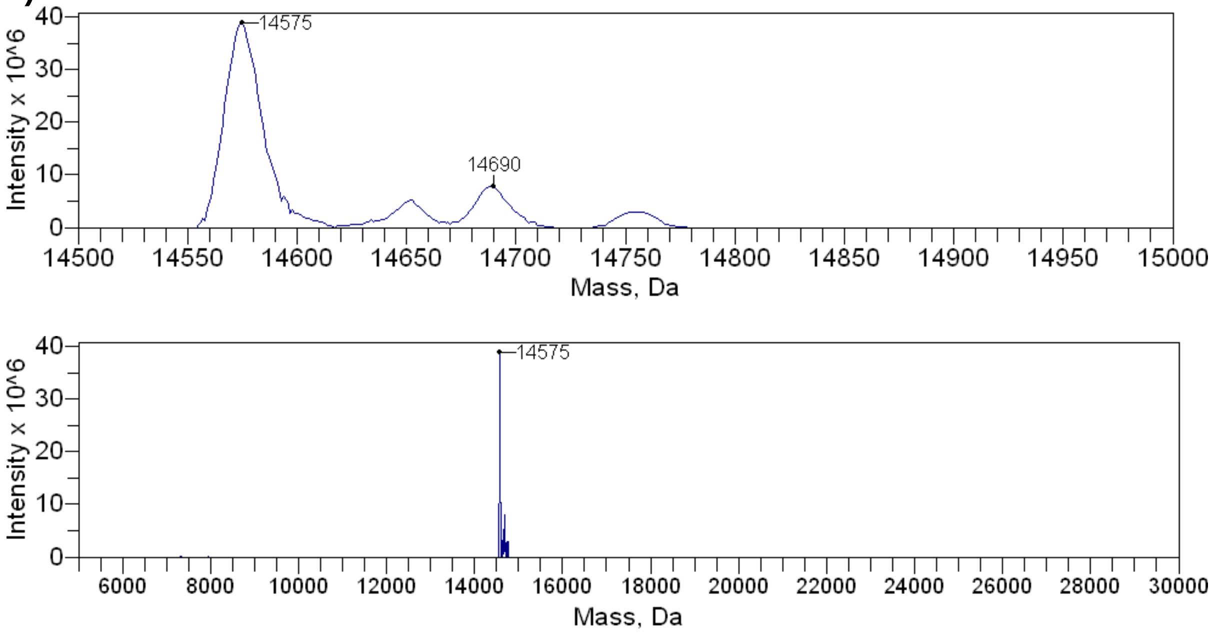

b)

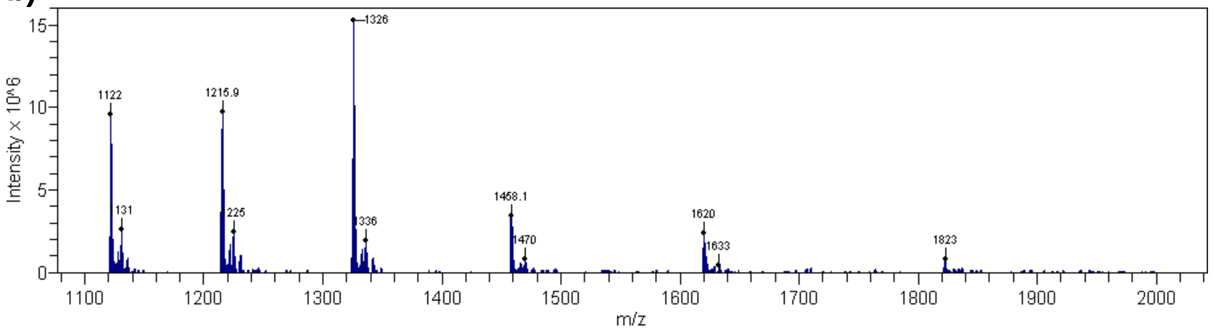

c)

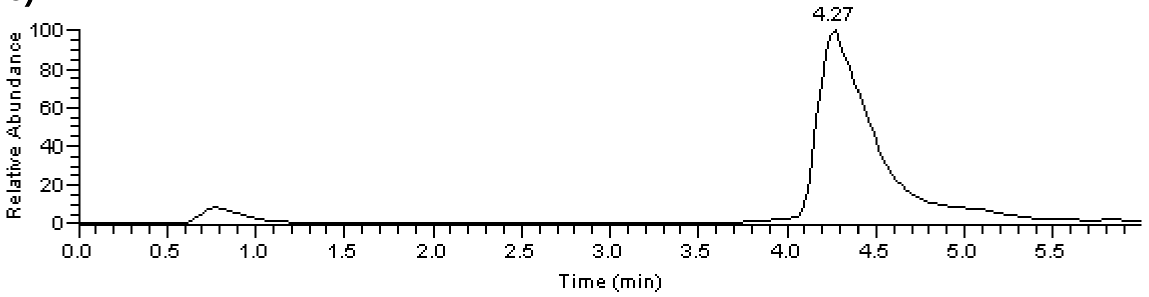

(a) deconvoluted, (b) non-deconvoluted, and (c) TIC mass spectrometry data for HER2DARPin(E64C) reacted with BrAcEGMe.

Supplementary Figure 24. HER2DARPin(E64C) with 1 mM NMM, 1 mM EDTA, in PBS pH 7.4, 10% DMF, 1 h, at 4 °C.
Expected mass (unmodified) 14570.4 Da
Expected mass (modified) 14681.5 Da

a)

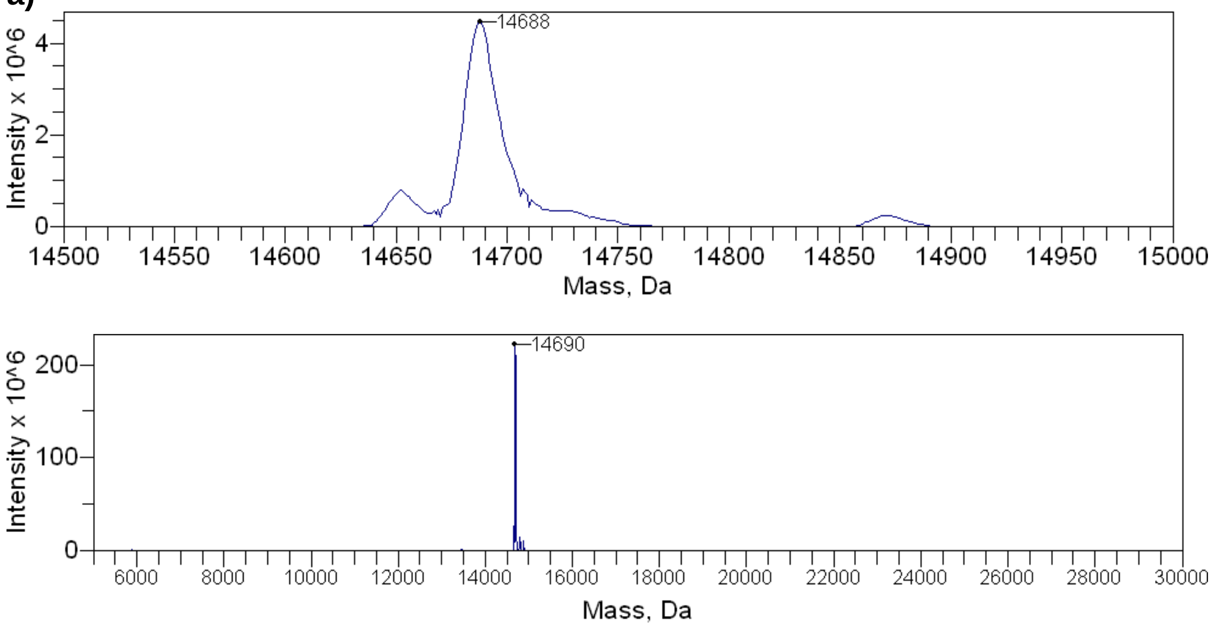

b)

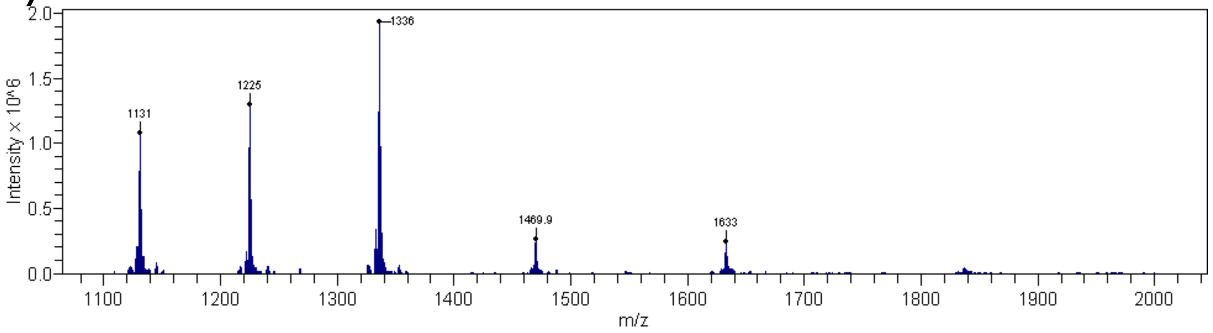

c)

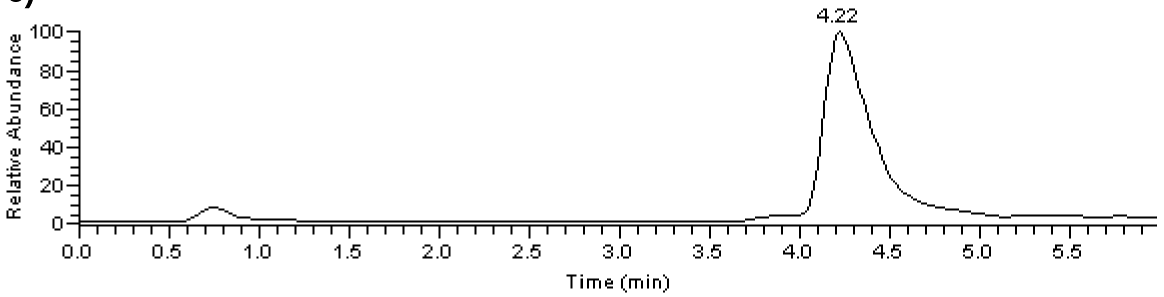

(a) deconvoluted, (b) non-deconvoluted, and (c) TIC mass spectrometry data for HER2DARPin(E64C) reacted with NMM.

Supplementary Figure 25. HER2DARPin(V65C) in PBS, 1 mM EDTA. Expected mass 14600.4 Da

Sequence

```
MRGSHHHHHHGSDLGKKLLEAARAGQDDEVRILMANGADVNAKDEYGLTPLYLATAHGHLEI
VECLLKNGADVNAVDAIGFTPLHLAAFIGHLEIAEVLLKHGADVNAQDKFGKTAFDISIGNG
NEDLAEILQKLN
```

a)

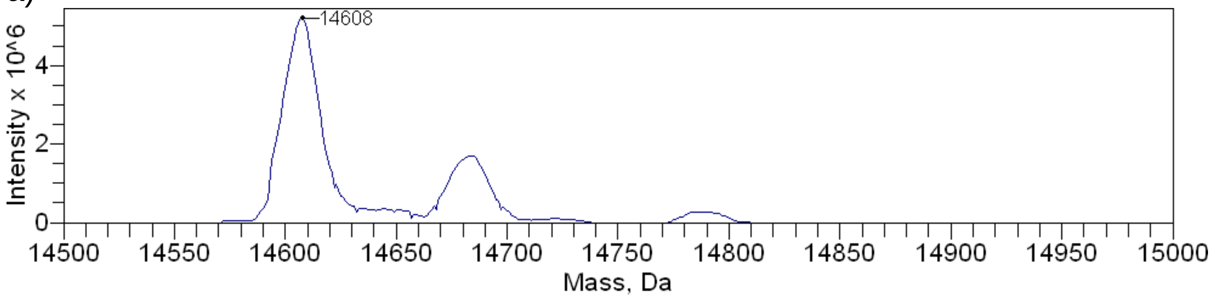

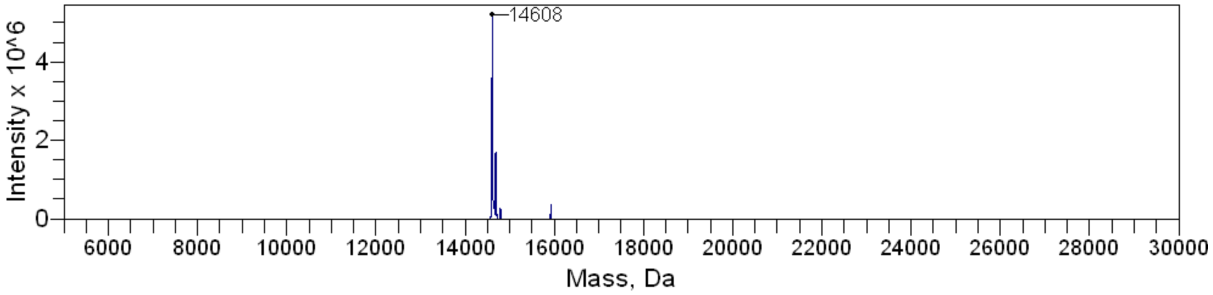

b)

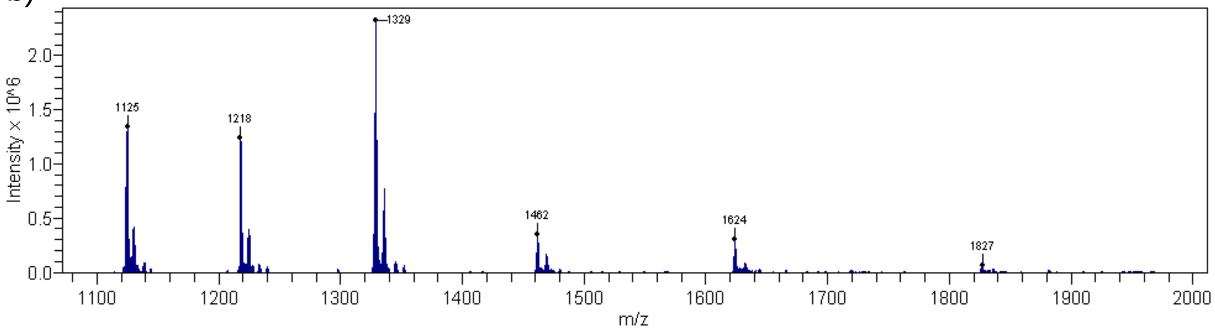

c)

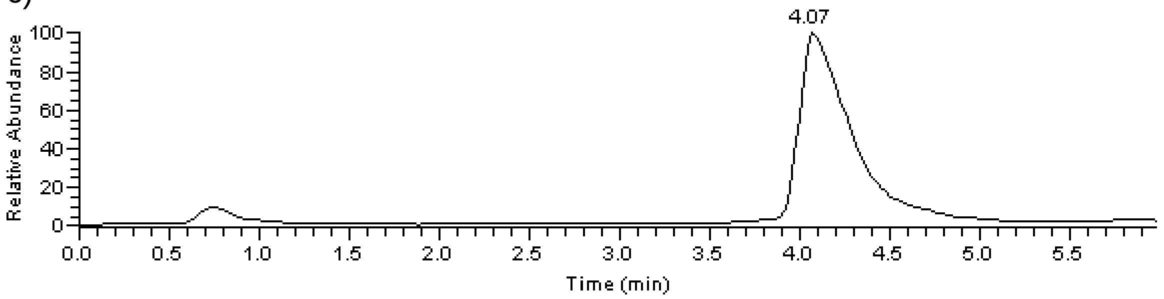

(a) deconvoluted, (b) non-deconvoluted, and (c) TIC mass spectrometry data for HER2DARPin(V65C).

Supplementary Figure 26. HER2DARPin(V65C) with 1 mM BrAcEGMe, 1 mM EDTA, in PBS pH 7.4, 10% DMF, 2 h, at 4 °C.
Expected mass (unmodified) 14600.4 Da
Expected mass (modified) 14715.6 Da

a)

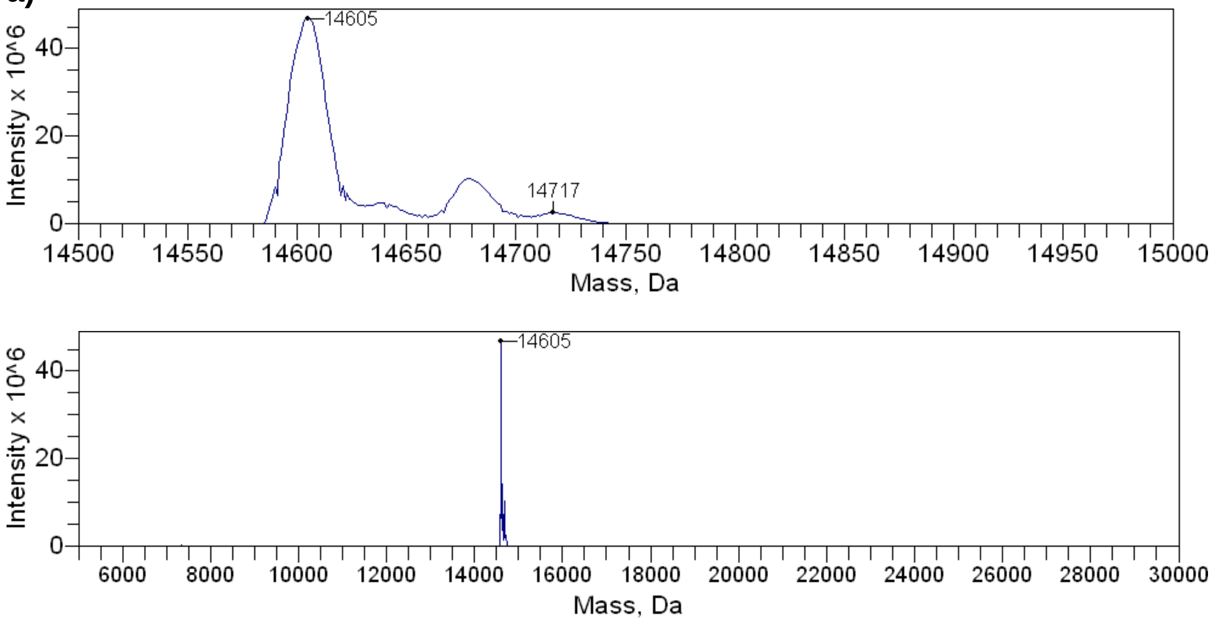

b)

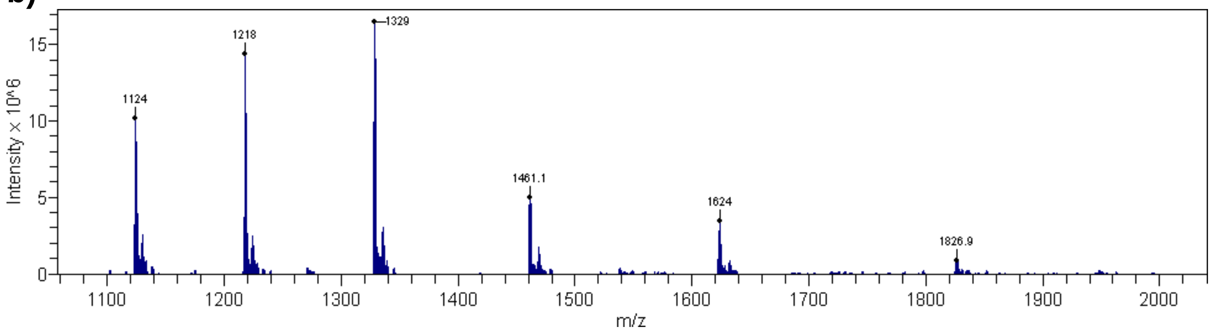

c)

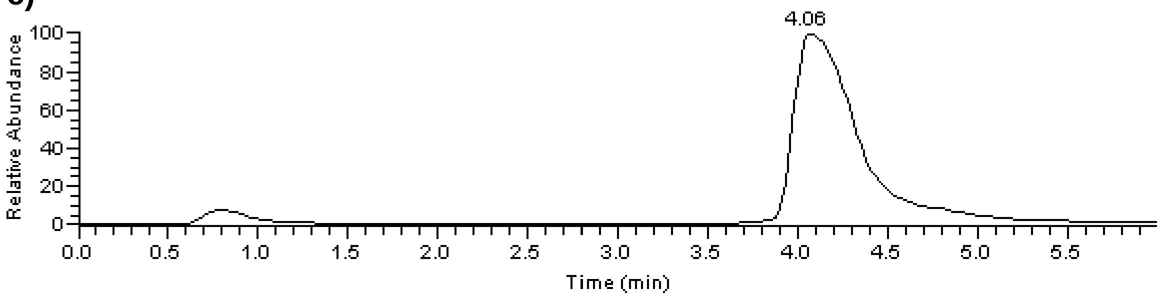

(a) deconvoluted, (b) non-deconvoluted, and (c) TIC mass spectrometry data for HER2DARPin(V65C) reacted with BrAcEGMe.

Supplementary Figure 27. HER2DARPin(V65C) with 1 mM NMM, 1 mM EDTA, in PBS pH 7.4, 10% DMF, 1 h, at 4 °C.
Expected mass (unmodified) 14600.4 Da
Expected mass (modified) 14711.5 Da

a)

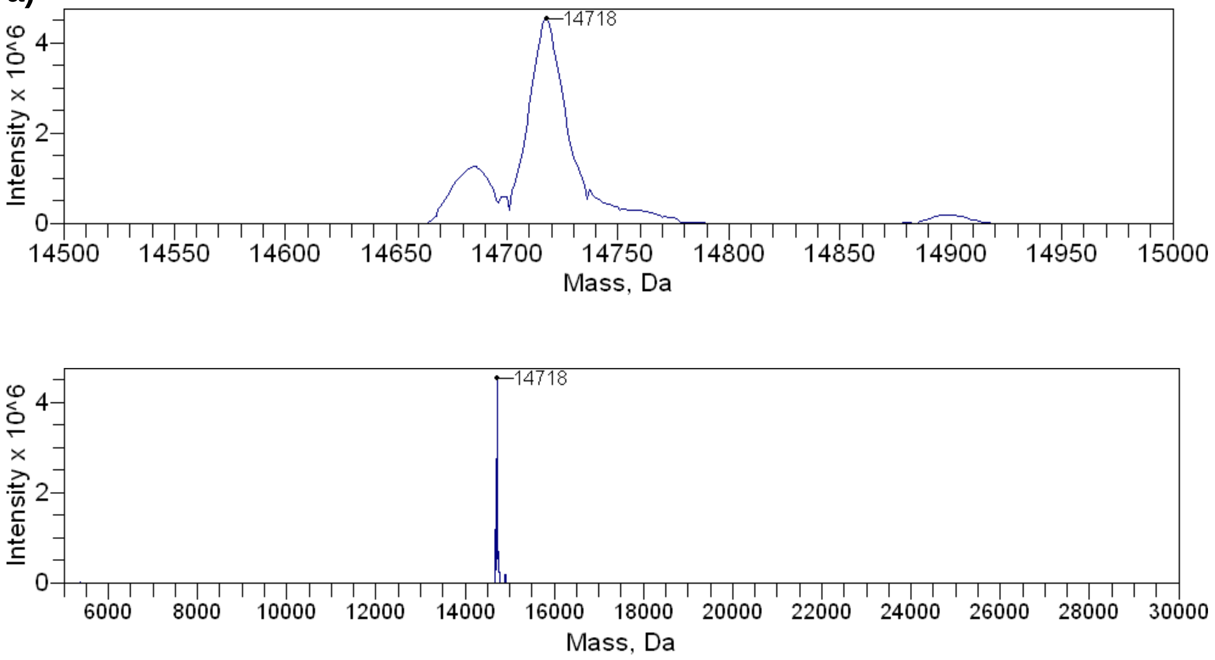

b)

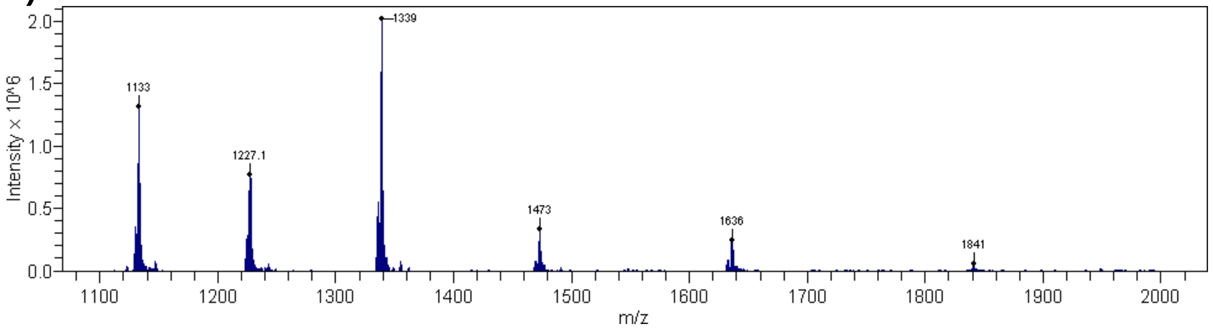

c)

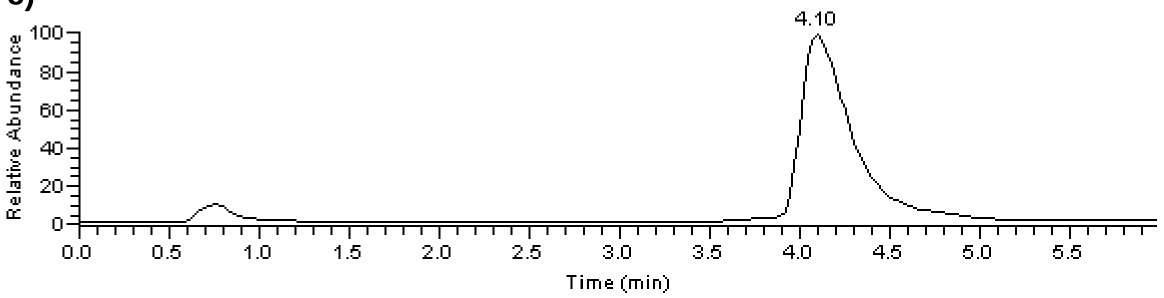

(a) deconvoluted, (b) non-deconvoluted, and (c) TIC mass spectrometry data for HER2DARPin(V65C) reacted with NMM.

Supplementary Figure 28. HER2DARPin(K68C) in PBS, 1 mM EDTA. Expected mass 14571.4 Da

Sequence
MRGSHHHHHHGSDLGKKLLEAARAGQDDEVRILMANGADVNAKDEYGLTPLYLATAHGHLEI
VEVLL**C**NGADVNAVDAIGFTPLHLAAFIGHLEIAEVLLKHGADVNAQDKFGKTAFDISIGNG
NEDLAEILQKLN

a)

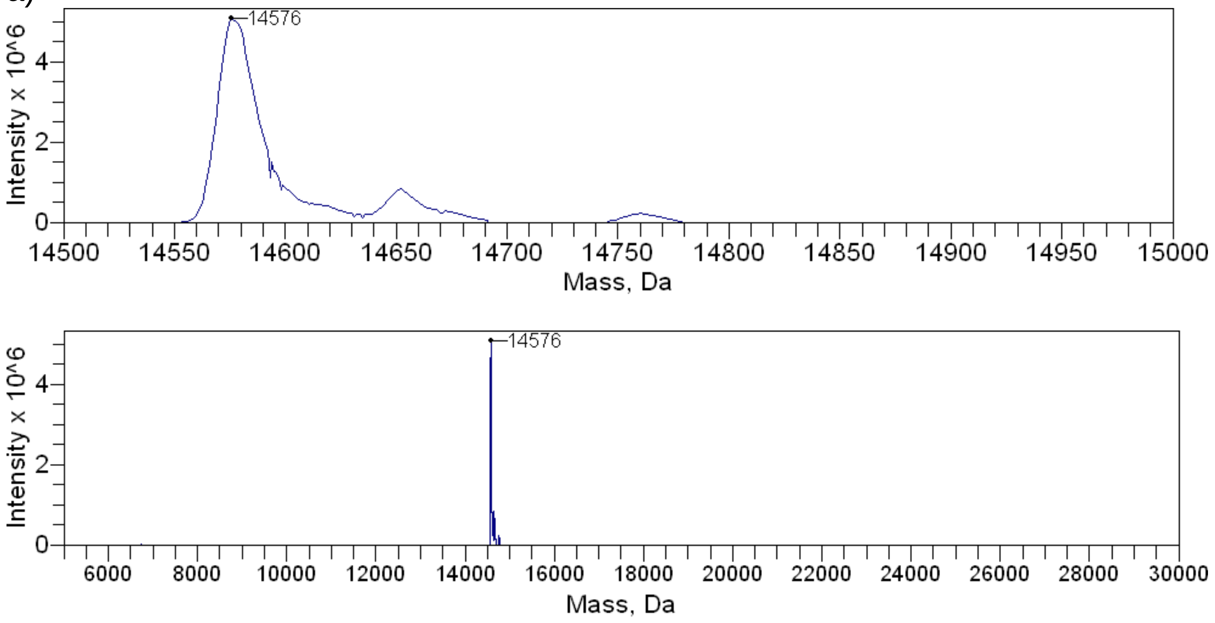

b)

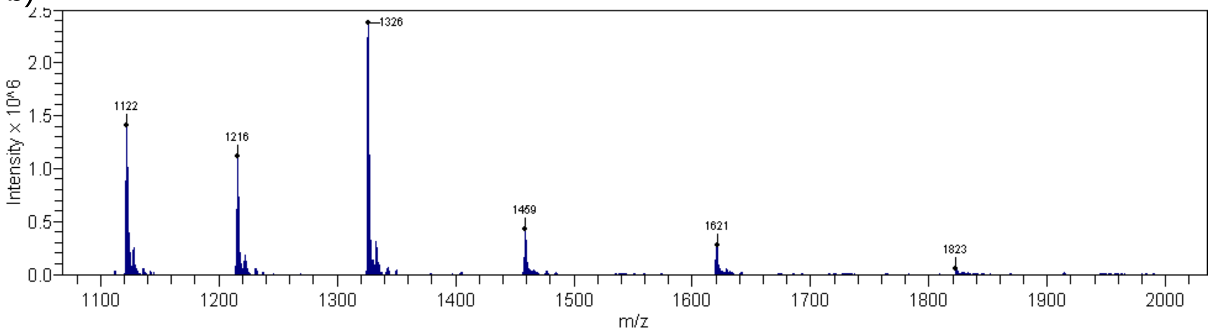

c)

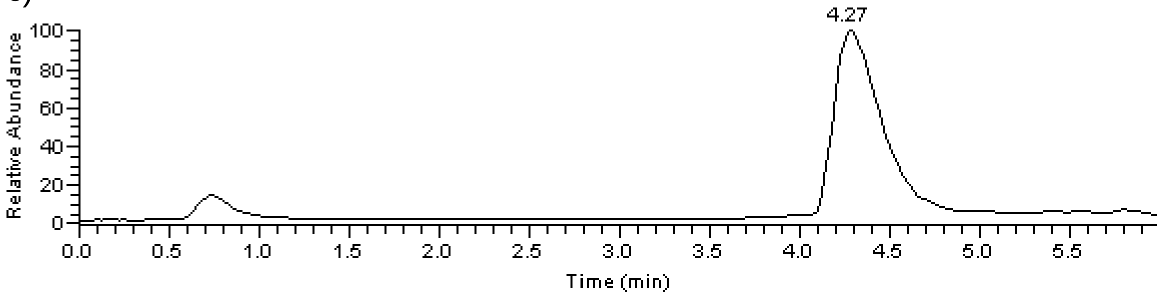

(a) deconvoluted, (b) non-deconvoluted, and (c) TIC mass spectrometry data for HER2DARPin(K68C).

Supplementary Figure 29. HER2DARPin(K68C) with 1 mM BrAcEGMe, 1 mM EDTA, in PBS pH 7.4, 10% DMF, 2 h, at 4 °C.
Expected mass (unmodified) 14571.4 Da
Expected mass (modified) 14686.6 Da

a)

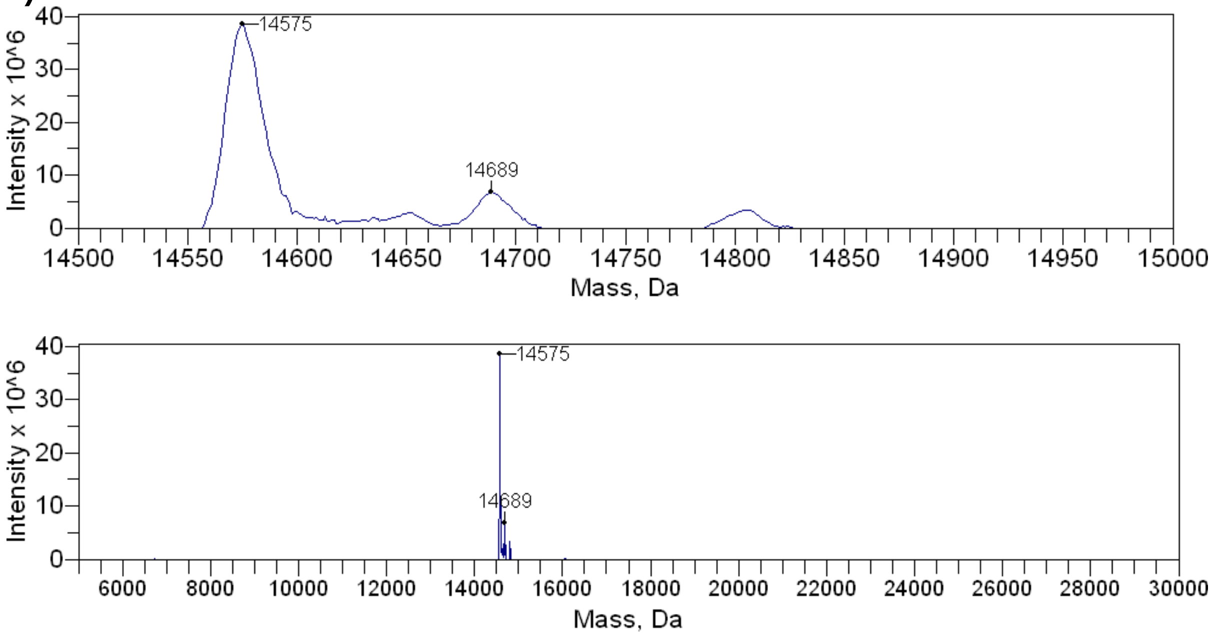

b)

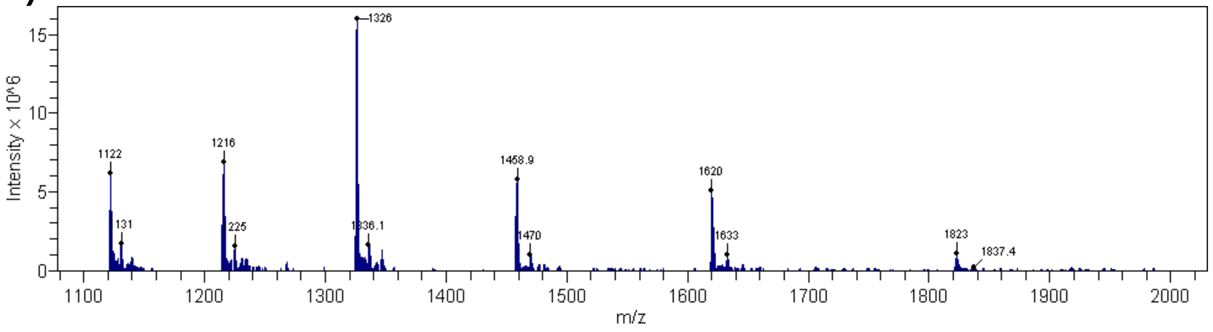

c)

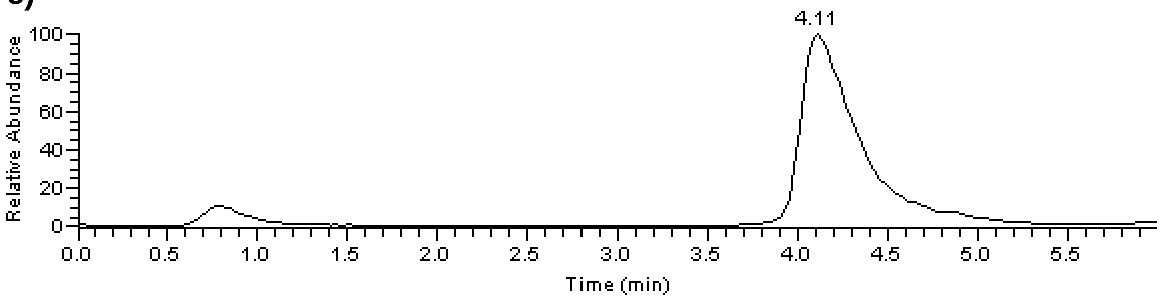

(a) deconvoluted, (b) non-deconvoluted, and (c) TIC mass spectrometry data for HER2DARPin(K68C) reacted with BrAcEGMe.

Supplementary Figure 30. HER2DARPin(K68C) with 1 mM NMM, 1 mM EDTA, in PBS pH 7.4, 10% DMF, 1 h, at 4 °C.
Expected mass (unmodified) 14571.4 Da
Expected mass (modified) 14682.5 Da

a)

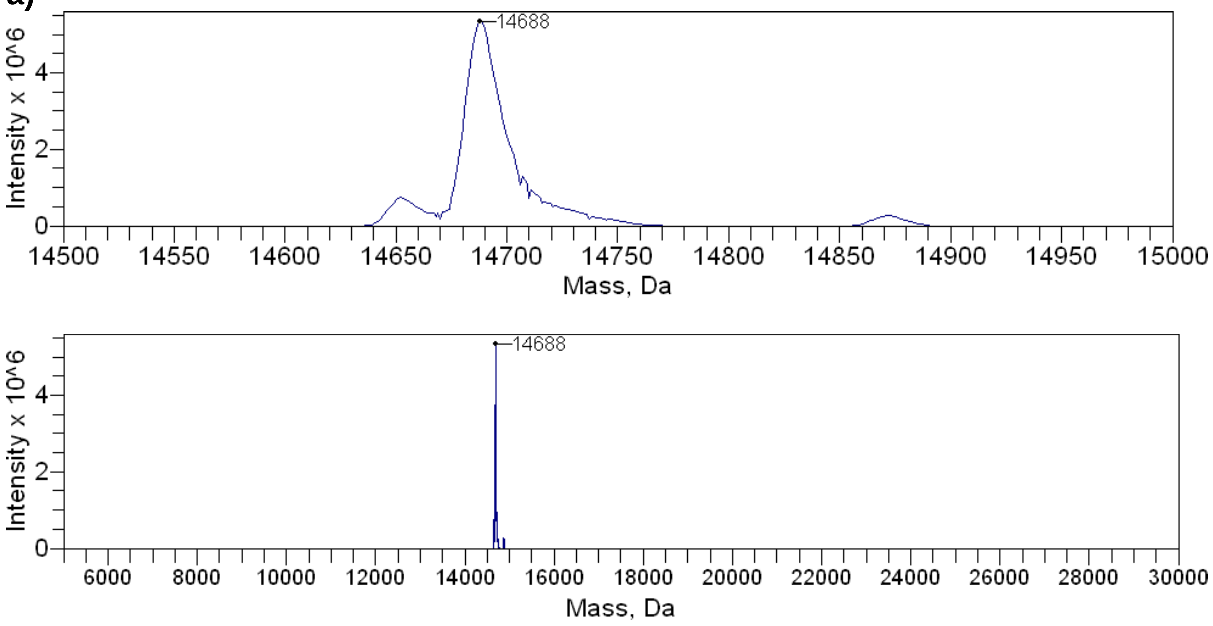

b)

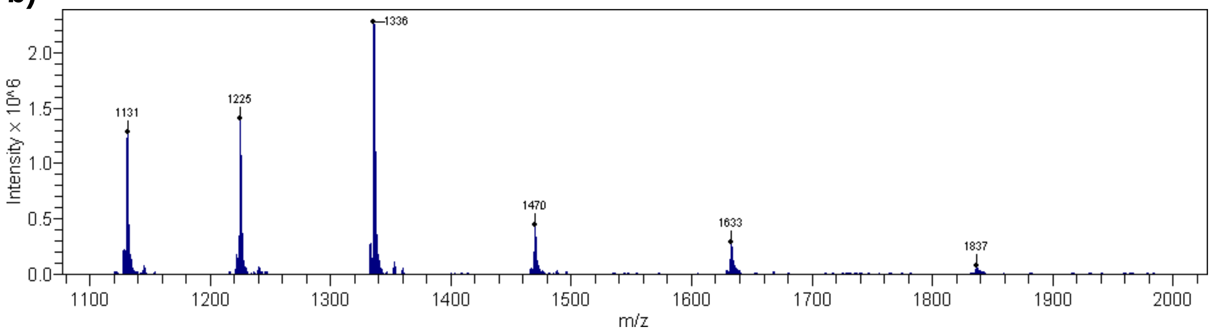

c)

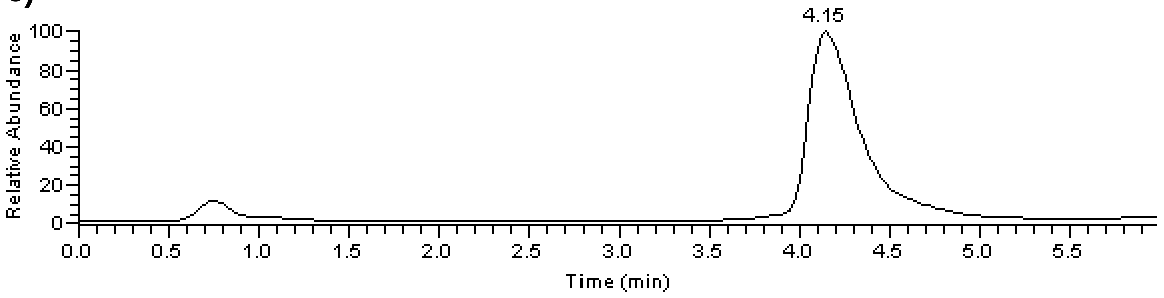

(a) deconvoluted, (b) non-deconvoluted, and (c) TIC mass spectrometry data for HER2DARPin(K68C) reacted with NMM.

Supplementary Figure 31. HER2DARPin(N69C) in PBS, 1 mM EDTA. Expected mass 14585.4 Da

Sequence

MRGSHHHHHHGSDLGKKLLEAARAGQDDEVRILMANGADVNAKDEYGLTPLYLATAHGHLEI
VEVLLKCGADVNAVDAIGFTPLHLAAFIGHLEIAEVLLKHGADVNAQDKFGKTAFDISIGNG
NEDLAEILQKLN

a)

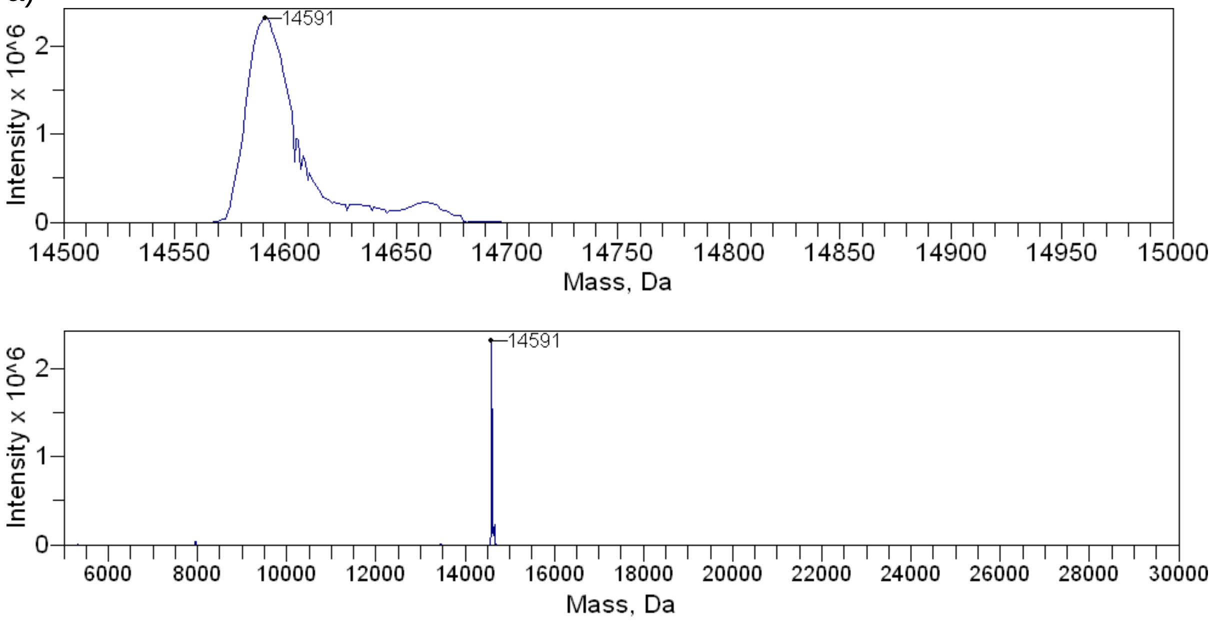

b)

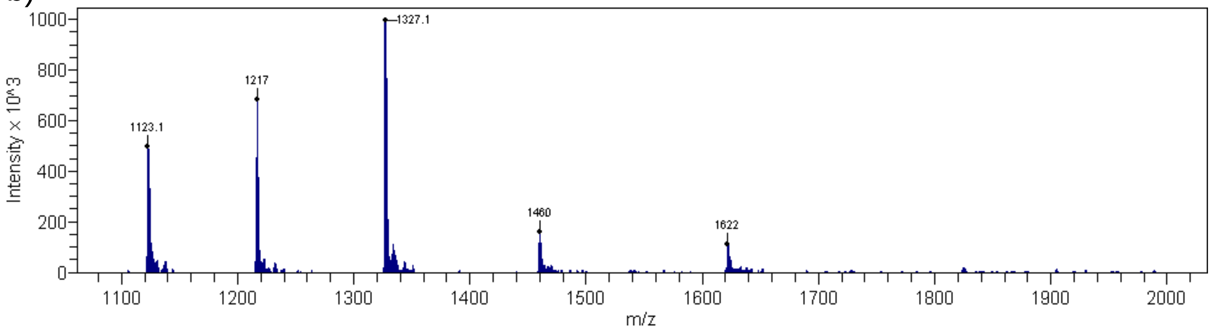

c)

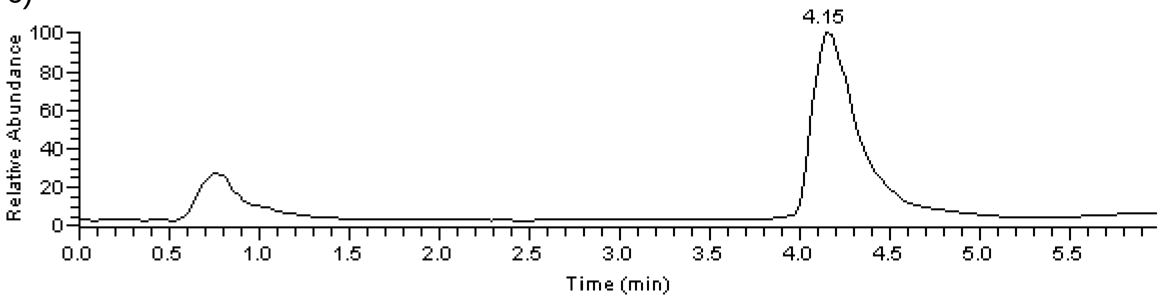

(a) deconvoluted, (b) non-deconvoluted, and (c) TIC mass spectrometry data for HER2DARPin(N69C).

Supplementary Figure 32. HER2DARPin(N69C) with 1 mM BrAcEGMe, 1 mM EDTA, in PBS pH 7.4, 10% DMF, 2 h, at 4 °C.
Expected mass (unmodified) 14585.4 Da
Expected mass (modified) 14700.6 Da

a)

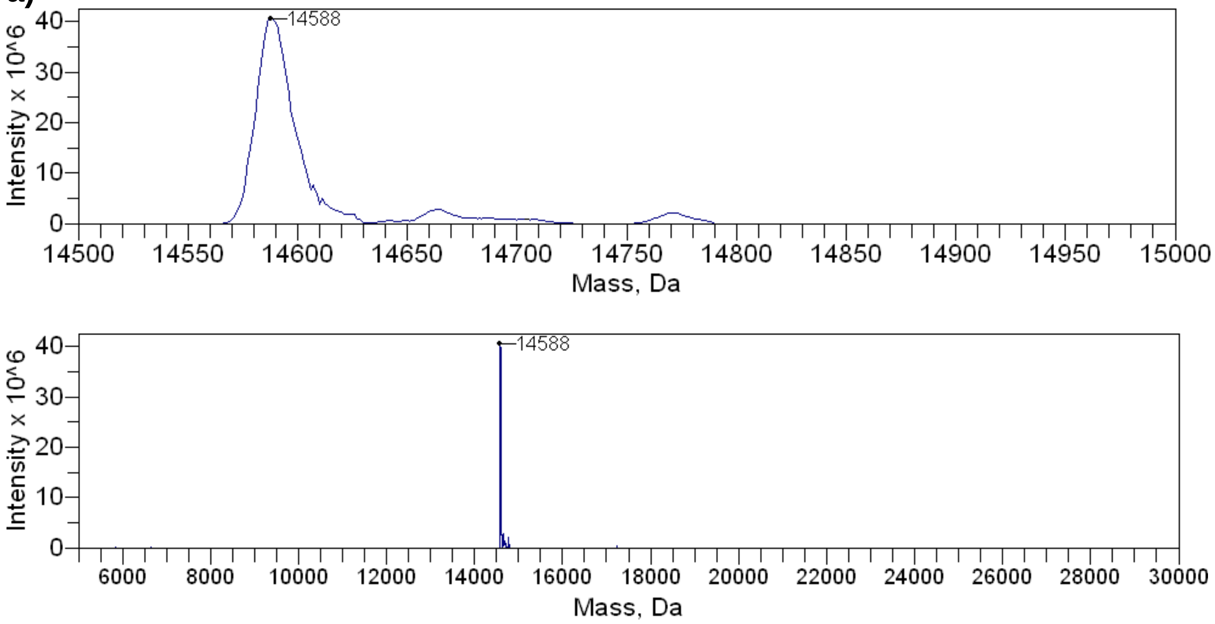

b)

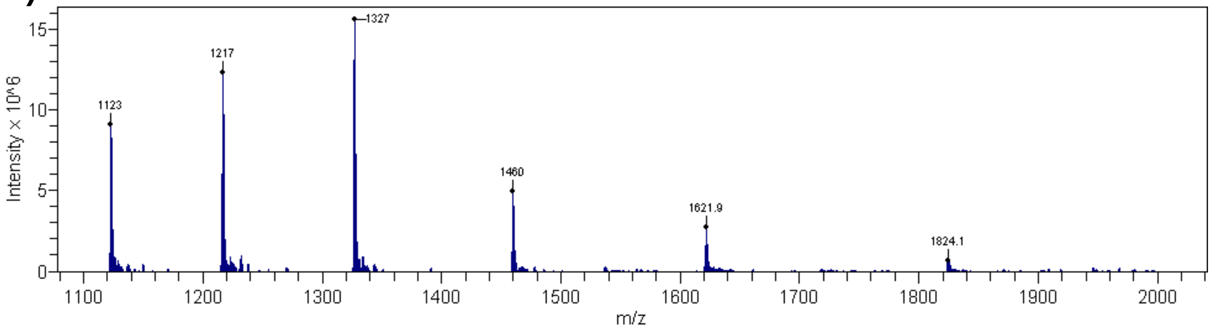

c)

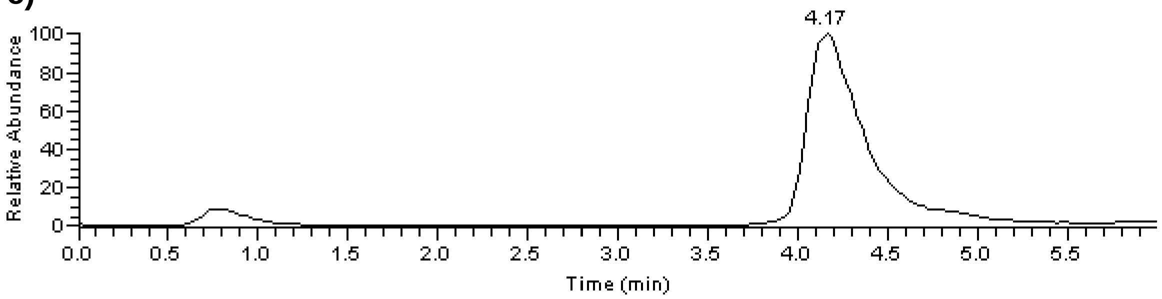

(a) deconvoluted, (b) non-deconvoluted, and (c) TIC mass spectrometry data for HER2DARPin(N69C) reacted with BrAcEGMe.

Supplementary Figure 33. HER2DARPin(N69C) with 1 mM NMM, 1 mM EDTA, in PBS pH 7.4, 10% DMF, 1 h, at 4 °C.
Expected mass (unmodified) 14585.4 Da
Expected mass (modified) 14696.5 Da

a)

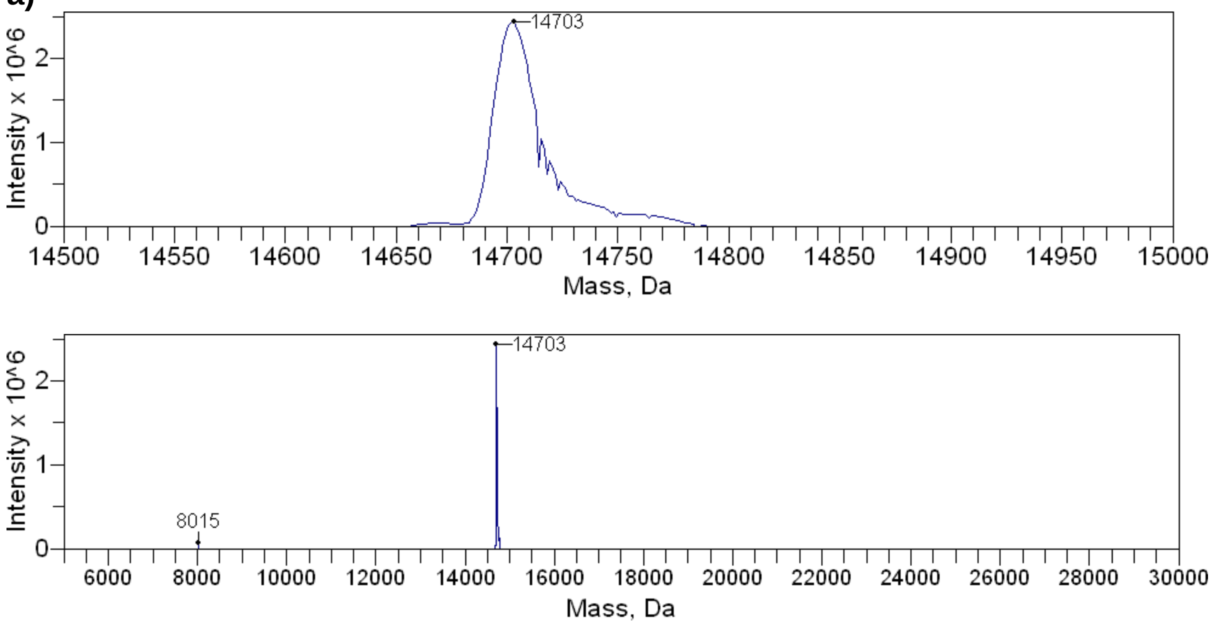

b)

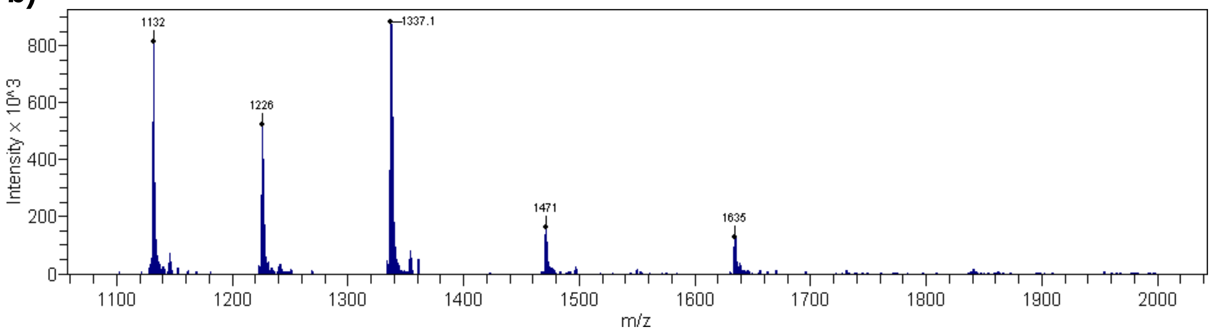

c)

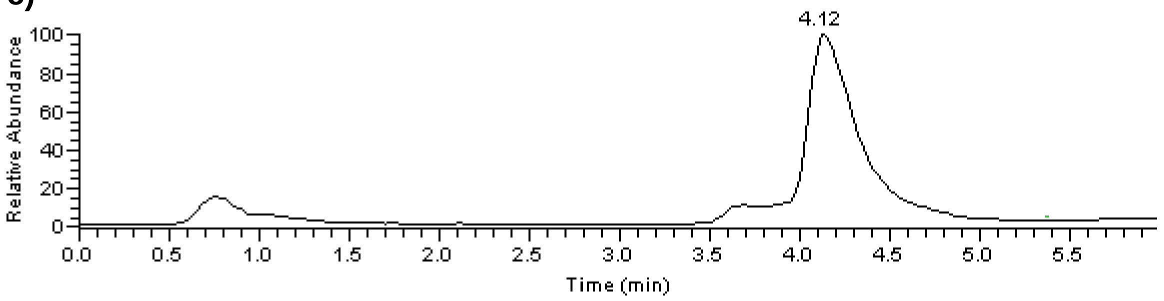

(a) deconvoluted, (b) non-deconvoluted, and (c) TIC mass spectrometry data for HER2DARPin(N69C) reacted with NMM.

Supplementary Figure 34. HER2DARPin(G70C) in PBS, 1 mM EDTA. Expected mass 14642.5 Da

Sequence

MRGSHHHHHHGSDLGKKLLEAARAGQDDEVRILMANGADVNAKDEYGLTPLYLATAHGHLEI
VEVLLKN**C**ADVNAVDAIGFTPLHLAAFIGHLEIAEVLLKHGADVNAQDKFGKTAFDISIGNG
NEDLAEILQKLN

a)

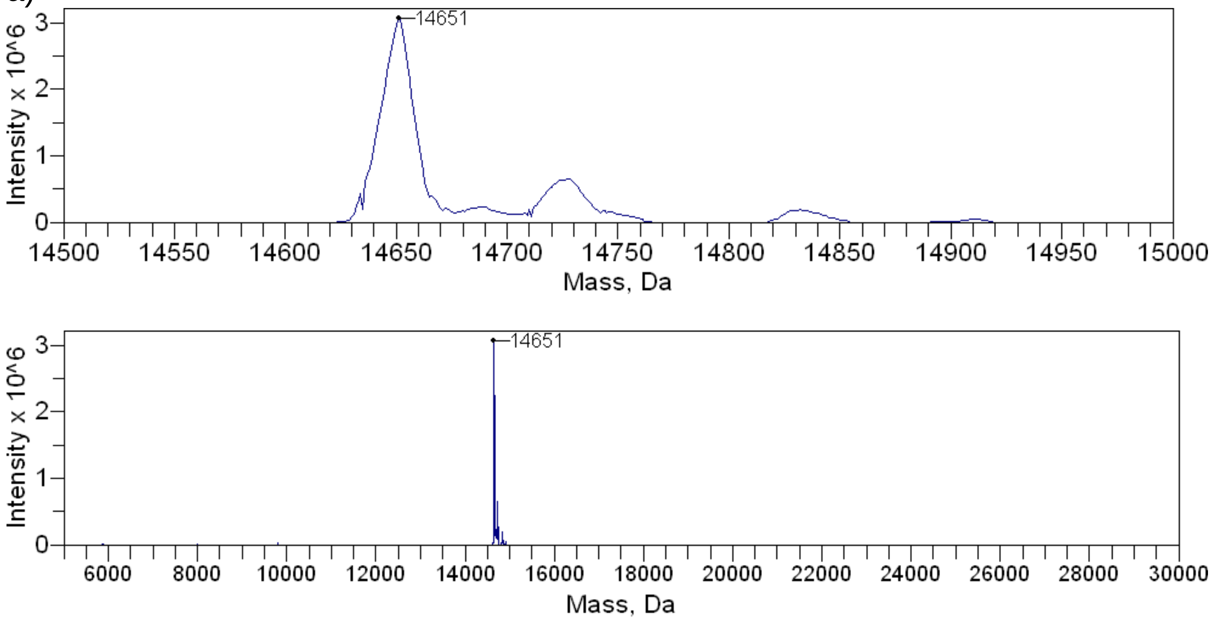

b)

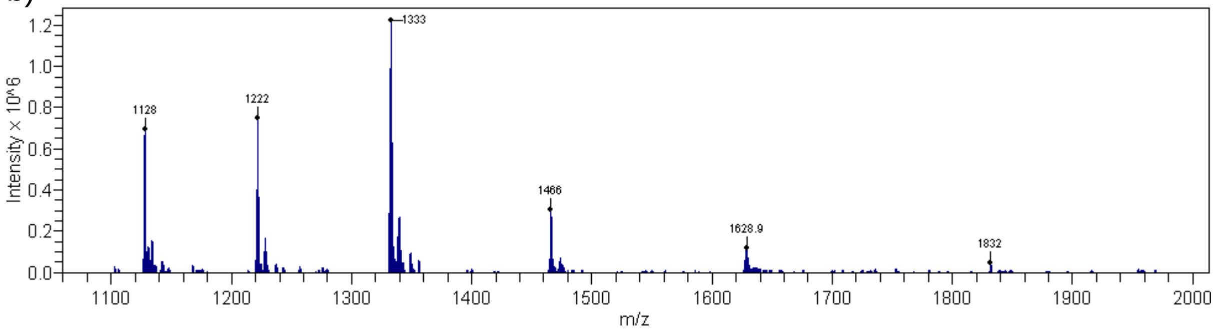

c)

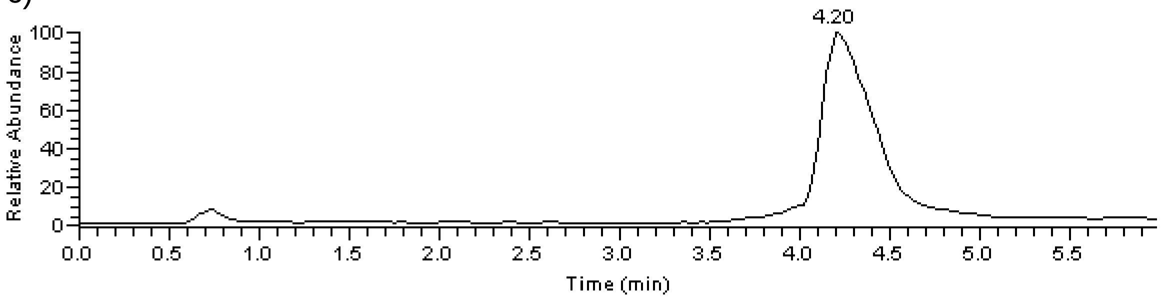

(a) deconvoluted, (b) non-deconvoluted, and (c) TIC mass spectrometry data for HER2DARPin(G70C).

Supplementary Figure 35. HER2DARPin(G70C) with 1 mM BrAcEGMe, 1 mM EDTA, in PBS pH 7.4, 10% DMF, 2 h, at 4 °C.
Expected mass (unmodified) 14642.4 Da
Expected mass (modified) 14757.6 Da

a)

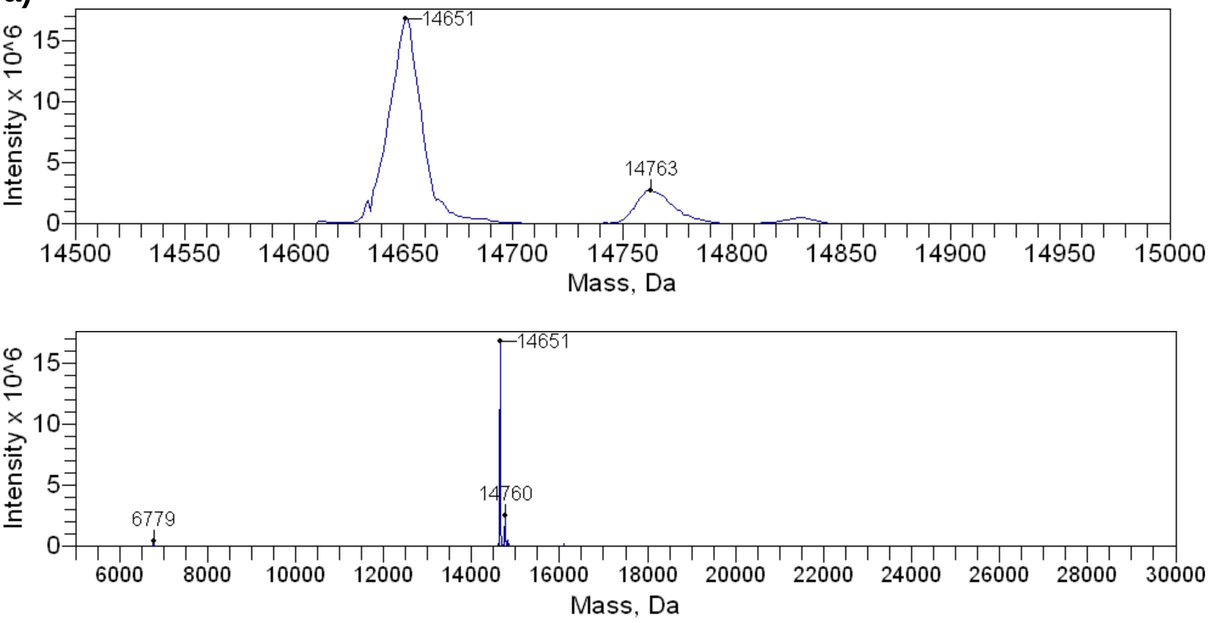

b)

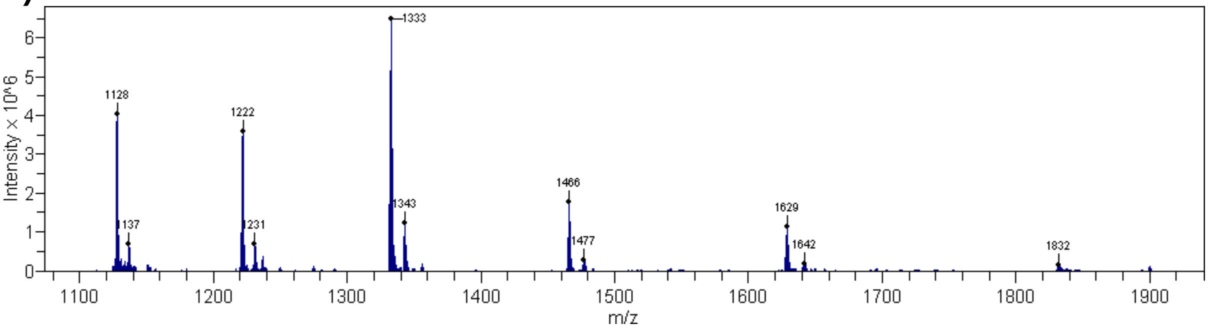

c)

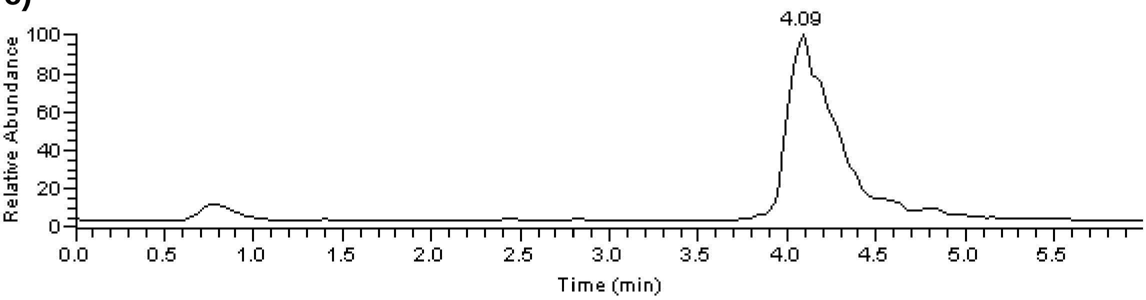

(a) deconvoluted, (b) non-deconvoluted, and (c) TIC mass spectrometry data for HER2DARPin(G70C) reacted with BrAcEGMe.

Supplementary Figure 36. HER2DARPin(G70C) with 1 mM NMM, in PBS, 1 mM EDTA, pH 7.4, 10% DMF, 1 h, at 4 °C.
Expected mass (unmodified) 14642.4 Da
Expected mass (modified) 14753.5 Da

a)

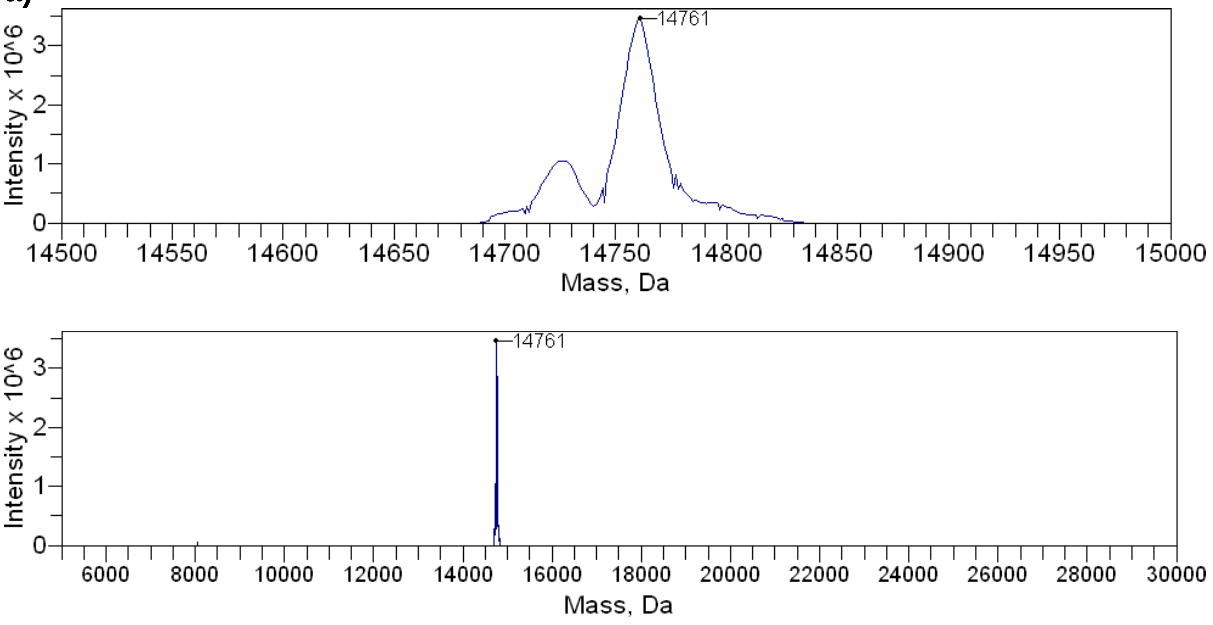

b)

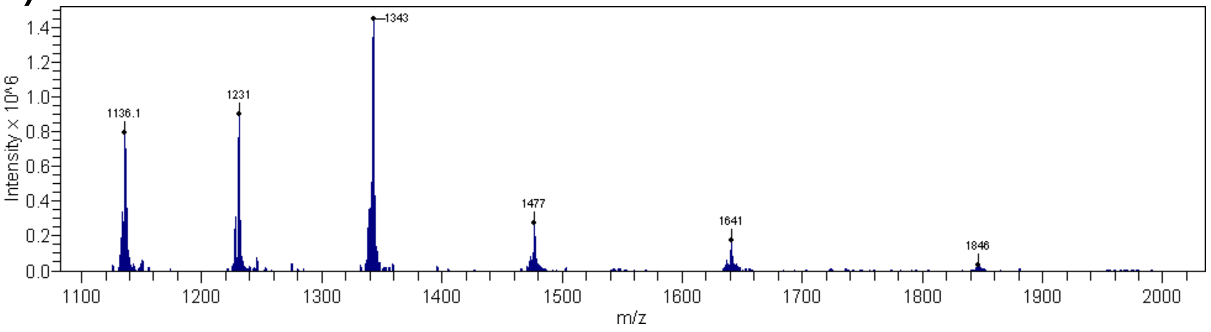

c)

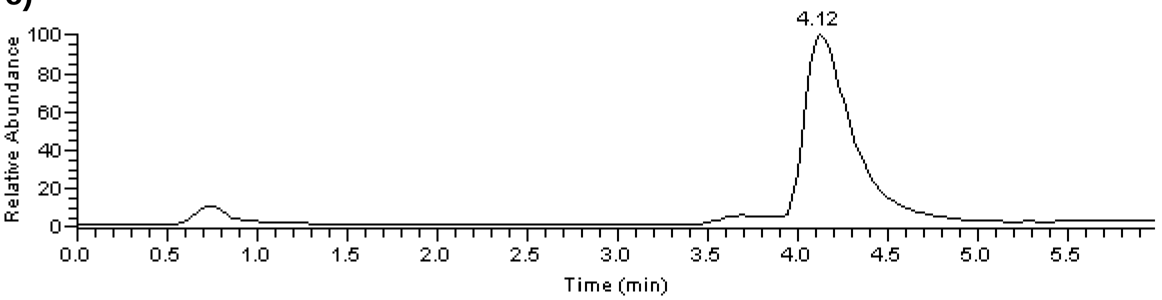

(a) deconvoluted, (b) non-deconvoluted, and (c) TIC mass spectrometry data for HER2DARPin(G70C) reacted with NMM.

Supplementary Figure 37. HER2DARPin(D72C) in PBS, 1 mM EDTA. Expected mass 14584.4 Da

Sequence

MRGSHHHHHHGSDLGKKLLEAARAGQDDEVRILMANGADVNAKDEYGLTPLYLATAHGHLEI
VEVLLKNGACVNAVDAIGFTPLHLAAFIGHLEIAEVLLKHGADVNAQDKFGKTAFDISIGNG
NEDLAEILQKLN

a)

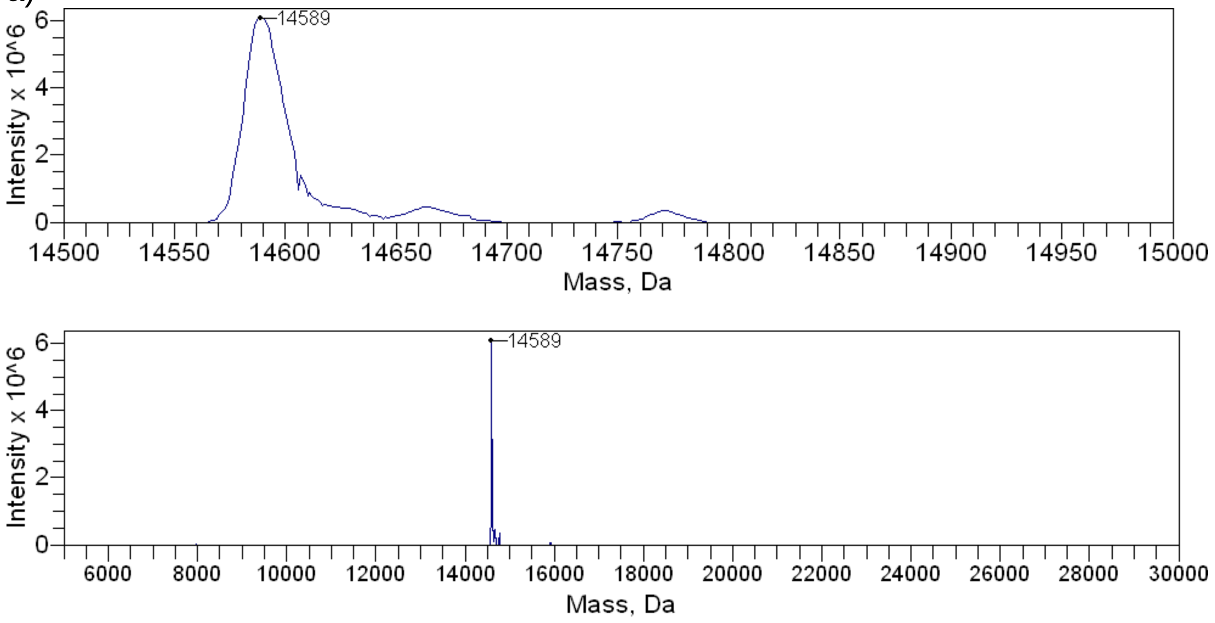

b)

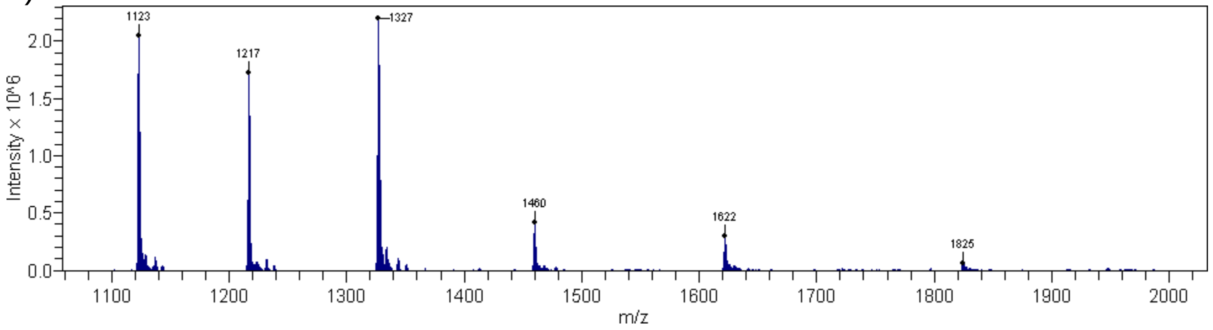

c)

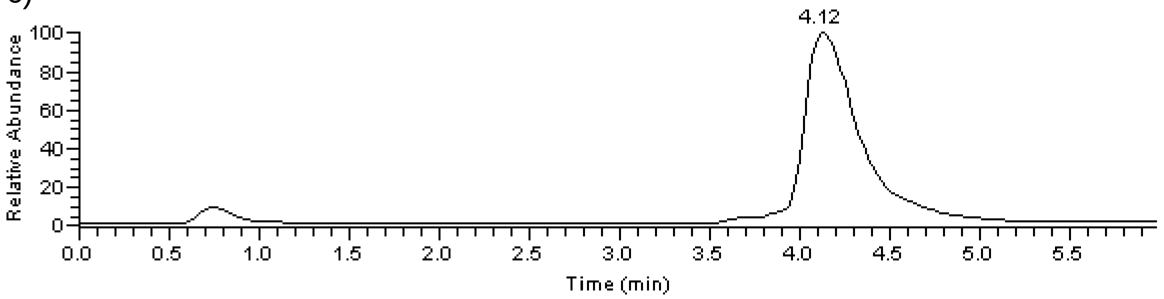

(a) deconvoluted, (b) non-deconvoluted, and (c) TIC mass spectrometry data for HER2DARPin(D72C).

Supplementary Figure 38. HER2DARPin(D72C) with 1 mM BrAcEGMe, 1 mM EDTA, in PBS pH 7.4, 10% DMF, 2 h, at 4 °C.
Expected mass (unmodified)   14584.4 Da
Expected mass (modified)   14699.6 Da

a)

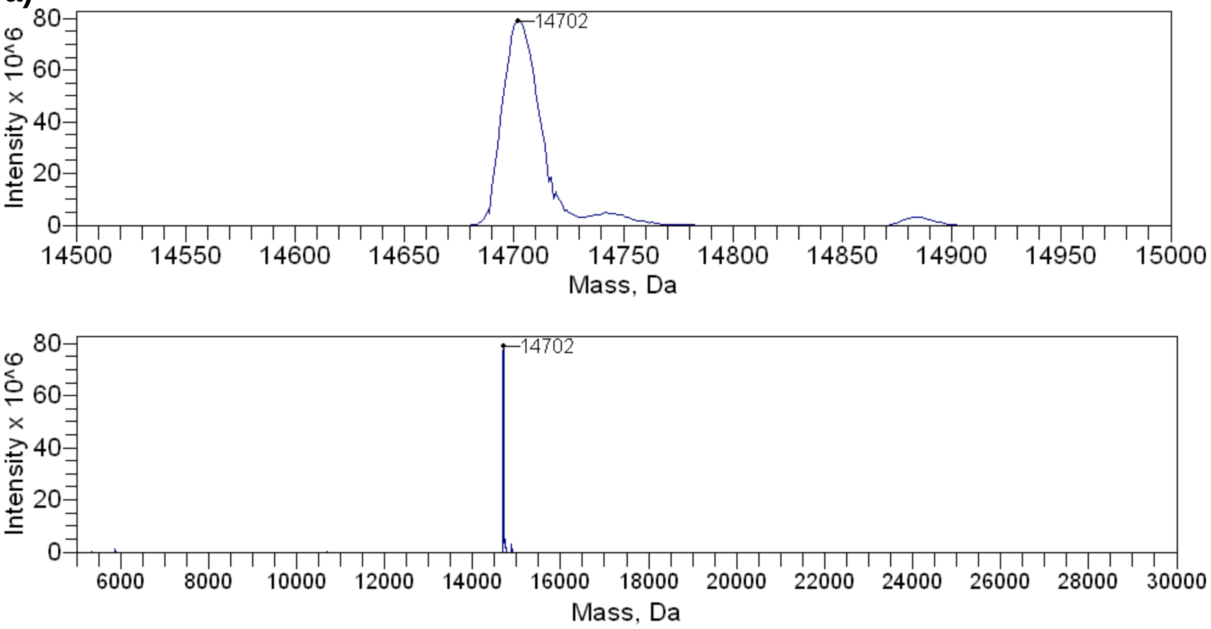

b)

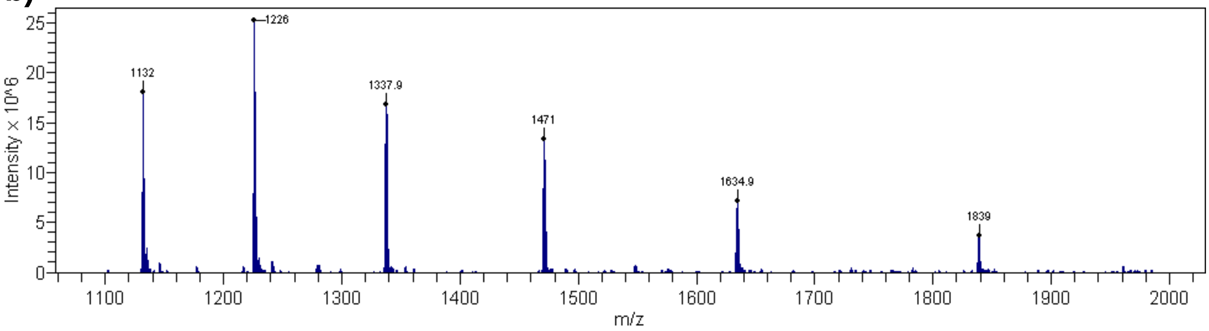

c)

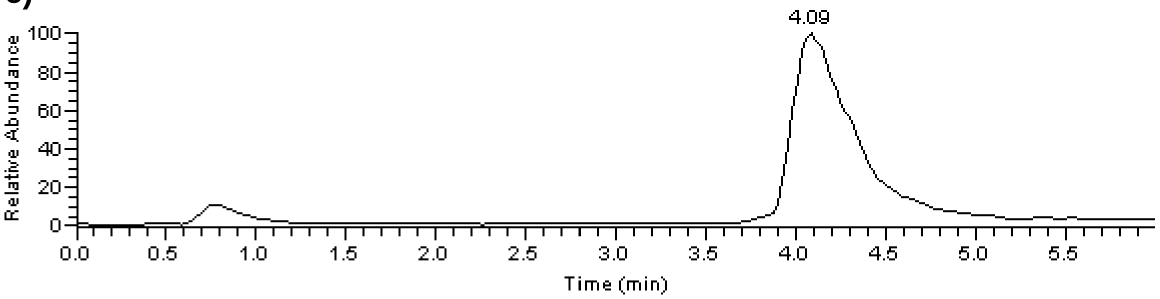

(a) deconvoluted, (b) non-deconvoluted, and (c) TIC mass spectrometry data for HER2DARPin(D72C) reacted with BrAcEGMe.

Supplementary Figure 39. HER2DARPin(D72C) with 1 mM NMM, 1 mM EDTA, in PBS pH 7.4, 10% DMF, 1 h, at 4 °C.
Expected mass (unmodified) 14584.4 Da
Expected mass (modified) 14695.5 Da

a)

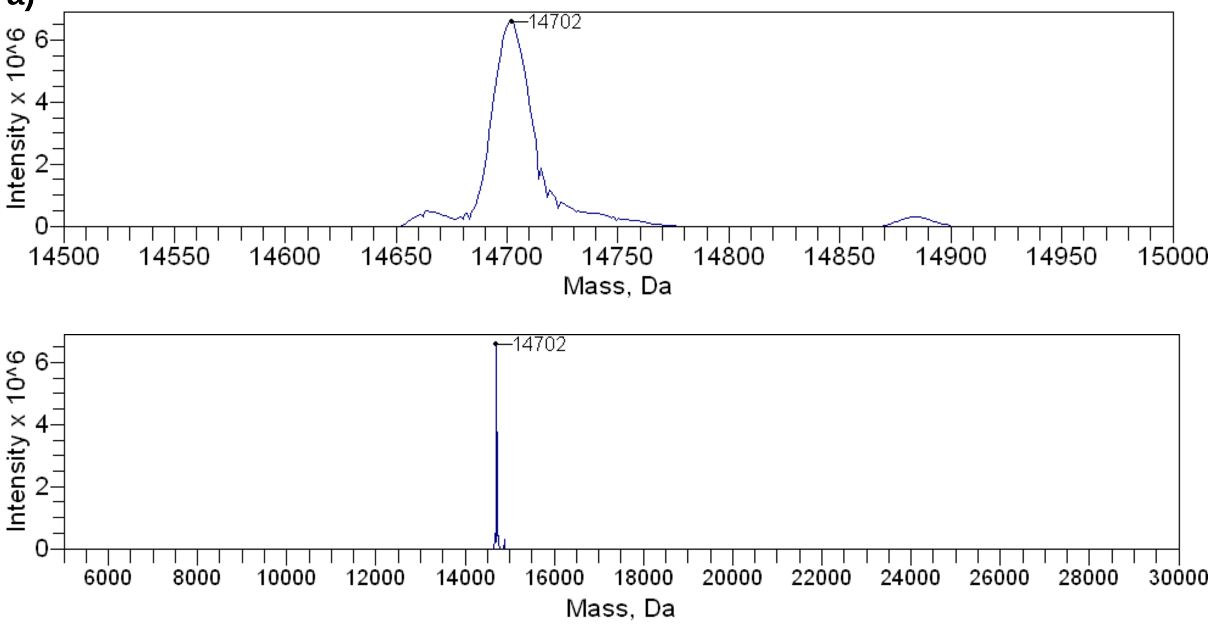

b)

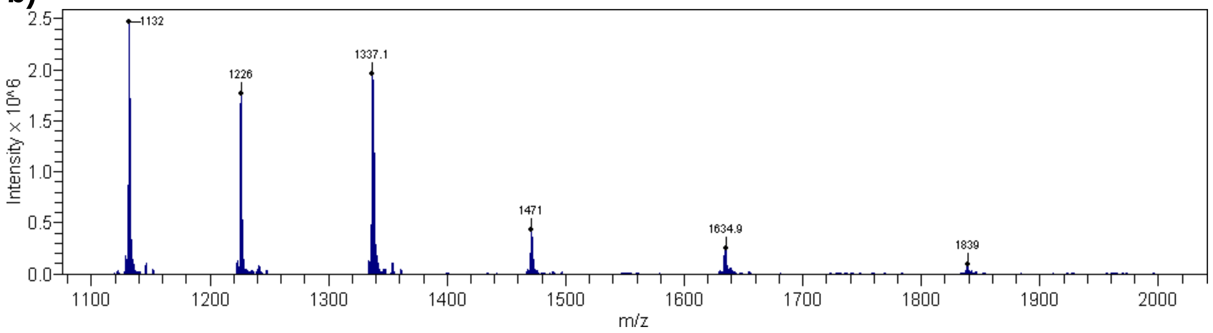

c)

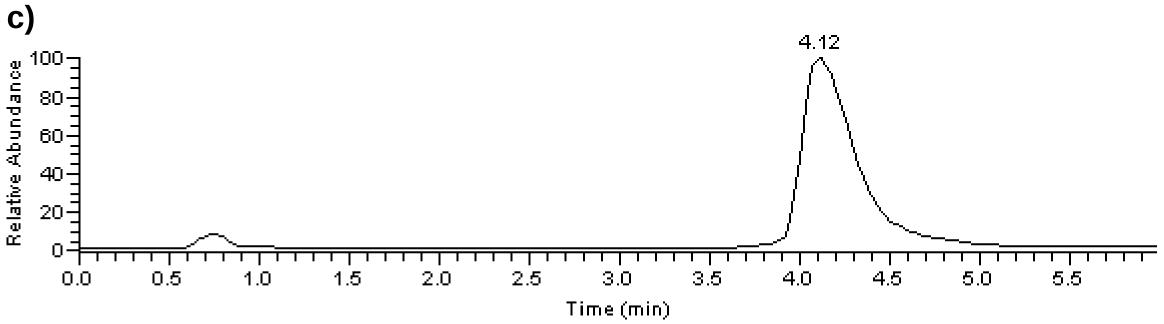

(a) deconvoluted, (b) non-deconvoluted, and (c) TIC mass spectrometry data for HER2DARPin(D72C) reacted with NMM.

Supplementary Figure 40. HER2DARPin(H102C) in PBS, 1 mM EDTA – Expected mass 14562.4 Da

Sequence

MRGSHHHHHHGSDLGKKLLEAARAGQDDEVRILMANGADVNAKDEYGLTPLYLATAHGHLEI
VEVLLKNGADVNAVDAIGFTPLHLAAFIGHLEIAEVLLKCGADVNAQDKFGKTAFDISIGNG
NEDLAEILQKLN

a)

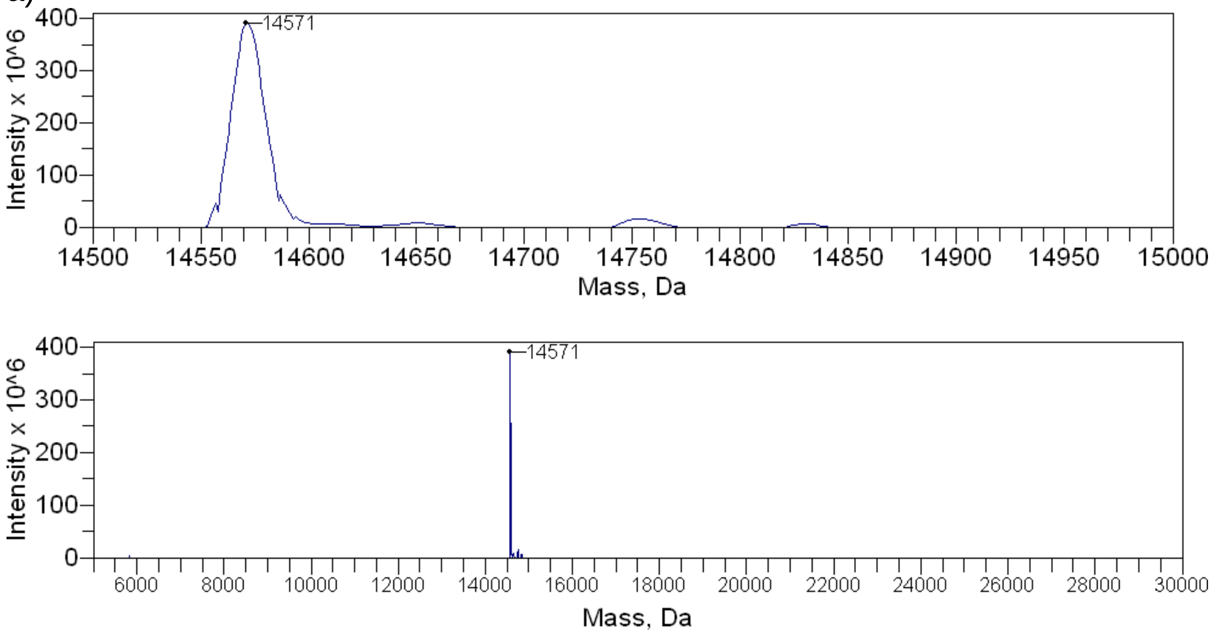

b)

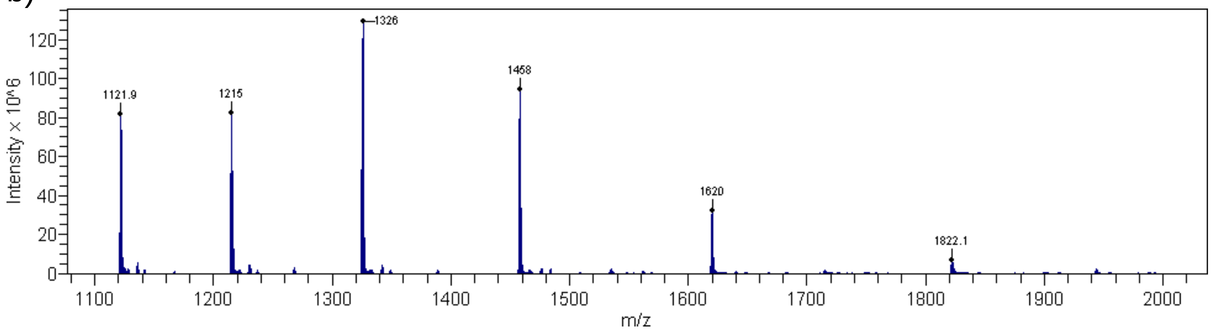

c)

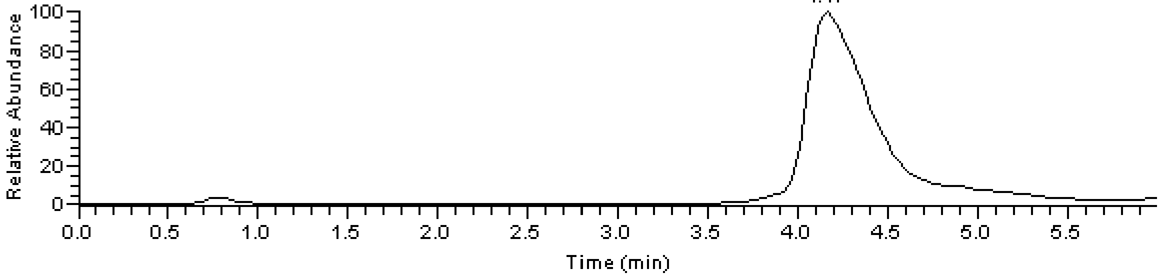

(a) deconvoluted, (b) non-deconvoluted, and (c) TIC mass spectrometry data for HER2DARPin(H102C).

Supplementary Figure 41. HER2DARPin(H102C) with 1 mM BrAcEGMe, 1 mM EDTA, in PBS pH 7.4, 10% DMF, 2 h, at 4 °C.
Expected mass (unmodified) 14562.4 Da
Expected mass (modified) 14677.6 Da

a)

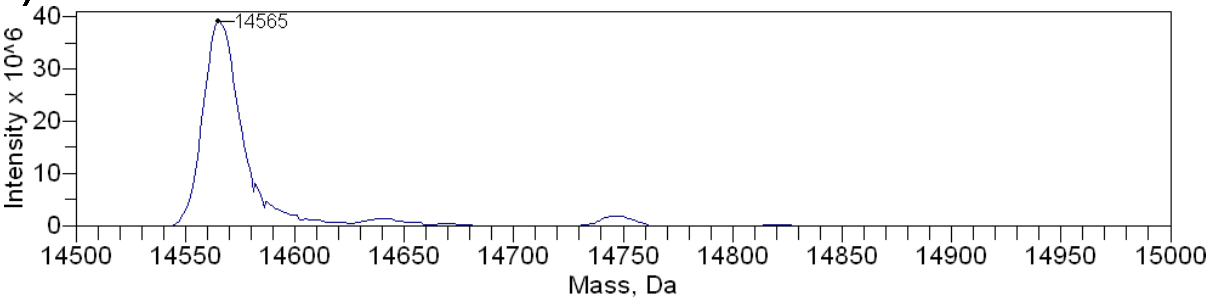

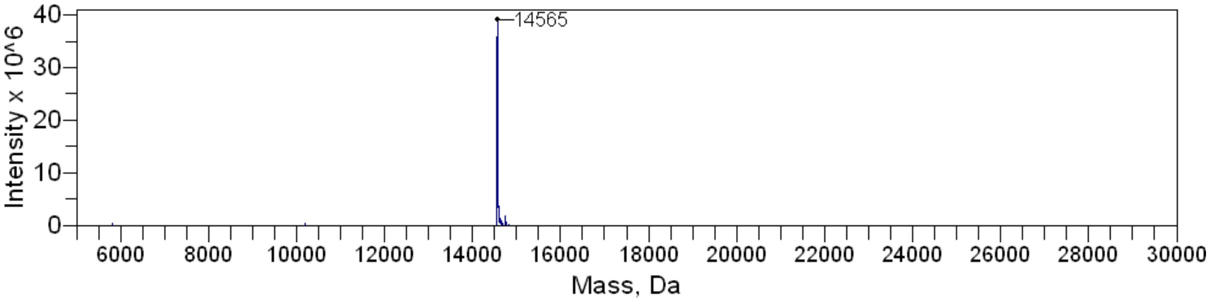

b)

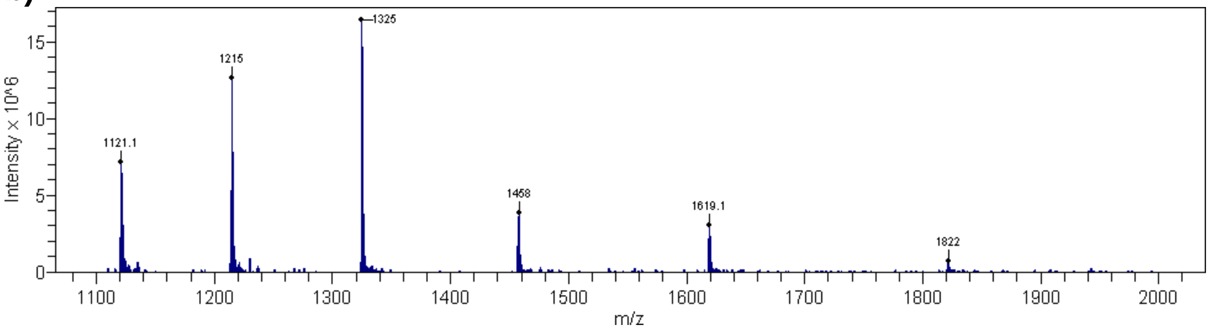

c)

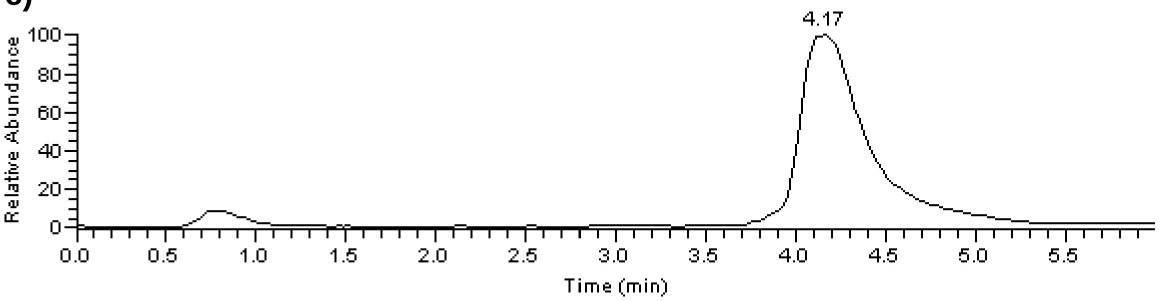

(a) deconvoluted, (b) non-deconvoluted, and (c) TIC mass spectrometry data for HER2DARPin(H102C) reacted with BrAcEGMe.

Supplementary Figure 42. HER2DARPin(H102C) with 1 mM NMM, 1 mM EDTA, in PBS pH 7.4, 10% DMF, 1 h, at 4 °C.
Expected mass (unmodified) 14562.4 Da
Expected mass (modified) 14673.5 Da

a)

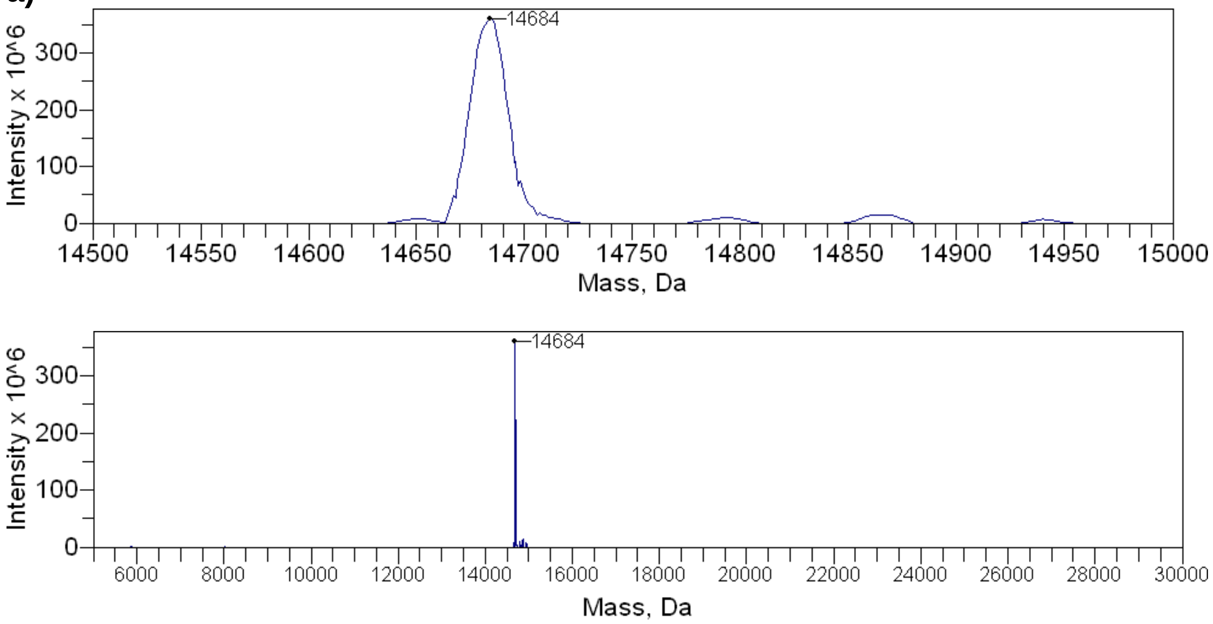

b)

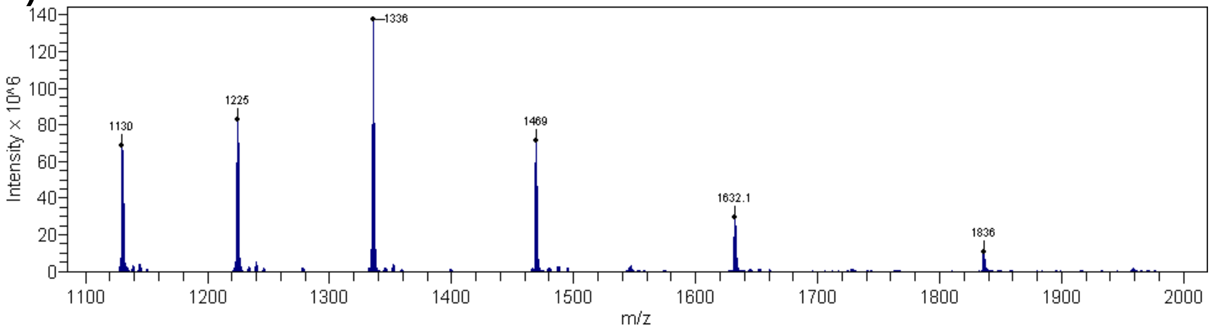

c)

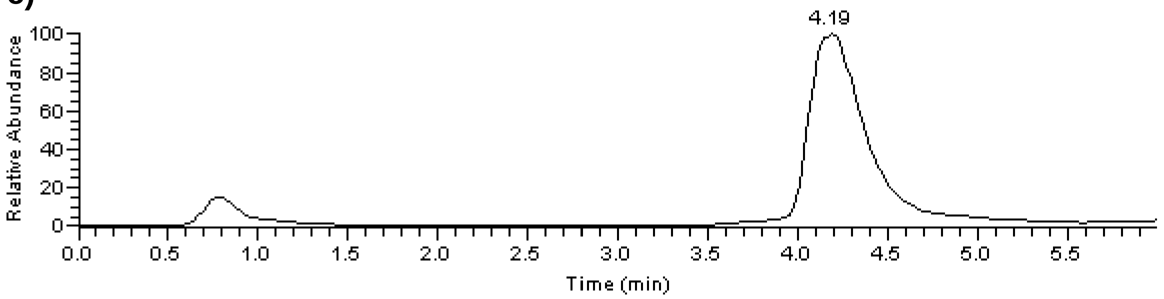

(a) deconvoluted, (b) non-deconvoluted, and (c) TIC mass spectrometry data for HER2DARPin(H102C) reacted with NMM.

Supplementary Figure 43. HER2DARPin(D105C) in PBS, 1 mM EDTA. Expected mass 14584.4 Da

Sequence
MRGSHHHHHHGSDLGKKLLEAARAGQDDEVRILMANGADVNAKDEYGLTPLYLATAHGHLEI
VEVLLKNGADVNAVDAIGFTPLHLAAFIGHLEIAEVLLKHGACVNAQDKFGKTAFDISIGNG
NEDLAEILQKLN

a)

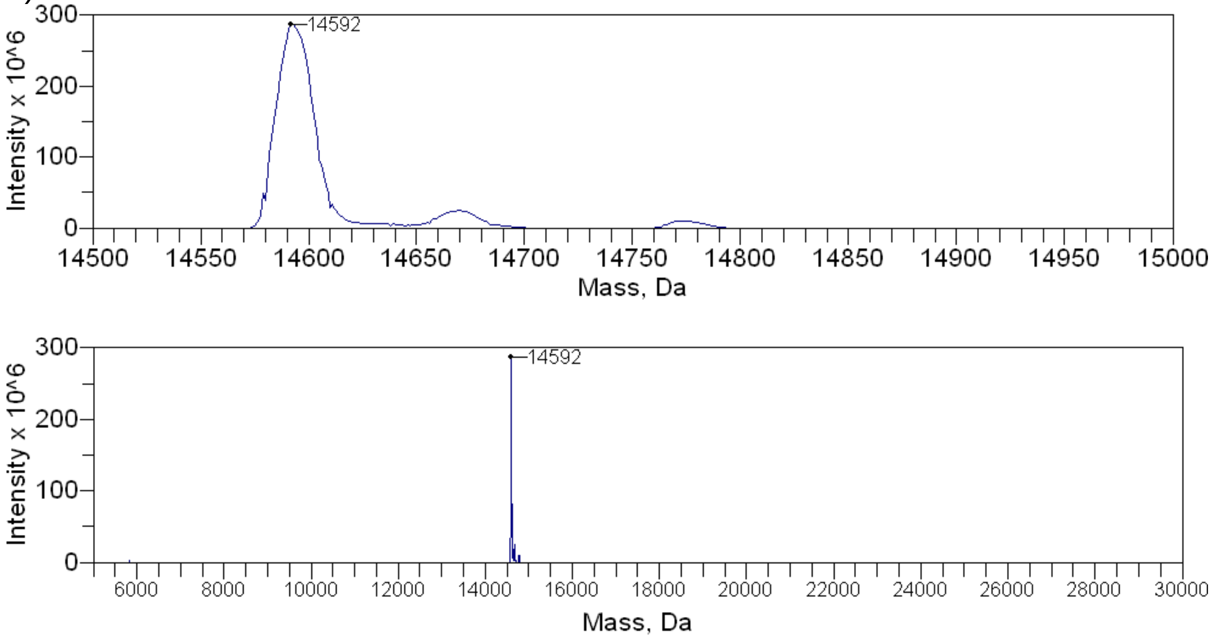

b)

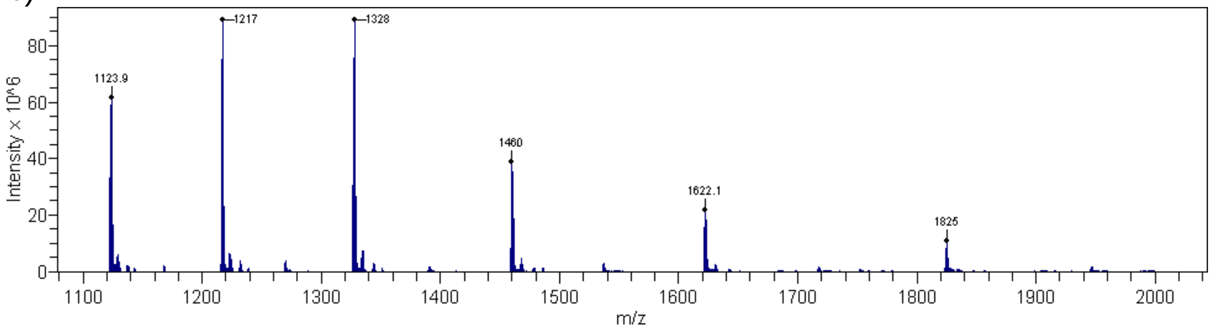

c)

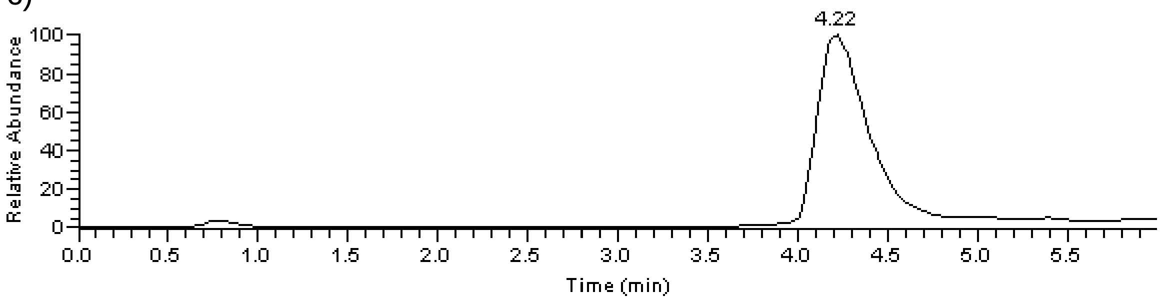

(a) deconvoluted, (b) non-deconvoluted, and (c) TIC mass spectrometry data for HER2DARPin(D105C).

Supplementary Figure 44. HER2DARPin(D105C) with 1 mM BrAcEGMe, 1 mM EDTA, in PBS pH 7.4, 10% DMF, 2 h, at 4 °C.
**Expected mass (unmodified)	14584.4 Da**
**Expected mass (modified)	14699.6 Da**

a)

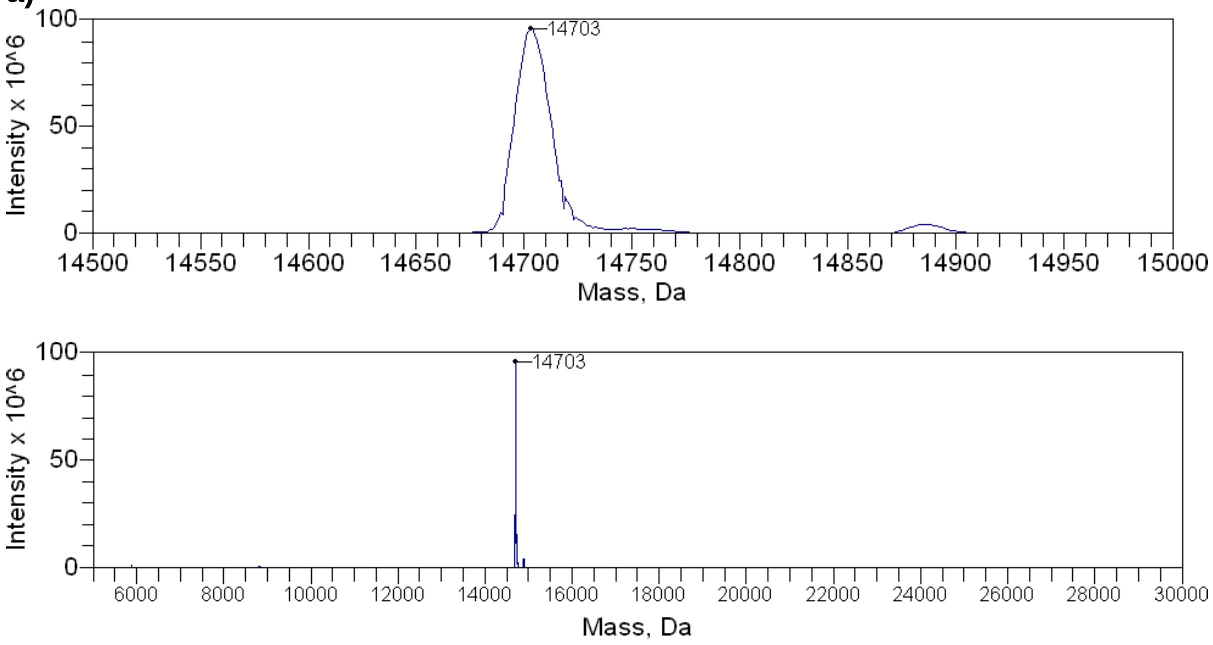

b)

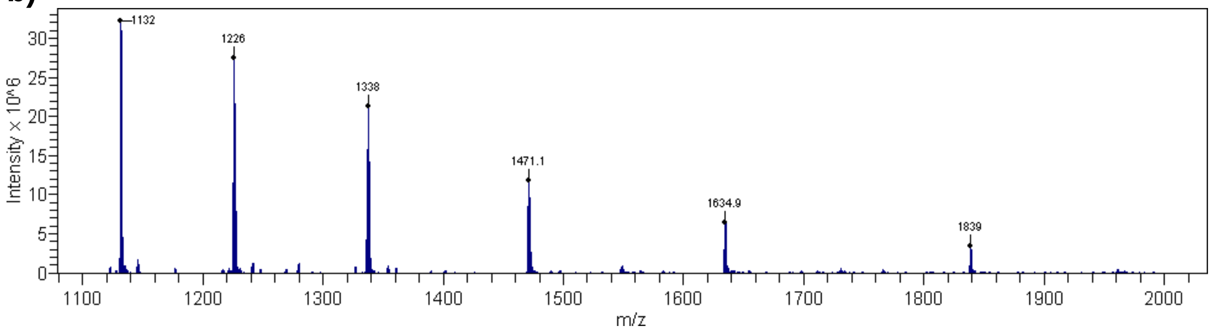

c)

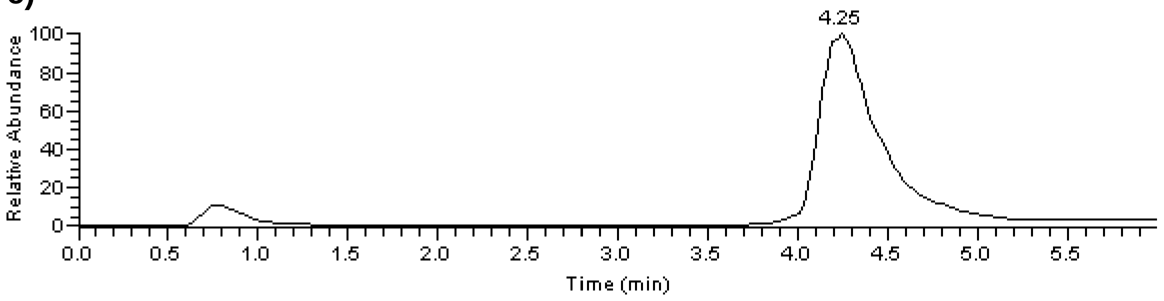

(a) deconvoluted, (b) non-deconvoluted, and (c) TIC mass spectrometry data for HER2DARPin(D105C) reacted with BrAcEGMe.

Supplementary Figure 45. HER2DARPin(D105C) with 1 mM NMM, 1 mM EDTA, in PBS pH 7.4, 10% DMF, 1 h, at 4 °C.
Expected mass (unmodified) 14584.4 Da
Expected mass (modified) 14695.5 Da

a)

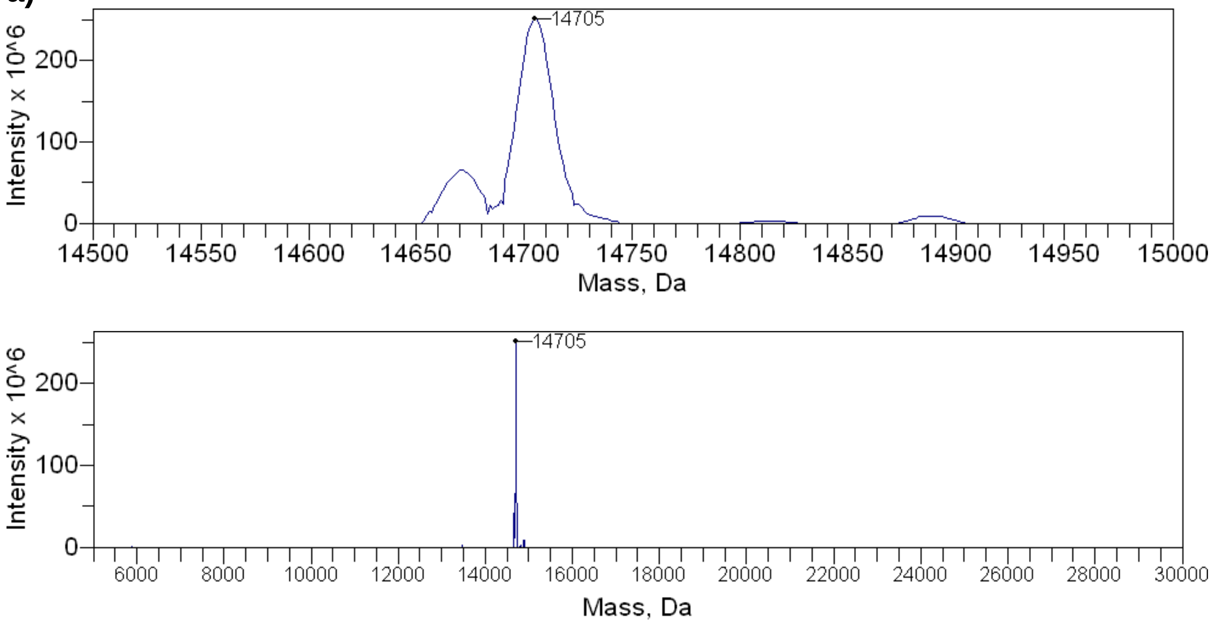

b)

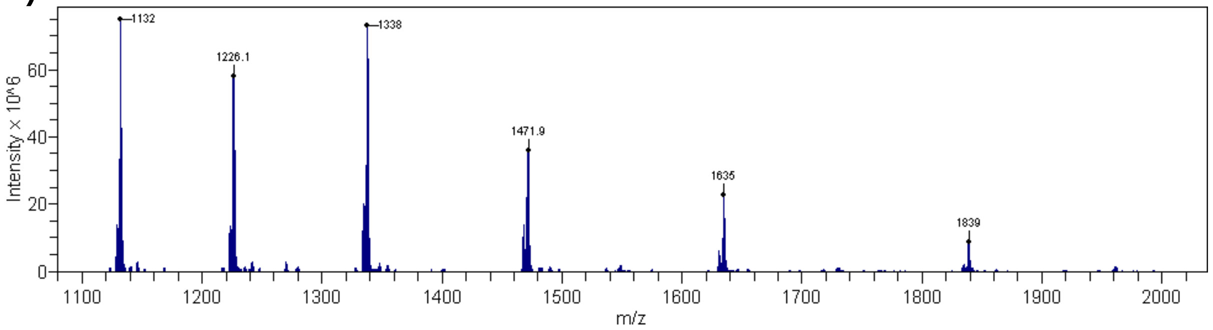

c)

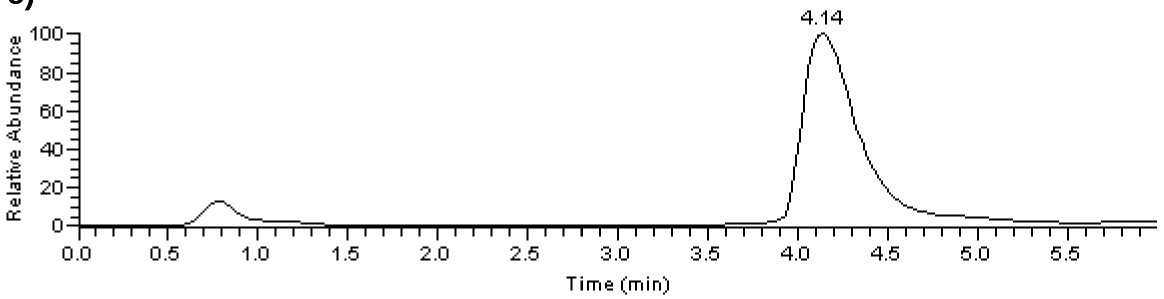

(a) deconvoluted, (b) non-deconvoluted, and (c) TIC mass spectrometry data for HER2DARPin(D105C) reacted with NMM.

Supplementary Figure 46. HER2DARPin(L135C) in PBS, 1 mM EDTA. Expected mass 14586.4 Da

Sequence

MRGSHHHHHHGSDLGKKLLEAARAGQDDEVRILMANGADVNAKDEYGLTPLYLATAHGHLEI
VEVLLKNGADVNAVDAIGFTPLHLAAFIGHLEIAEVLLKHGADVNAQDKFGKTAFDISIGNG
NEDLAEILQK**C**N

a)

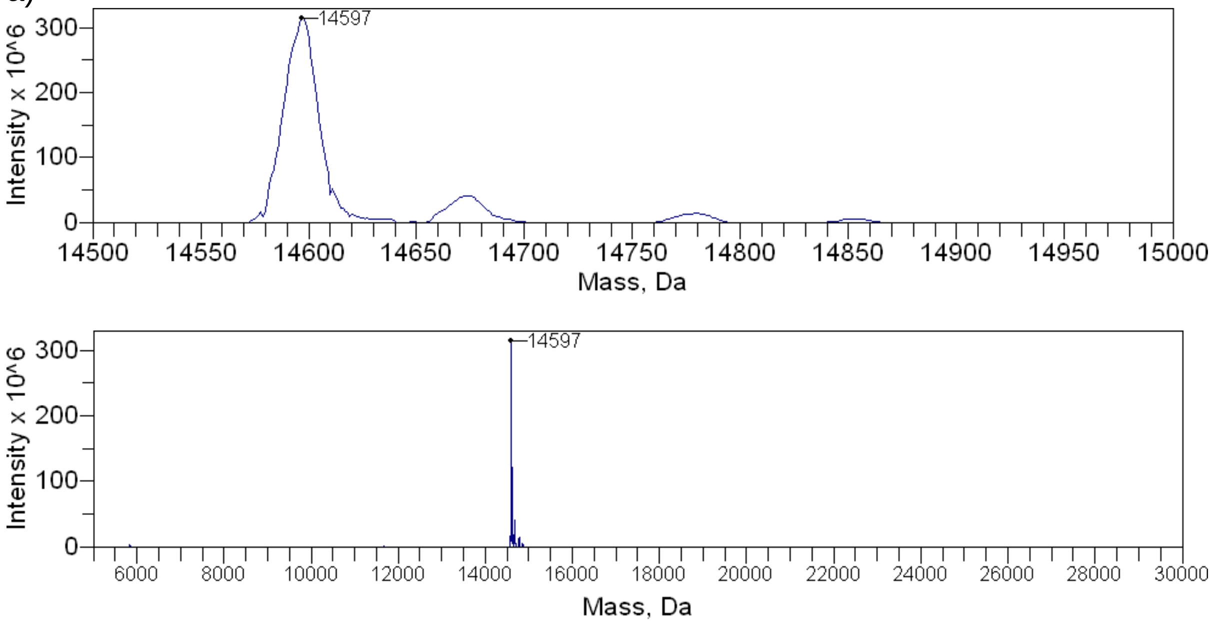

b)

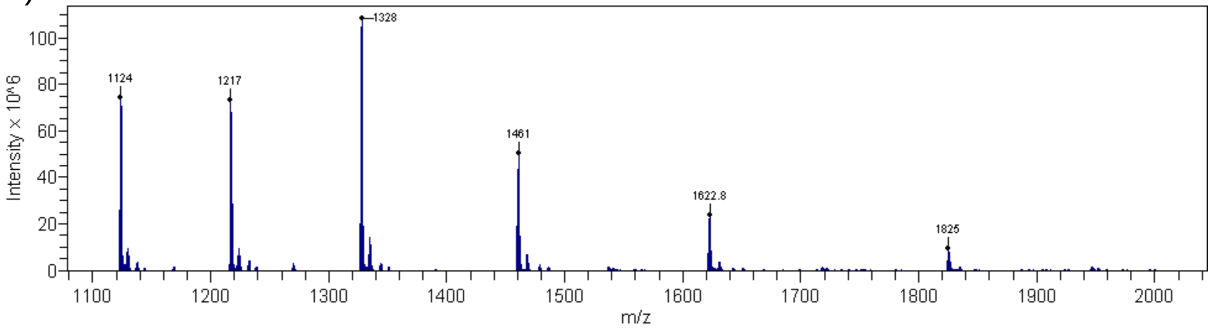

c)

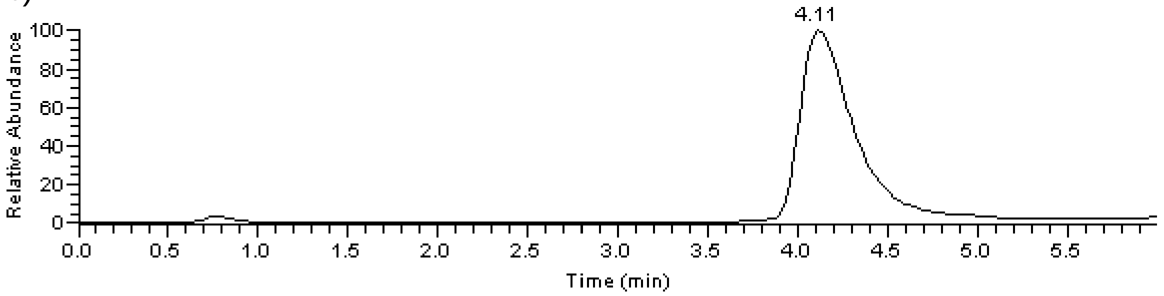

(a) deconvoluted, (b) non-deconvoluted, and (c) TIC mass spectrometry data for HER2DARPin(L135C).

Supplementary Figure 47. HER2DARPin(L135C) with 1 mM BrAcEGMe, 1 mM EDTA, in PBS pH 7.4, 10% DMF, 2 h, at 4 °C.
Expected mass (unmodified) 14586.4 Da
Expected mass (modified) 14701.6 Da

a)

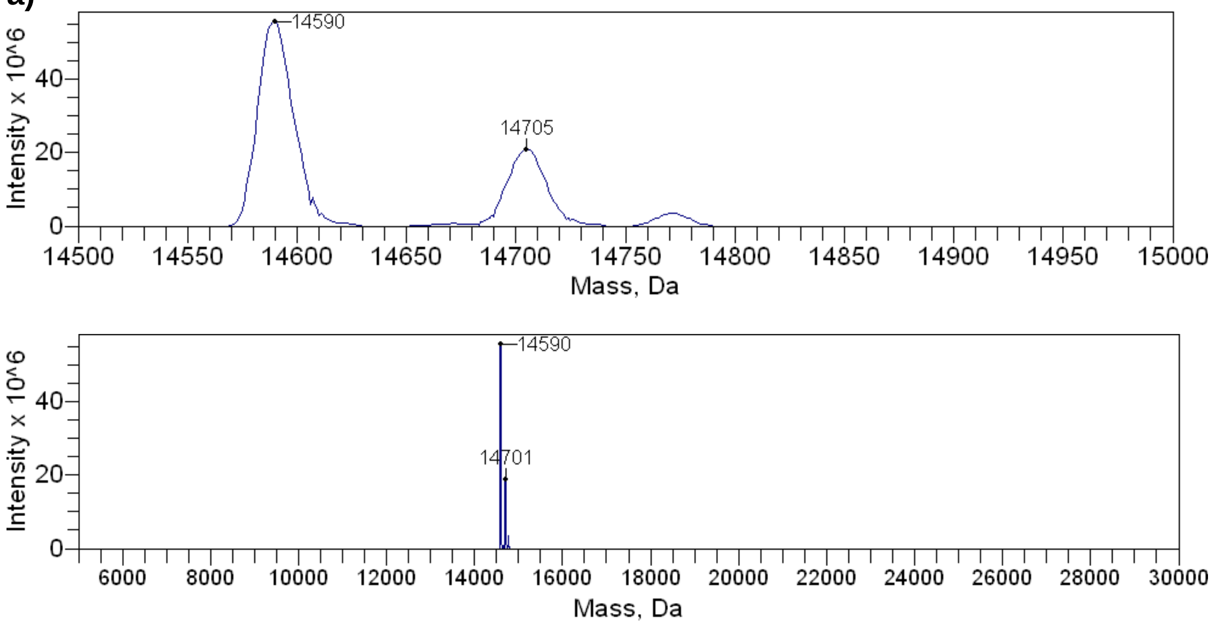

b)

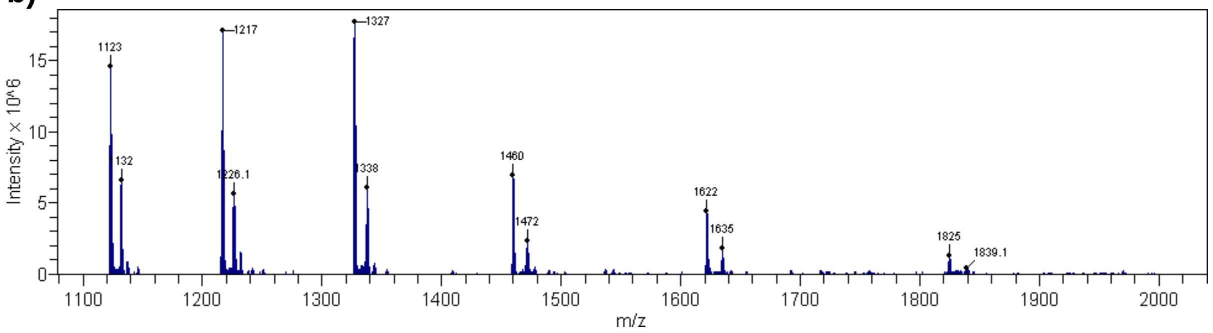

c)

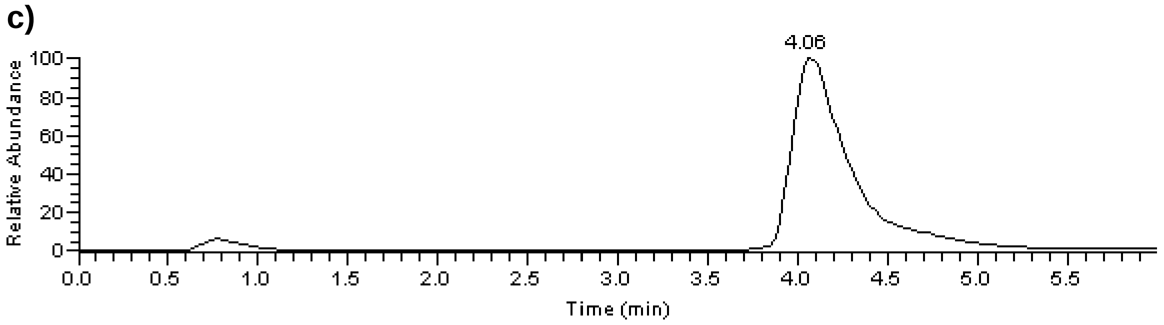

(a) deconvoluted, (b) non-deconvoluted, and (c) TIC mass spectrometry data for HER2DARPin(L135C) reacted with BrAcEGMe.

Supplementary Figure 48. HER2DARPin(L135C) with 1 mM NMM, 1 mM EDTA, in PBS pH 7.4, 10% DMF, 1 h, at 4 °C.
Expected mass (unmodified) 14586.4 Da
Expected mass (modified) 14697.5 Da

a)

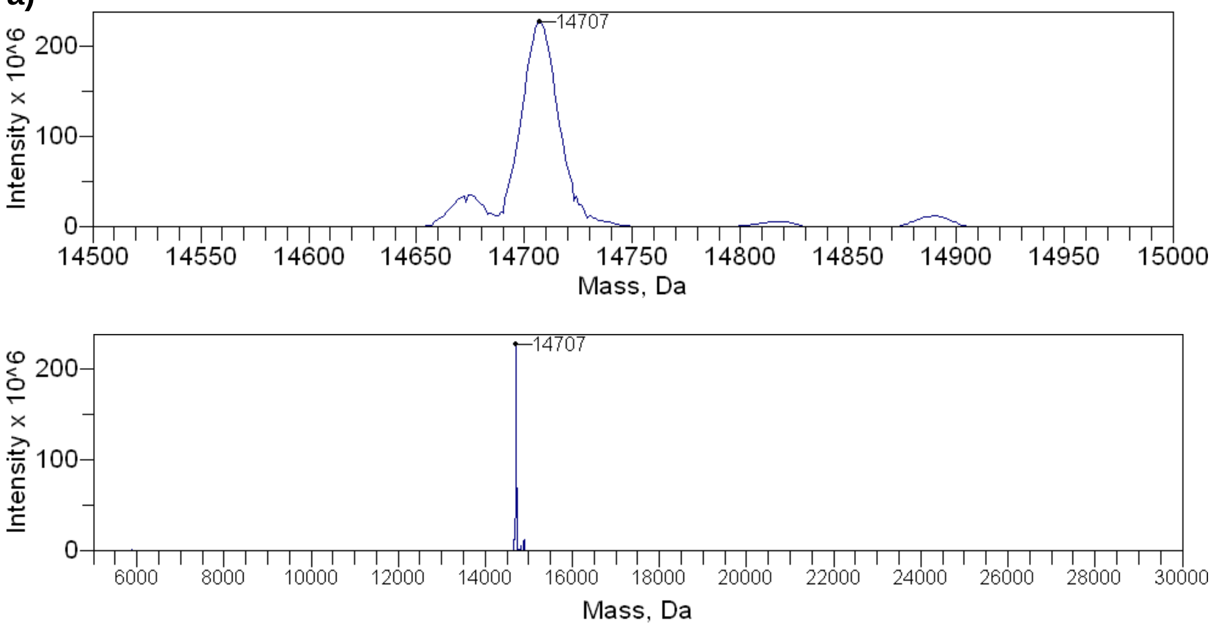

b)

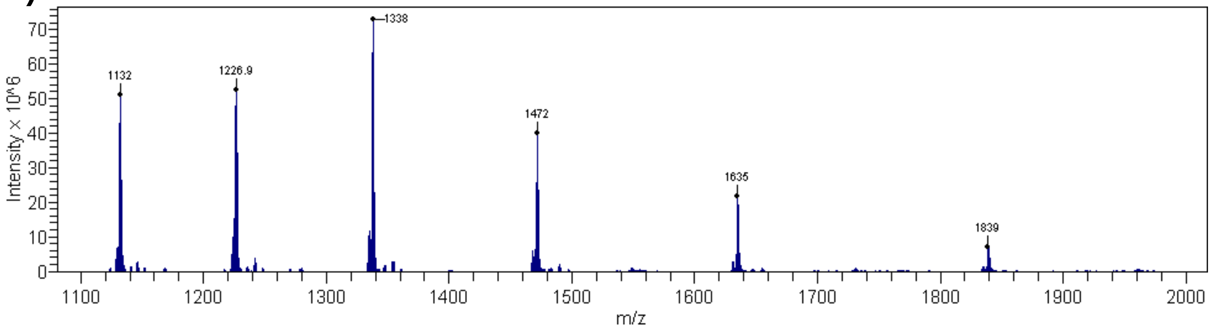

c)

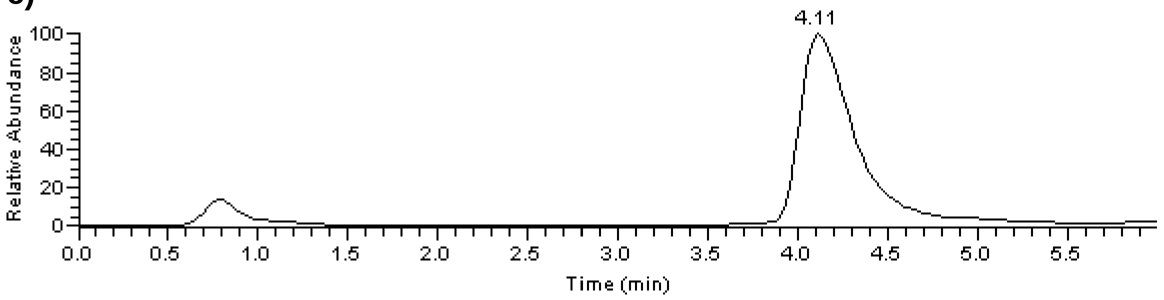

(a) deconvoluted, (b) non-deconvoluted, and (c) TIC mass spectrometry data for HER2DARPin(L135C) reacted with NMM.

Supplementary Figure 49. HER2DARPin(Cmut, L135C) in PBS, 1 mM EDTA. Expected mass 14676.4 Da

Sequence

MRGSHHHHHHGSDLGKKLLEAARAGQDDEVRILMANGADVNAKDEYGLTPLYLATAHGHLEI
VEVLLKNGADVNAVDAIGFTPLHLAAFIGHLEIAEVLLKHGADVNAQDKFGKT**P**FD**LA**I**RE**G
HED**I**AE**V**LQK**C**A

a)

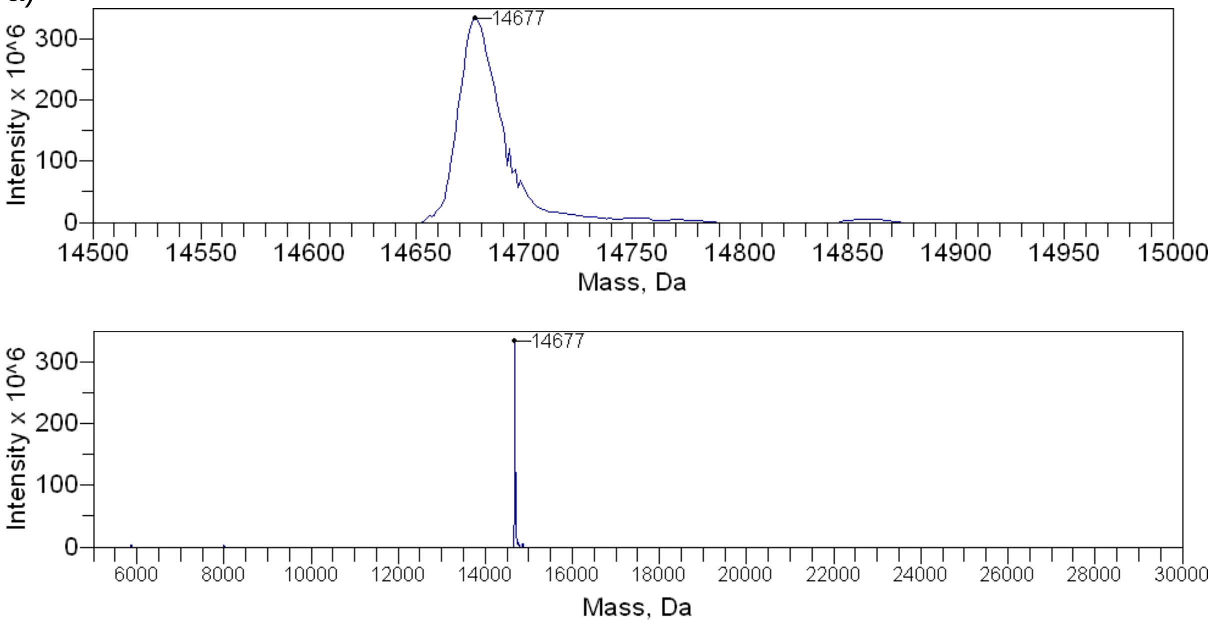

b)

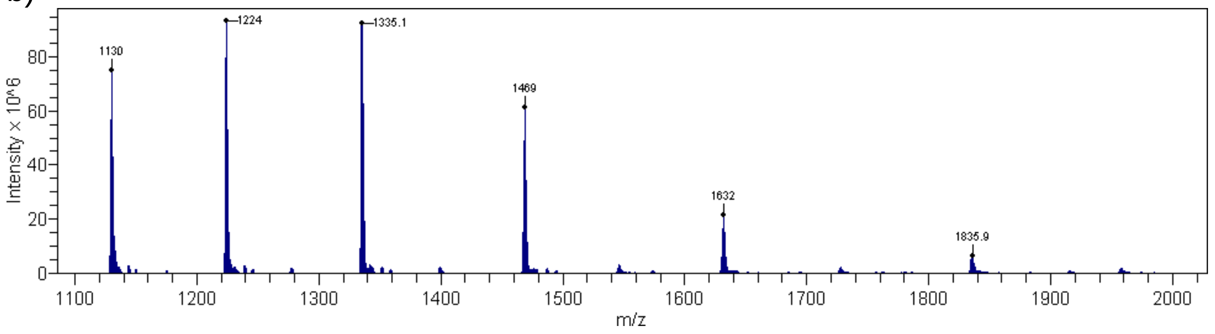

c)

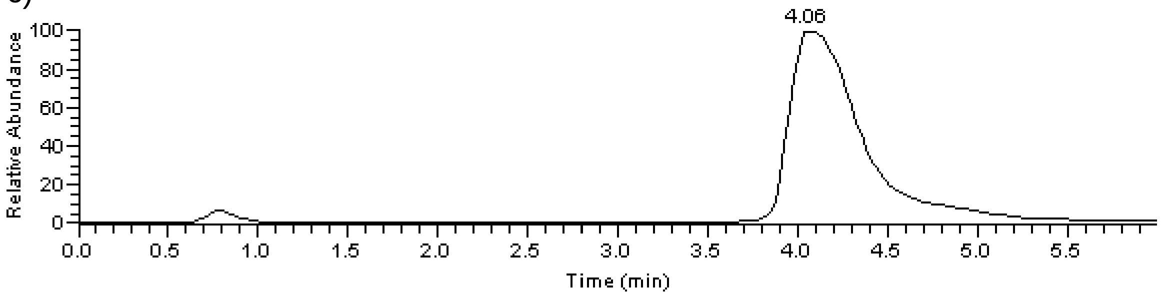

(a) deconvoluted, (b) non-deconvoluted, and (c) TIC mass spectrometry data for HER2DARPin(Cmut, L135C).

Supplementary Figure 50. HER2DARPin(Cmut, L135C) with 1 mM BrAcEGMe, 1 mM EDTA, in PBS pH 7.4, 10% DMF, 2 h, at 4 °C.
Expected mass (unmodified) 14676.4 Da
Expected mass (modified) 14791.6 Da

a)

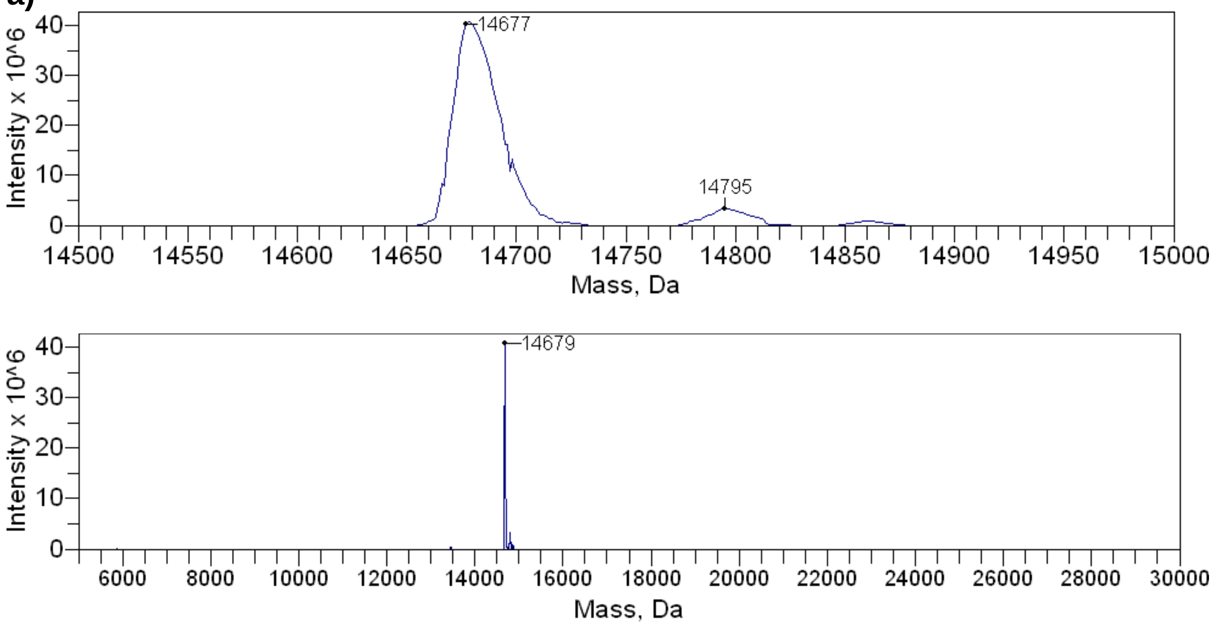

b)

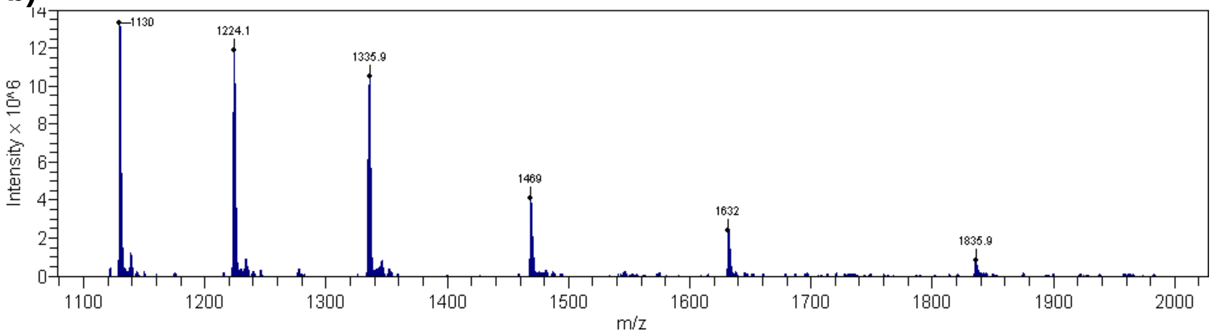

c)

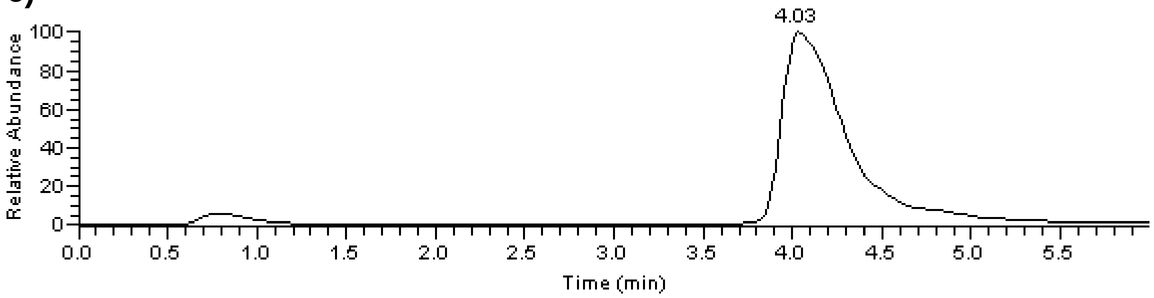

(a) deconvoluted, (b) non-deconvoluted, and (c) TIC mass spectrometry data for HER2DARPin(Cmut, L135C) reacted with BrAcEGMe.

Supplementary Figure 51. HER2DARPin(Cmut, L135C) with 1 mM NMM, 1 mM EDTA, in PBS pH 7.4, 10% DMF, 1 h, at 4 °C.
Expected mass (unmodified) 14676.4 Da
Expected mass (modified) 14787.5 Da

a)

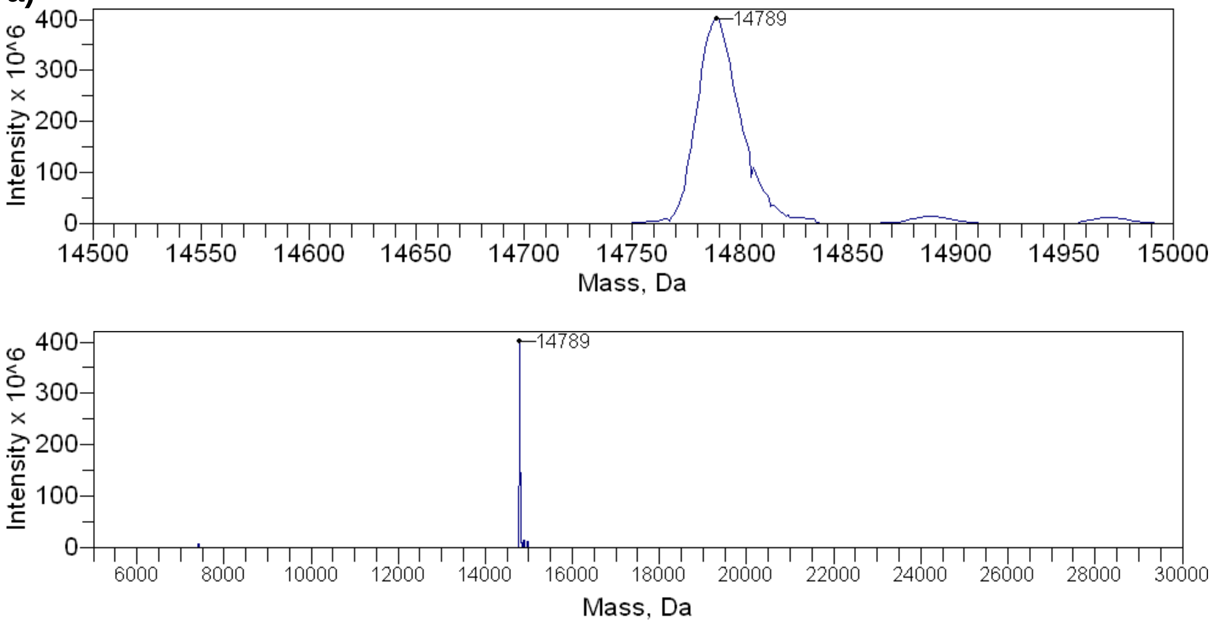

b)

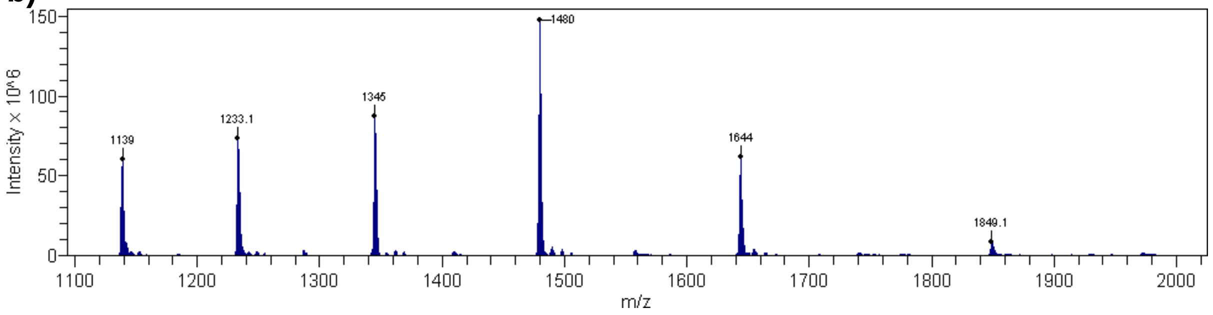

c)

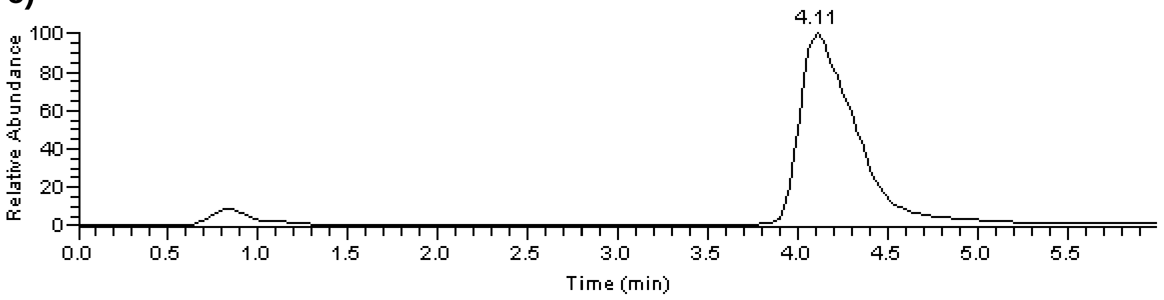

(a) deconvoluted, (b) non-deconvoluted, and (c) TIC mass spectrometry data for HER2DARPin(Cmut, L135C) reacted with NMM.

Supplementary Figure 52. HER2DARPin(N69C, D72C) in PBS, 1 mM EDTA. Expected mass 14573.4 Da

Sequence

MRGSHHHHHHGSDLGKKLLEAARAGQDDEVRILMANGADVNAKDEYGLTPLYLATAHGHLEI
VEVLLKCGACVNAVDAIGFTPLHLAAFIGHLEIAEVLLKHGADVNAQDKFGKTAFDISIGNG
NEDLAEILQKLN

a)

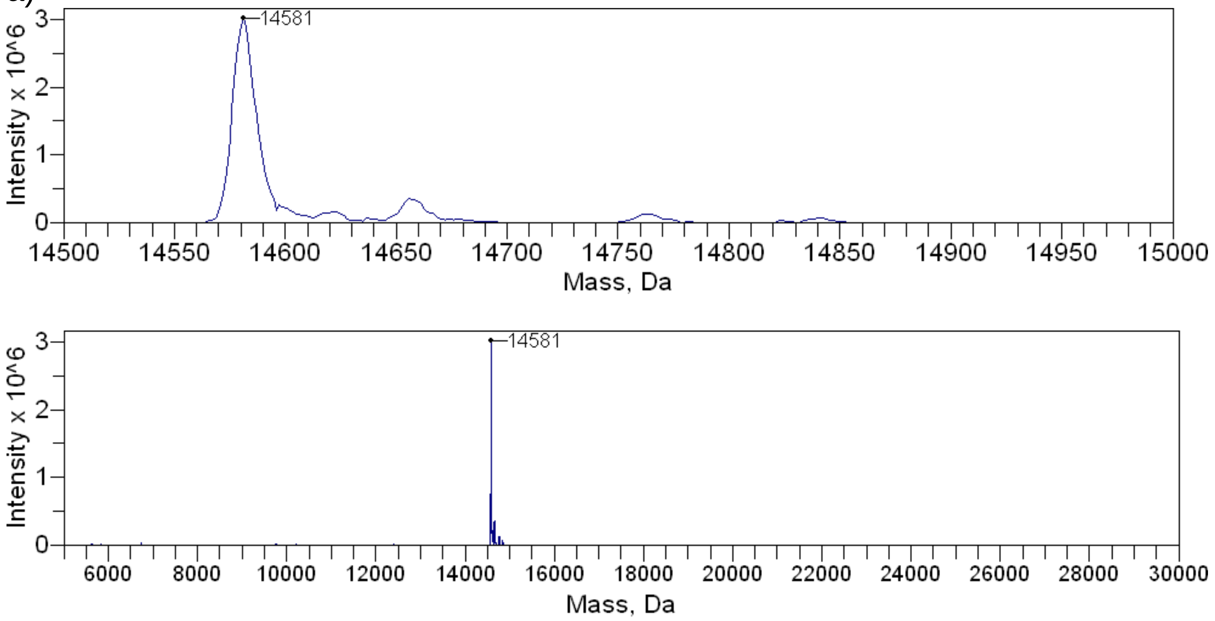

b)

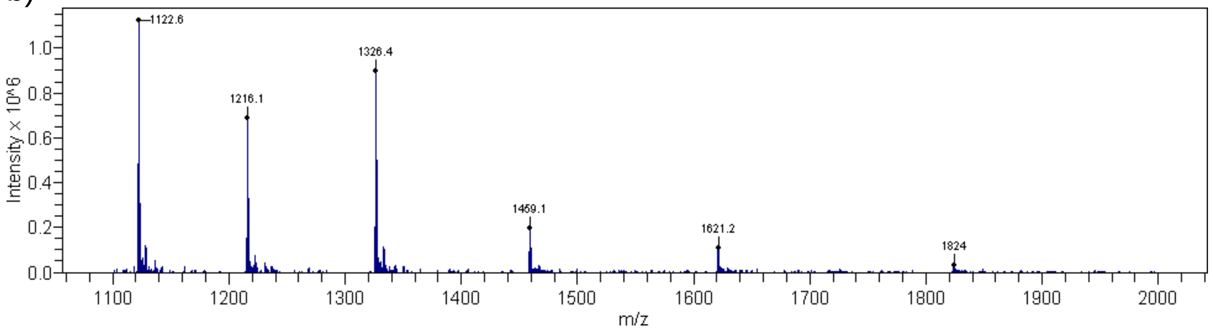

c)

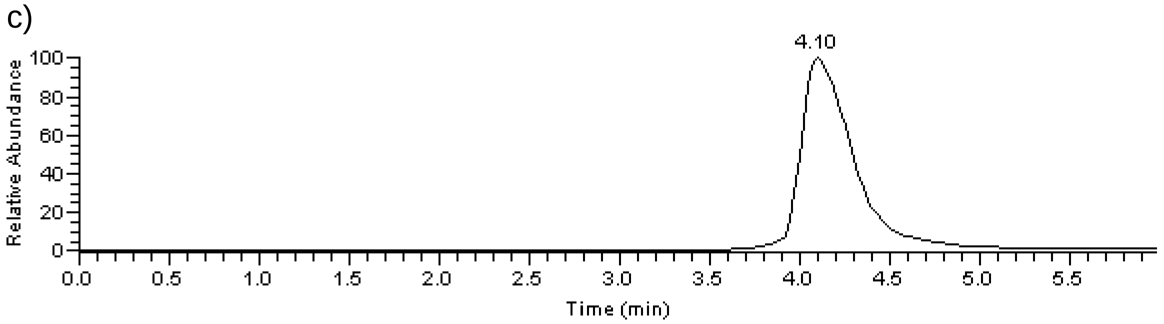

(a) deconvoluted, (b) non-deconvoluted, and (c) TIC mass spectrometry data for HER2DARPin(N69C, D72C).

Supplementary Figure 53. HER2DARPin(N69C, D72C) with 1 mM BrAcEGMe, 1 mM EDTA, in PBS pH 7.4, 10% DMF, 2 h, at 4 °C.

**Expected mass (unmodified)**	**14573.4 Da**
**Expected mass (singly modified)**	**14688.6 Da**
**Expected mass (doubly modified)**	**14803.8 Da**

a)

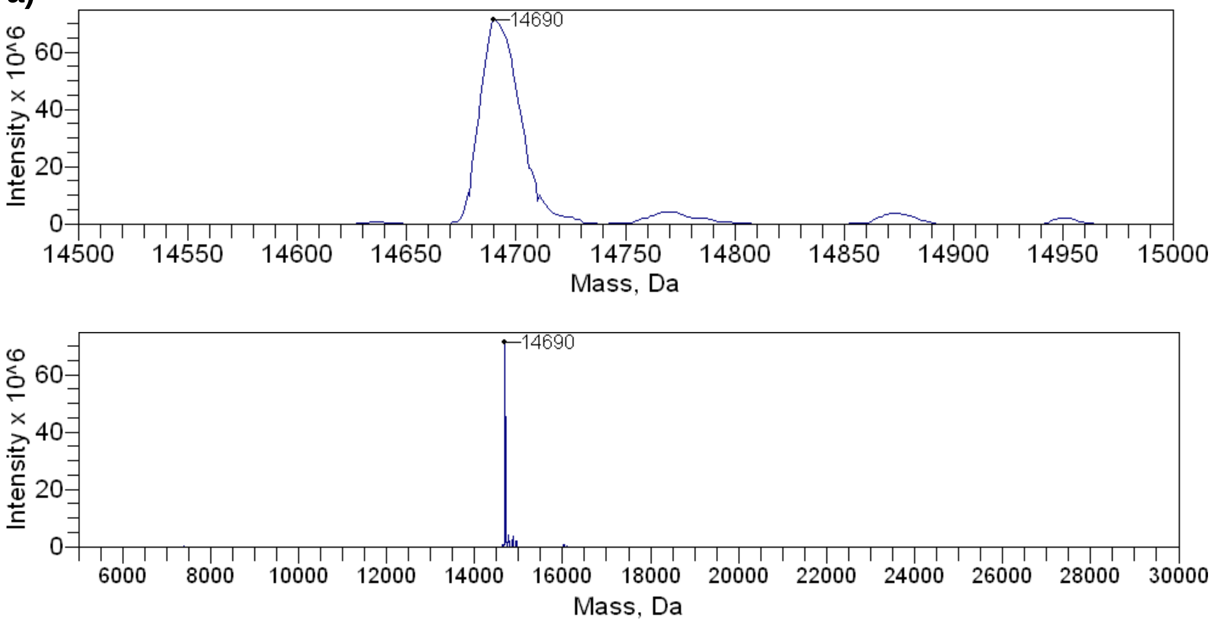

b)

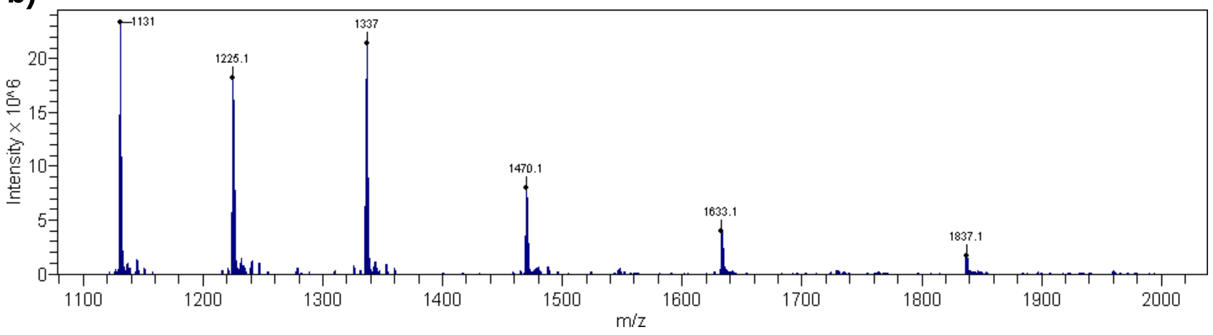

c)

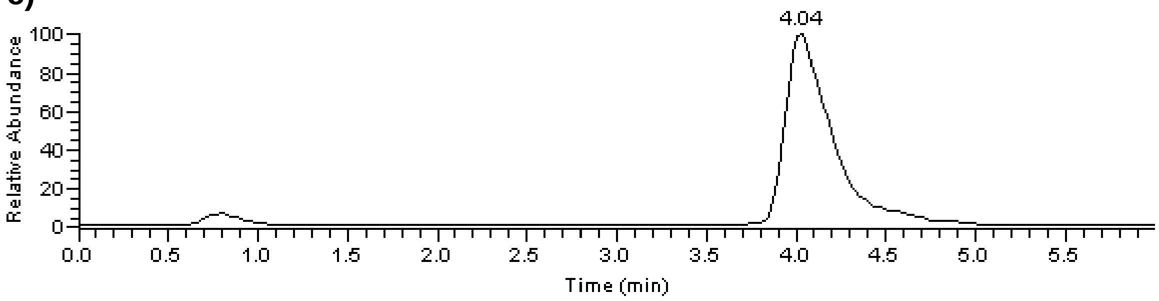

(a) deconvoluted, (b) non-deconvoluted, and (c) TIC mass spectrometry data for HER2DARPin(N69C, D72C) reacted with BrAcEGMe.

Supplementary Figure 54. HER2DARPin(N69C, D72C) with 1 mM NMM, 1 mM EDTA, in PBS pH 7.4, 10% DMF, 1 h, at 4 °C.

Expected mass (unmodified) 14573.4 Da
Expected mass (singly modified) 14684.5 Da
Expected mass (doubly modified) 14795.6 Da

a)

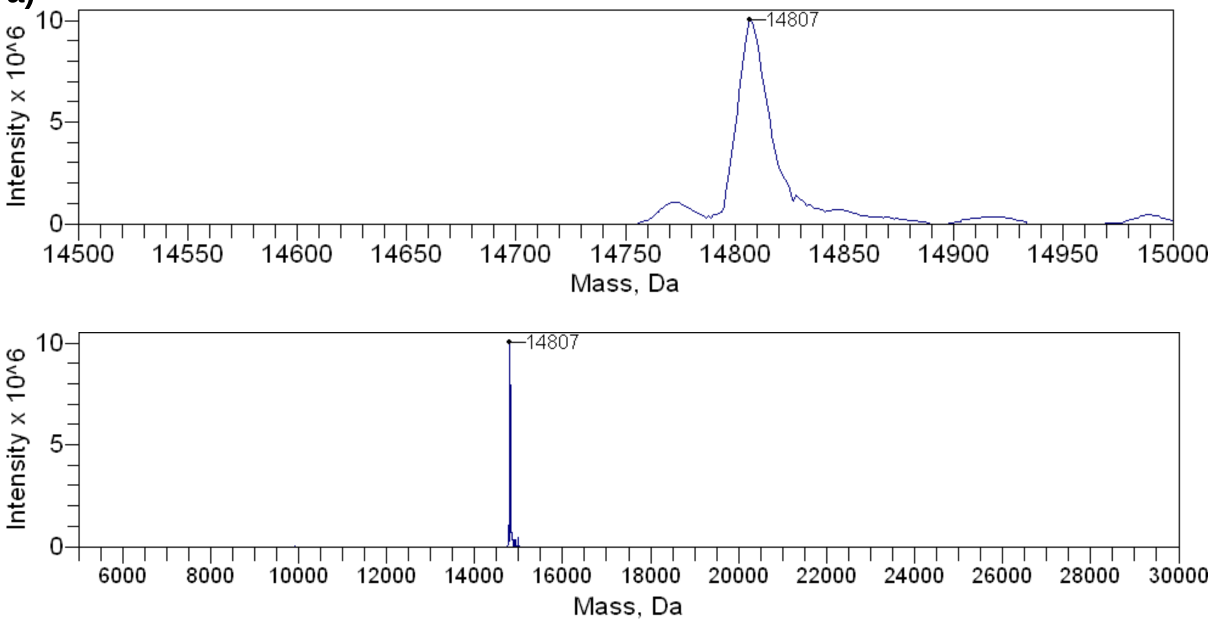

b)

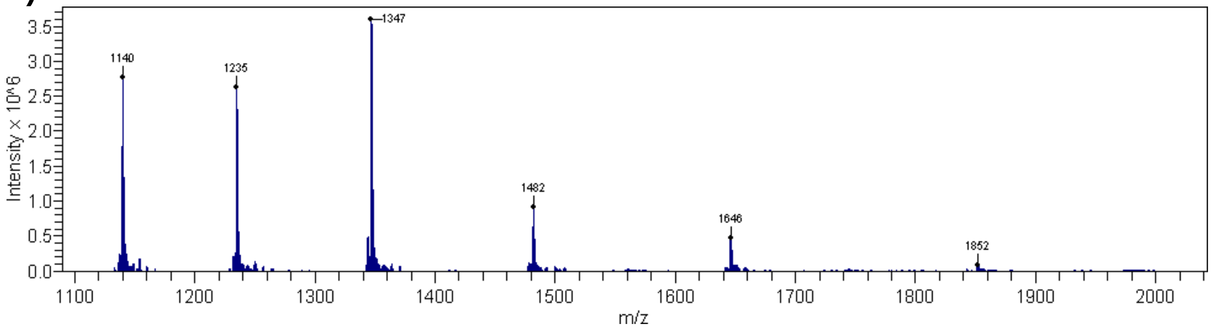

c)

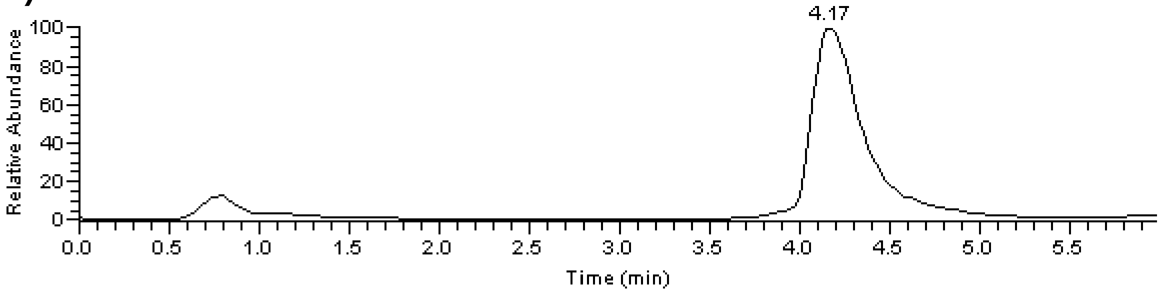

(a) deconvoluted, (b) non-deconvoluted, and (c) TIC mass spectrometry data for HER2DARPin(N69C, D72C) reacted with NMM.

Supplementary Figure 55. Mut4DARPin(N69C, D72C) in PBS, 1 mM EDTA. Expected mass 11133.4 Da

Sequence

MRGSHHHHHHGSDLGKKLLEAARAGQDDEVRILMANGADVNAKDKDGYTPLHLAAREGHL
EIVEVLLK**C**GA**C**VNAQDKFGKTAFDISIDNGNEDLAEILQKAA

a)

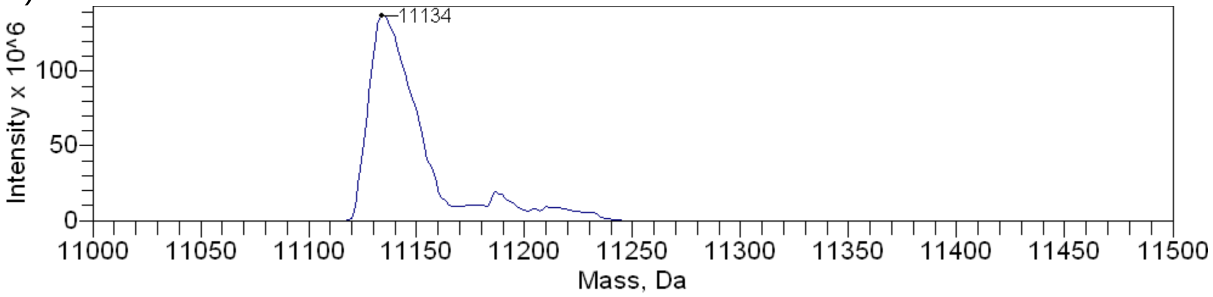

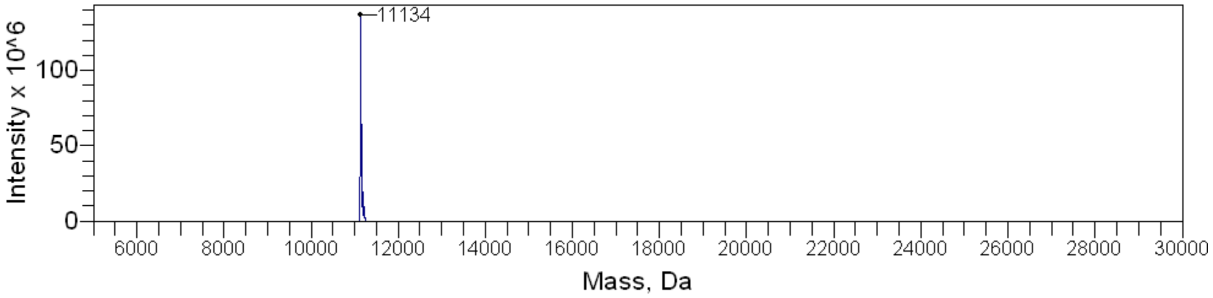

b)

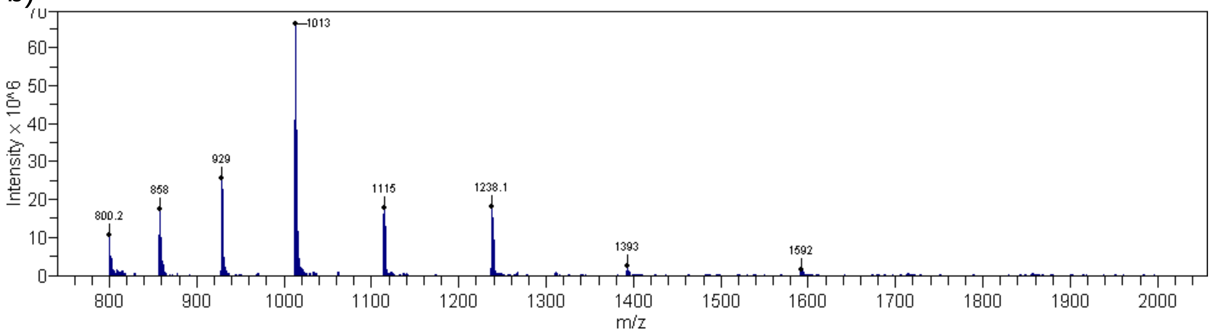

c)

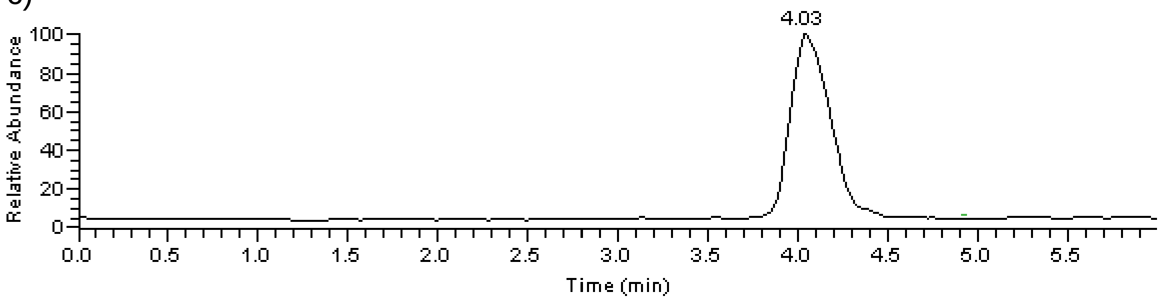

(a) deconvoluted, (b) non-deconvoluted, and (c) TIC mass spectrometry data for Mut4DARPin(N69C, D72C).

Supplementary Figure 56. Mut4DARPin(N69C, D72C) with 1 mM BrAcEGMe, 1 mM EDTA, in PBS pH 7.4, 10% DMF, 2 h, at 4 °C.

**Expected mass (unmodified)**	**11133.4 Da**
**Expected mass (singly modified)**	**11248.6 Da**
**Expected mass (doubly modified)**	**11363.8 Da**

a)

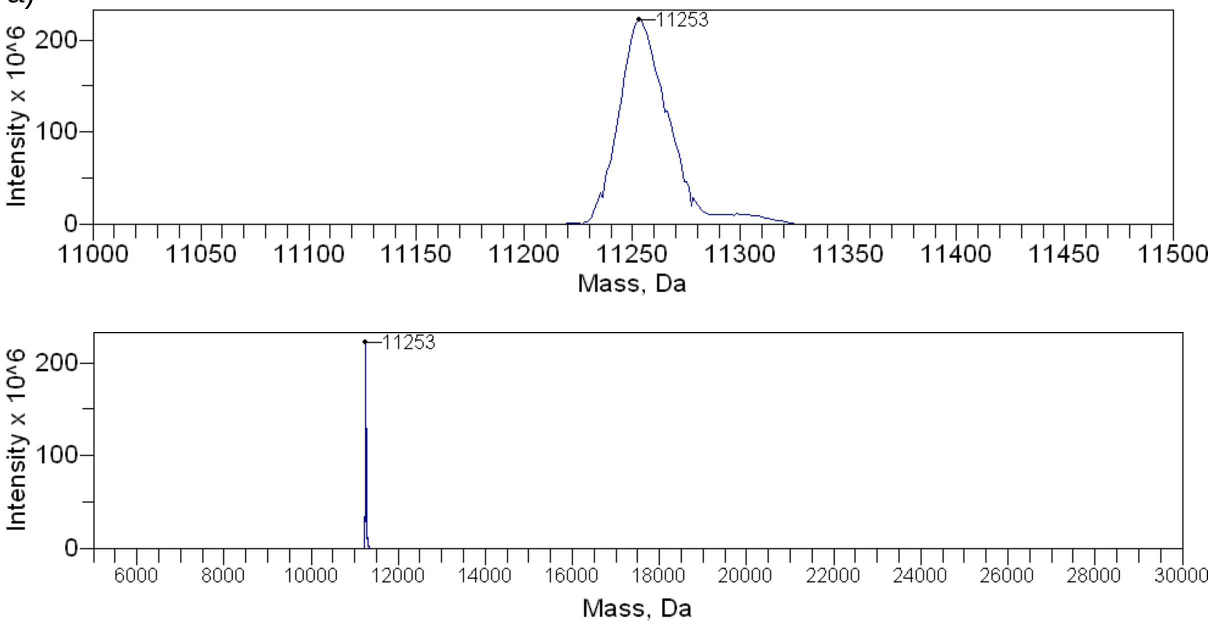

b)

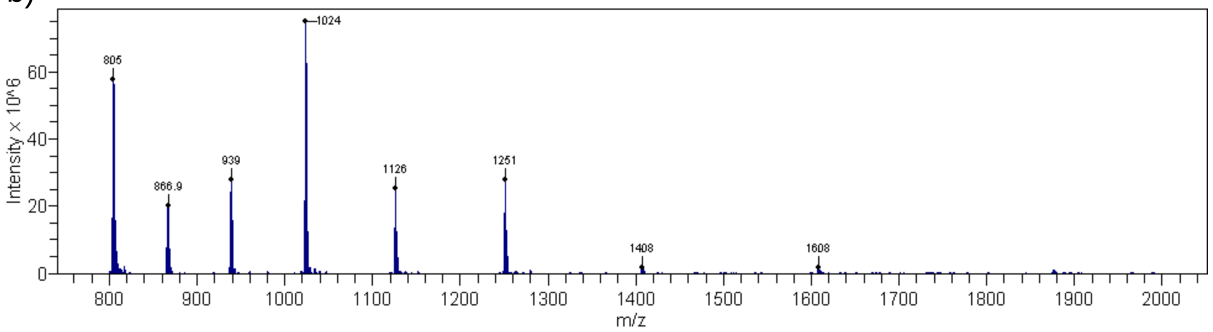

c)

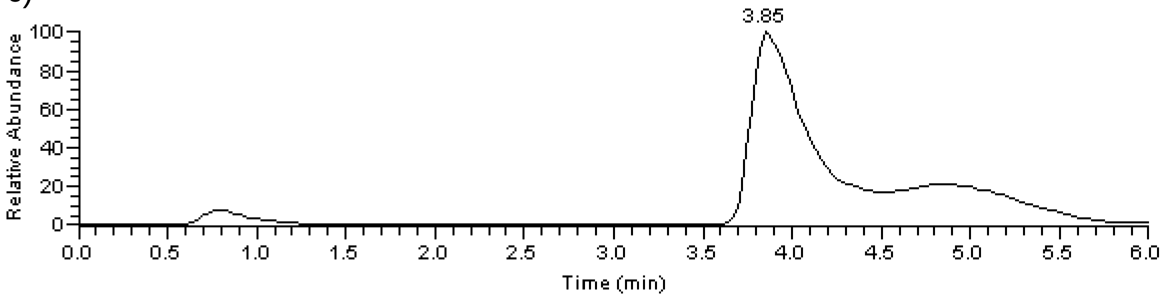

(a) deconvoluted, (b) non-deconvoluted, and (c) TIC mass spectrometry data for Mut4DARPin(N69C, D72C) reacted with BrAcEGMe.

Supplementary Figure 57. Mut4DARPin(N69C, D72C) with 1 mM NMM, 1 mM EDTA, in PBS pH 7.4, 10% DMF, 1 h, at 4 °C.

**Expected mass (unmodified)**	**11133.4 Da**
**Expected mass (singly modified)**	**11233.5 Da**
**Expected mass (doubly modified)**	**11355.6 Da**

a)

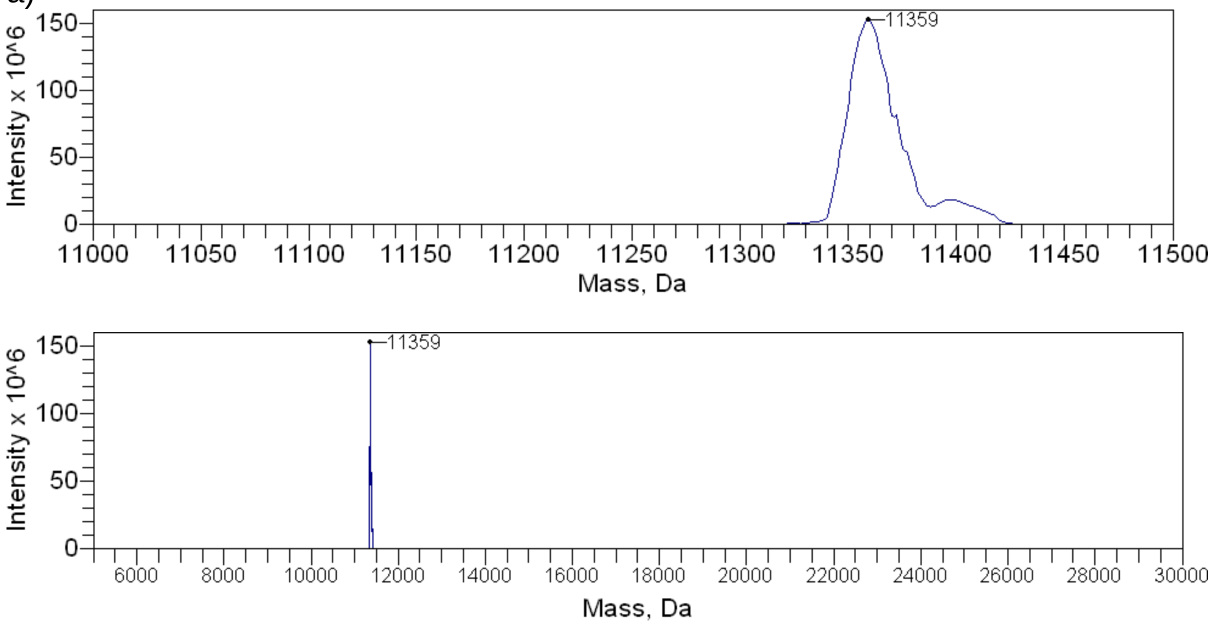

b)

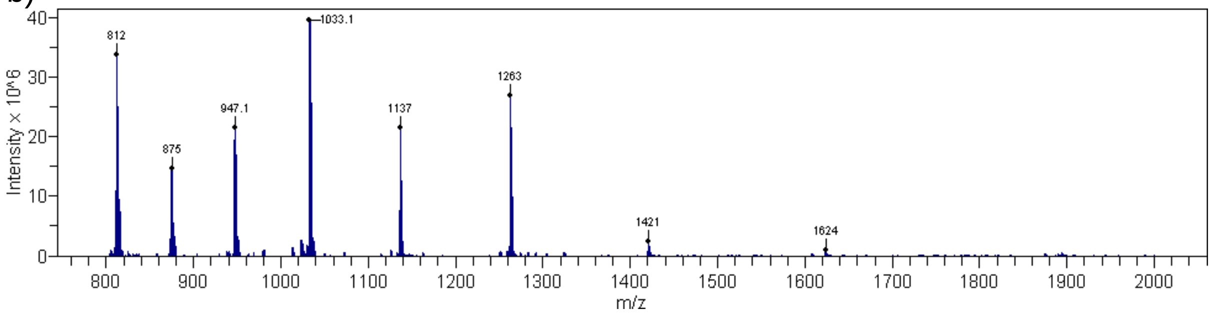

c)

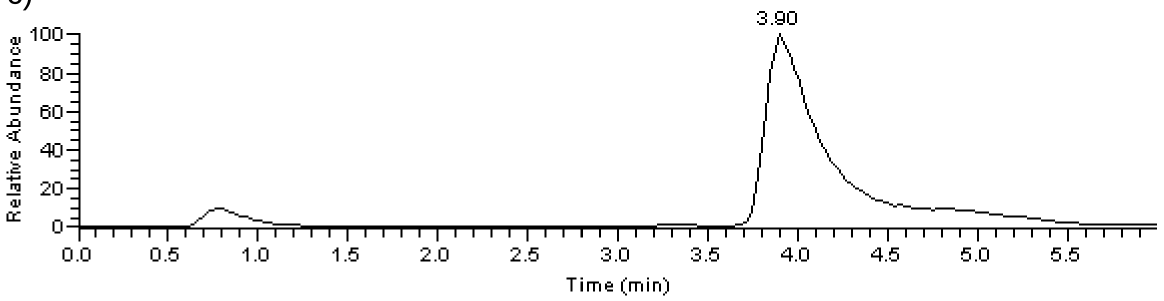

(a) deconvoluted, (b) non-deconvoluted, and (c) TIC mass spectrometry data for Mut4DARPin(N69C, D72C) reacted with NMM.

Supplementary Figure 58. HER2DARPin(N36C, D105C) in PBS, 1 mM EDTA. Expected mass 14573.5.4 Da

Sequence
MRGSHHHHHHGSDLGKKLLEAARAGQDDEVRILMA<u>**C**</u>GADVNAKDEYGLTPLYLAT
AHGHLEIVEVLLKNGADVNAVDAIGFTPLHLAAFIGHLEIAEVLLKHGA<u>**C**</u>VNAQDKFG
KTAFDISIGNGNEDLAEILQKLN

a)

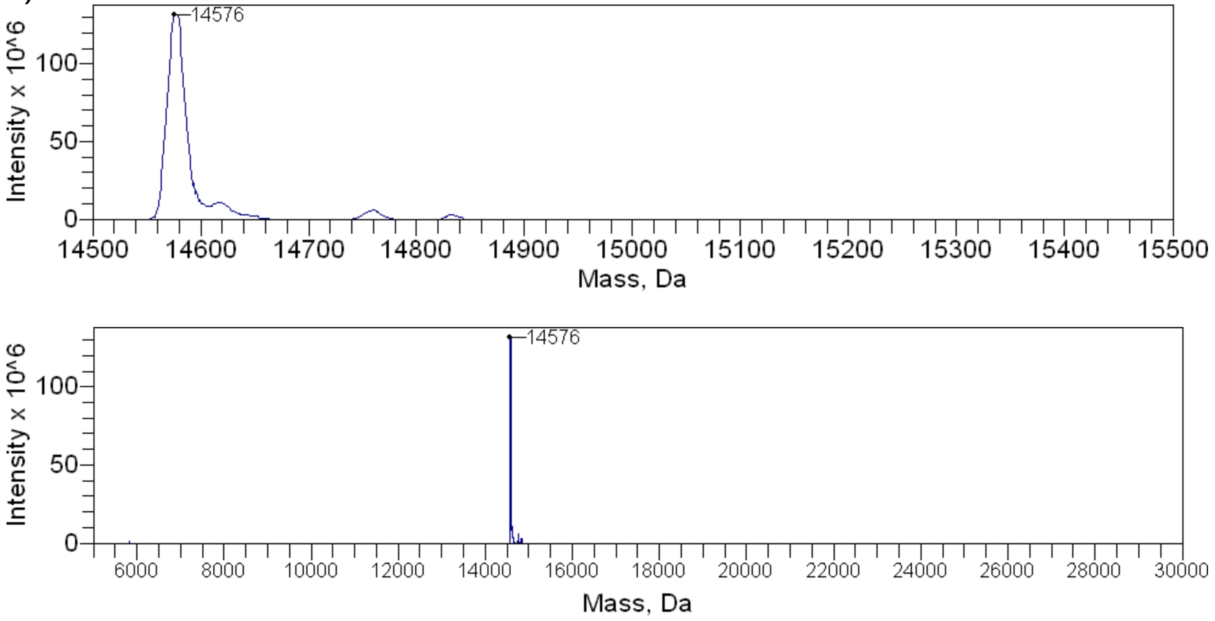

b)

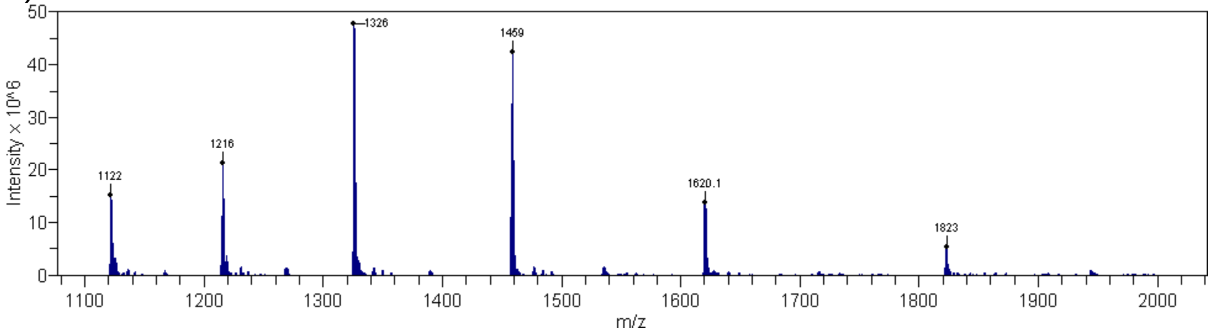

c)

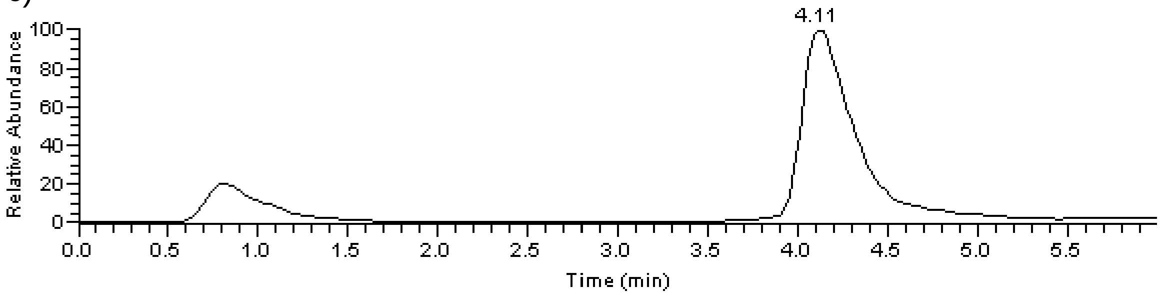

(a) deconvoluted, (b) non-deconvoluted, and (c) TIC mass spectrometry data for HER2DARPin(N36C, D105C).

Supplementary Figure 59. HER2DARPin(N36C, D105C) with 1 mM BrAcEGMe, 1 mM EDTA, in PBS pH 7.4, 10% DMF, 2 h, at 4 °C.
Expected mass (unmodified) 14573.4 Da
Expected mass (singly modified) 14688.6 Da
Expected mass (doubly modified) 14803.8 Da

a)

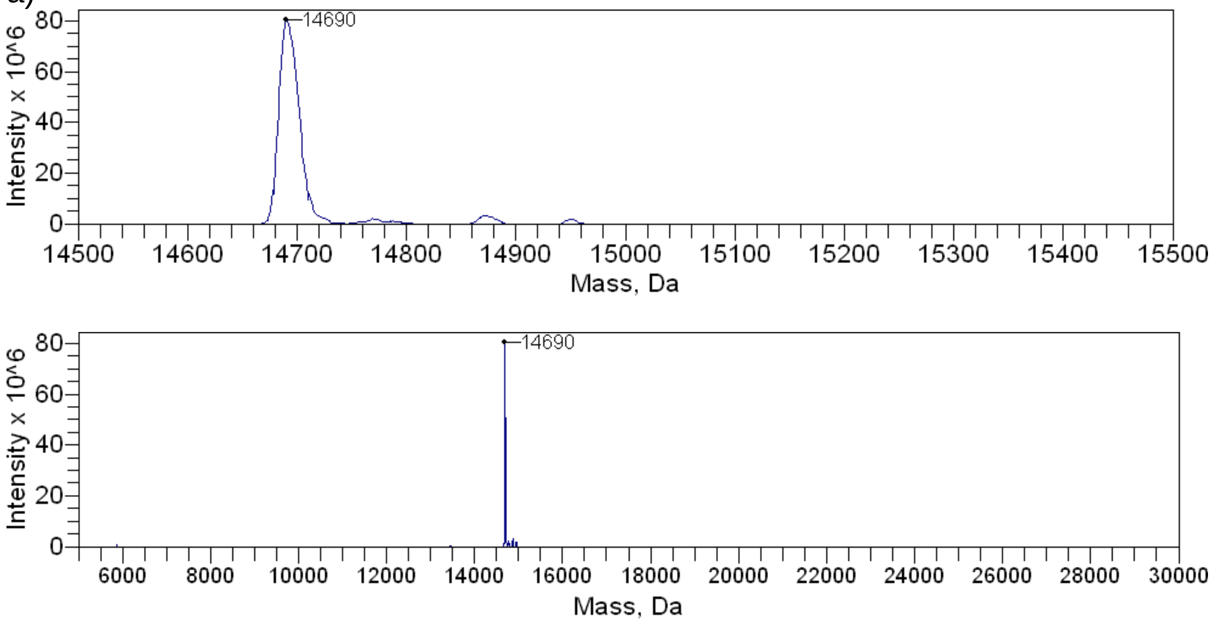

b)

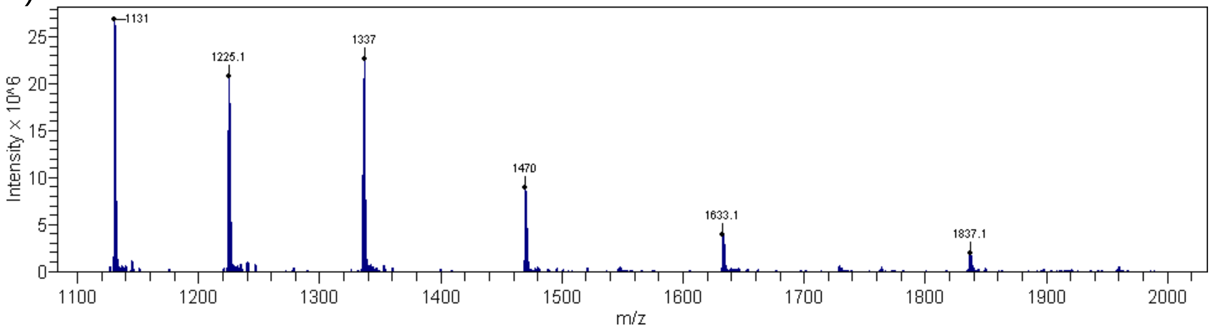

c)

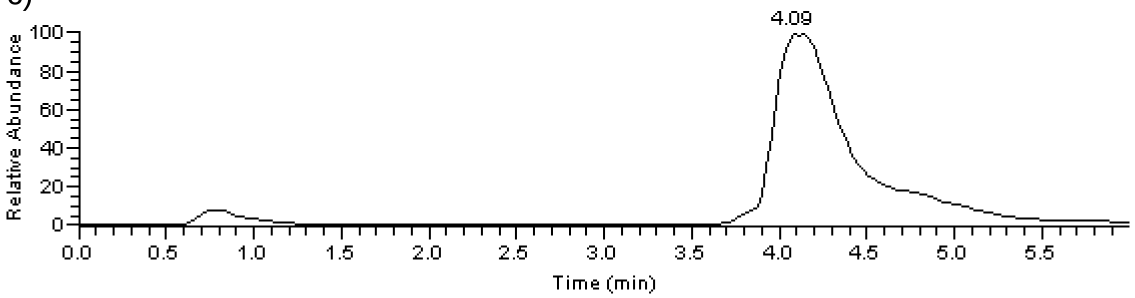

(a) deconvoluted, (b) non-deconvoluted, and (c) TIC mass spectrometry data for HER2DARPin(N36C, D105C) reacted with BrAcEGMe.

Supplementary Figure 60. HER2DARPin(N36C, D105C) with 1 mM NMM, 1 mM EDTA, in PBS pH 7.4, 10% DMF, 1 h, at 4 °C.

**Expected mass (unmodified)**	**14573.4 Da**
**Expected mass (singly modified)**	**14684.5 Da**
**Expected mass (doubly modified)**	**14795.6 Da**

a)

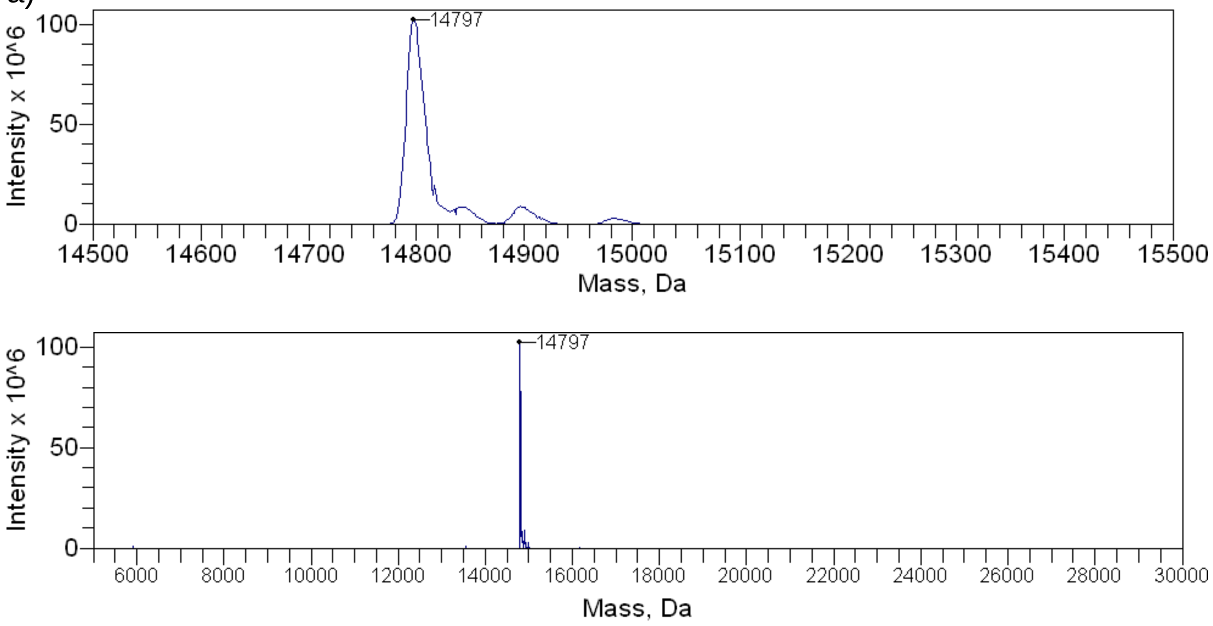

b)

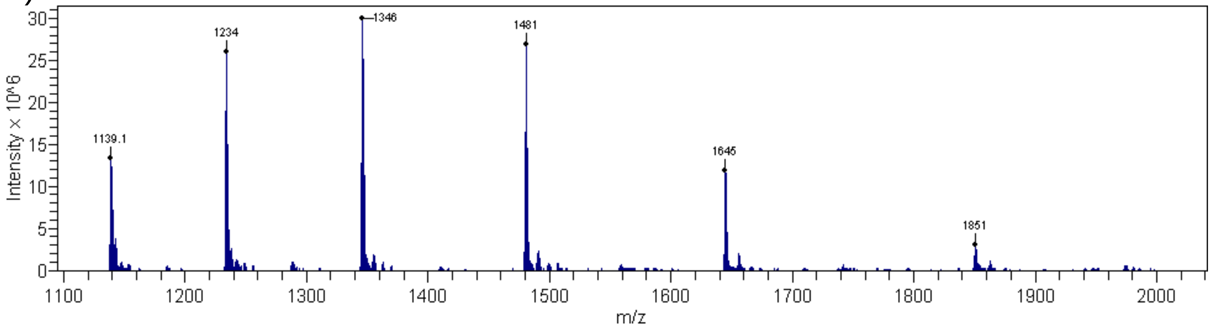

c)

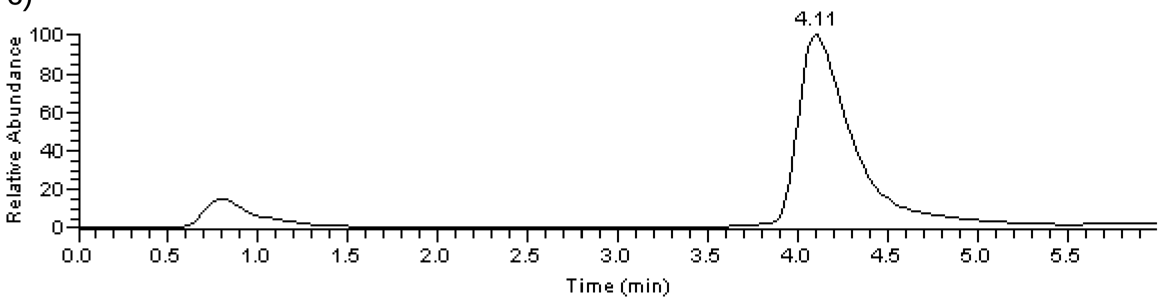

(a) deconvoluted, (b) non-deconvoluted, and (c) TIC mass spectrometry data for HER2DARPin(N36C, D105C) reacted with NMM.

Supplementary Figure 61. HER2DARPin(N36C, D105CAcEGMe) with 1 mM TMRM, 1 mM EDTA, in PBS pH 7.4, 10% DMF, 1 h, at 4 °C.

Expected mass (unreacted) 14688.6 Da

Expected mass (reacted) 15241.2 Da

a)

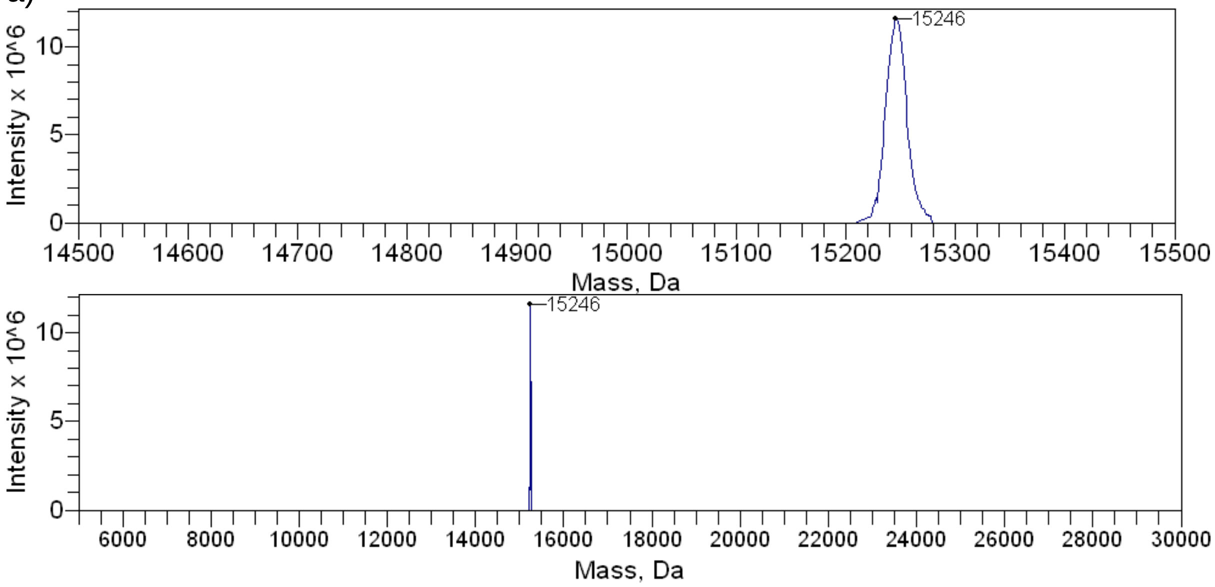

b)

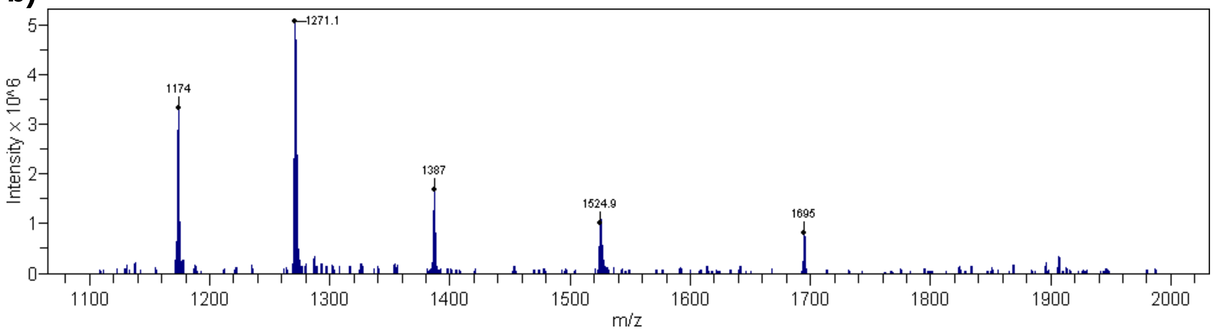

c)

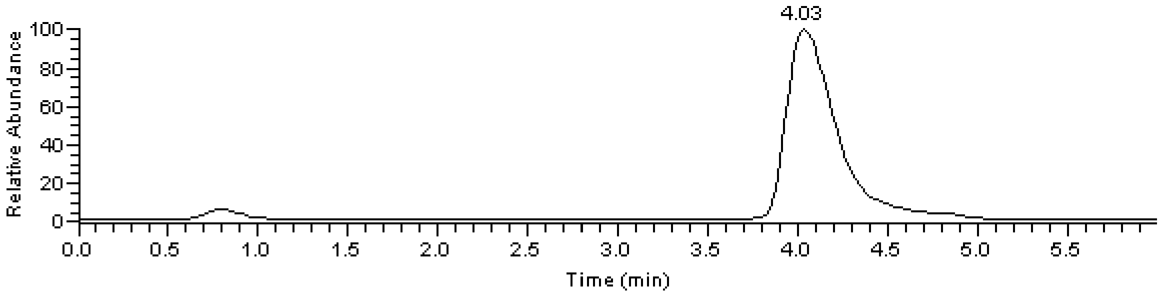

(a) deconvoluted, (b) non-deconvoluted, and (c) TIC mass spectrometry data for HER2DARPin(N36C, D105CAcEGMe) reacted with TMRM to form HER2DARPin(N36CTMRM, D105CAcEGMe).

Supplementary Figure 62. HER2DARPin(N69C, D72CAcEGMe) with 1 mM NMM, 1 mM EDTA, in PBS pH 7.4, 10% DMF, 1 h, at 4 °C.
Expected mass (unreacted) 14688.6 Da
Expected mass (reacted) 14799.7 Da

a)

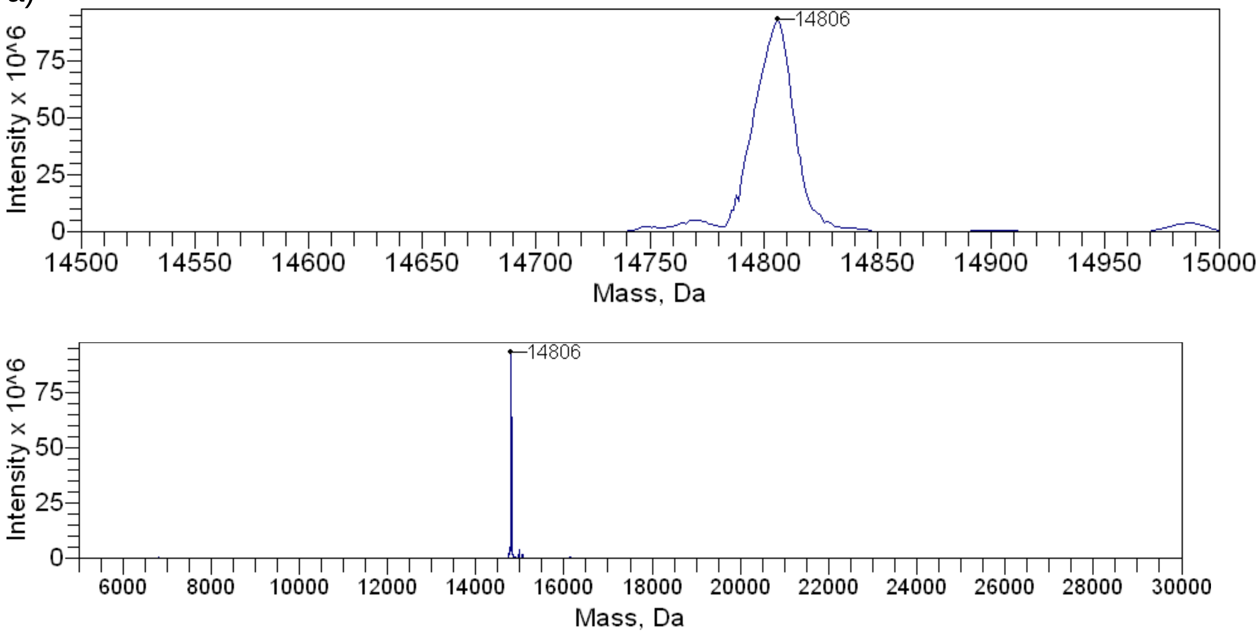

b)

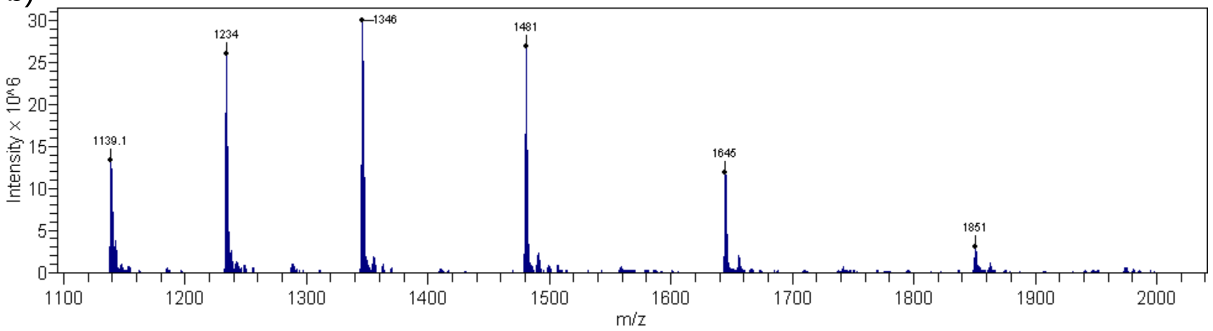

c)

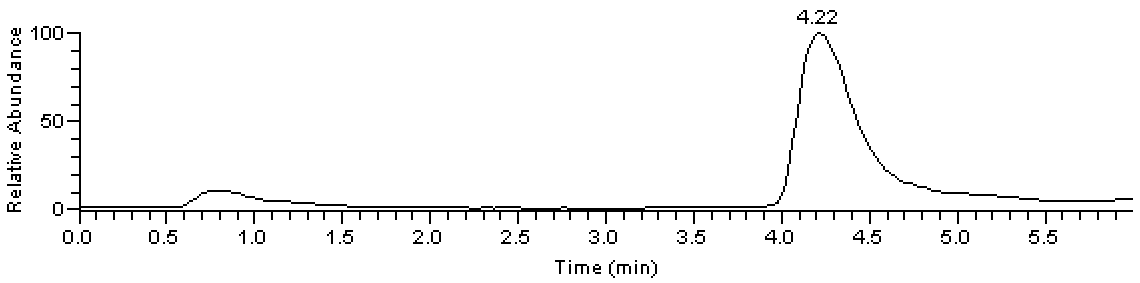

(a) deconvoluted, (b) non-deconvoluted, and (c) TIC mass spectrometry data for HER2DARPin(N69C, D72CAcEGMe) reacted with NMM to form HER2DARPin(N69CNMM, D72CAcEGMe).

Supplementary Figure 63. Mut4DARPin(N69C, D72CAcEGMe) with 1 mM NMM, 1 mM EDTA, in PBS pH 7.4, 10% DMF, 1 h, at 4 °C.

**Expected mass (unreacted)**	**11248.6 Da**
**Expected mass (reacted)**	**11359.7 Da**

a)

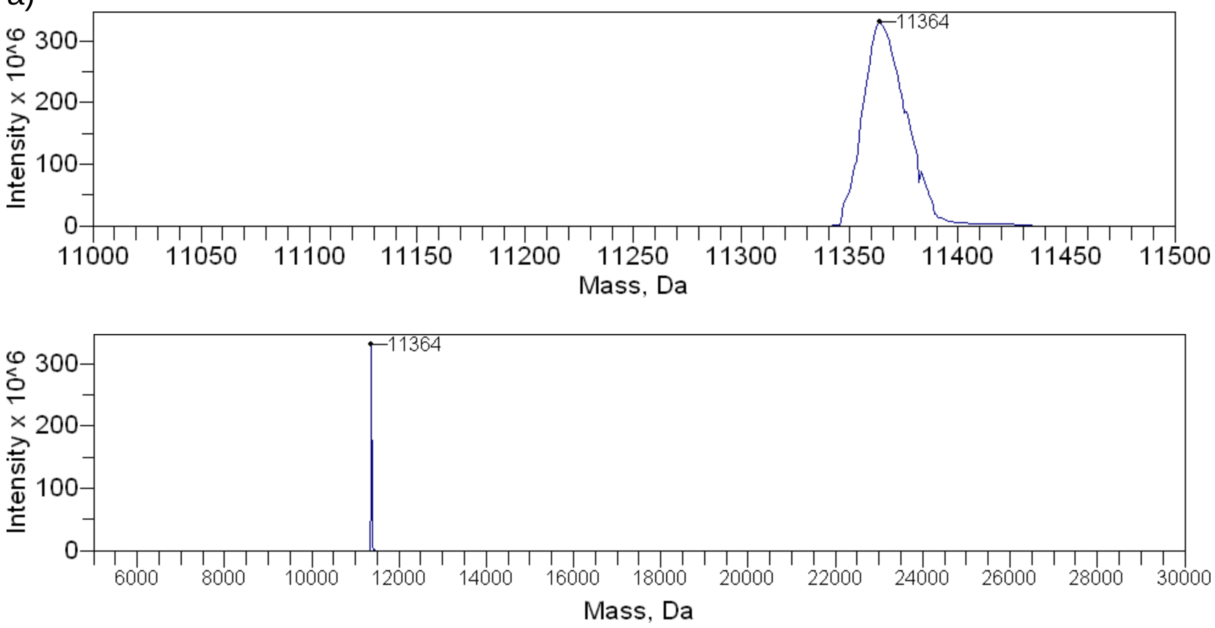

b)

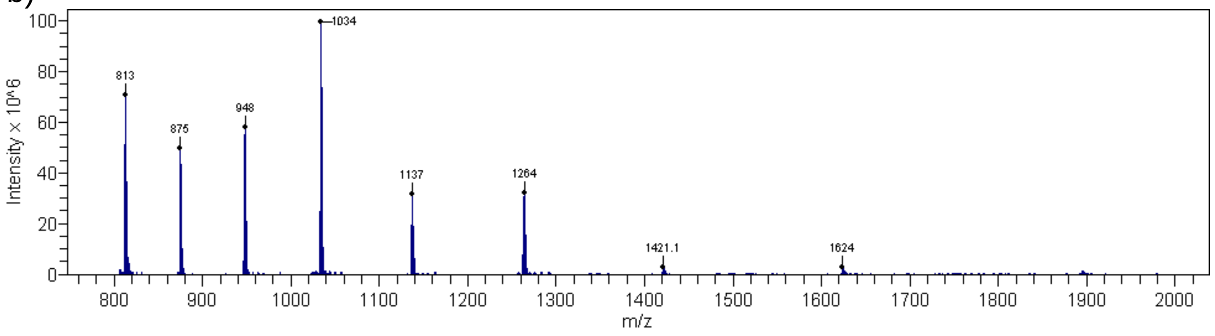

c)

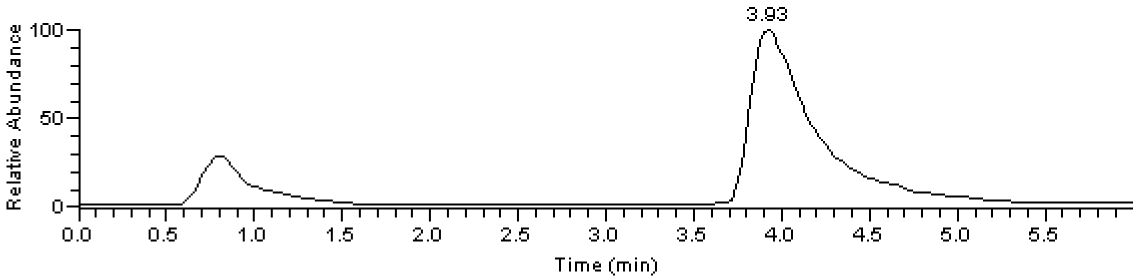

(a) deconvoluted, (b) non-deconvoluted, and (c) TIC mass spectrometry data for Mut4DARPin(N69C, D72CAcEGMe) reacted with NMM to form Mut4DARPin(N69CNMM, D72CAcEGMe).

Circular Dichroism and Thermal Unfolding

a)

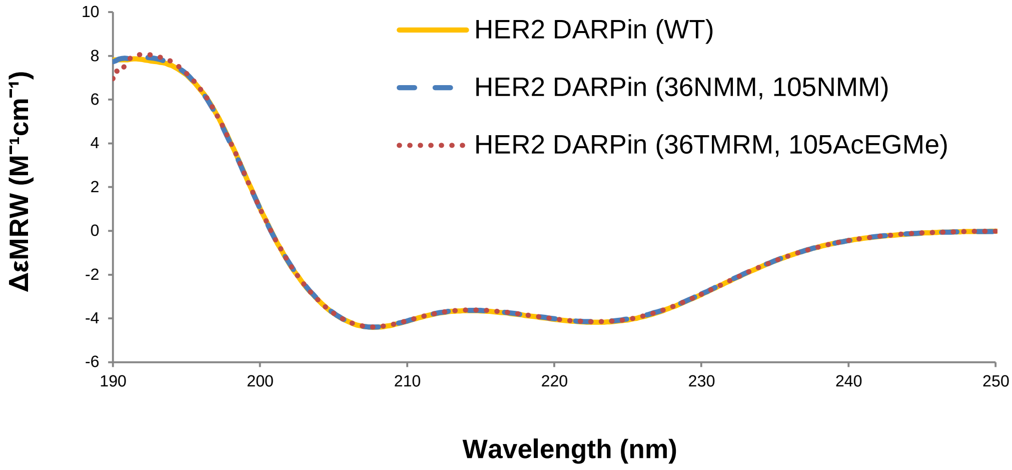

b)

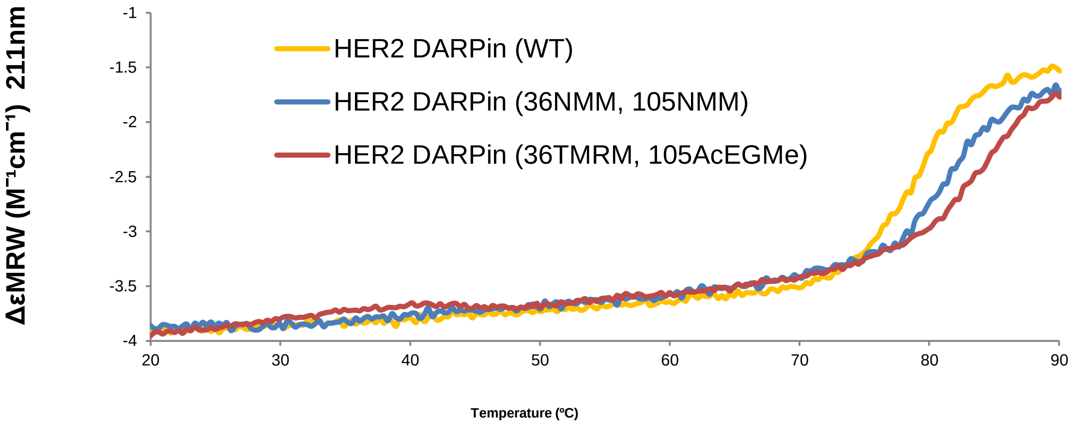

(a) Circular dichroism spectra of HER2DARPin variants, and (b) thermal unfolding of HER2DARPin variants.
